# SOMOphilic alkyne vs radical-polar crossover approaches: The full story of the azido-alkynylation of alkenes

**DOI:** 10.3762/bjoc.20.64

**Published:** 2024-04-03

**Authors:** Julien Borrel, Jerome Waser

**Affiliations:** 1 Laboratory of Catalysis and Organic Synthesis, Institute of Chemical Sciences and Engineering, Ecole Polytechnique Fédérale de Lausanne, EPFL SB ISIC LCSO, BCH 4306, 1015 Lausanne, Switzerland

**Keywords:** alkyne, azide, hypervalent iodine, photoredox, trifluoroborate salt

## Abstract

We report the detailed background for the discovery and development of the synthesis of homopropargylic azides by the azido-alkynylation of alkenes. Initially, a strategy involving SOMOphilic alkynes was adopted, but only resulted in a 29% yield of the desired product. By switching to a radical-polar crossover approach and after optimization, a high yield (72%) of the homopropargylic azide was reached. Full insights are given about the factors that were essential for the success of the optimization process.

## Introduction

**Scheme 1:**
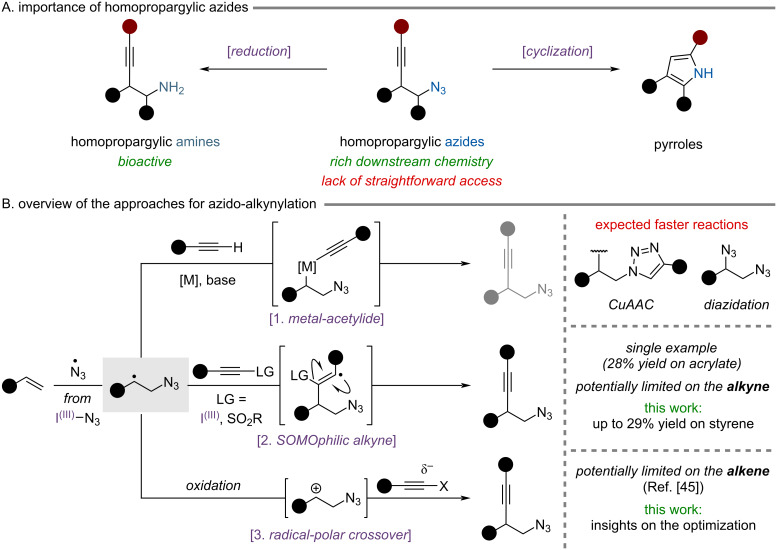
Overview of homopropargylic azides importance and strategies for azido-alkynylation.

Homopropargylic azides are important building blocks bearing two of the most versatile functional groups, allowing a rich panel of functionalization. They have been used as intermediates in numerous syntheses to access bioactive compounds [1–4] or materials [5–7]. In addition, azide reduction affords homopropargylic amines, which can be found in bioactive molecules and have been tested in structure–activity relationship studies (Scheme 1A) [8–10]. Moreover, transformations have been developed to exploit the two functional groups simultaneously, for example through their intramolecular cyclization to form pyrroles in the presence of transition metal catalysts [11–14].

Currently, this motif is synthesized by sequential introduction of the two functional groups [11–13]. Addition of a lithium acetylide to an epoxide affords the corresponding homopropargylic alcohol which can then undergo a sequence of mesylation and substitution with azide ions to produce the desired homopropargylic azide. However, this approach gives only access to products bearing the alkyne at the least substituted position. To the best of our knowledge, no general strategy has been employed to access the other regioisomer possessing a terminal azide, despite its implication in the synthesis of complex molecules [3,6]. Therefore, the development of a straightforward reaction to synthesize homopropargylic azides would be of general interest.

The azido-alkynylation of alkenes would allow to generate the desired motif in a single step, greatly increasing the molecular complexity of the starting substrate. Using radical chemistry would lead to a regioselective addition of azide radicals to the alkene, forming selectively the most stabilized C-centered radical. A prominent method for the generation of azide radicals relies on hypervalent iodine reagents [15–16]. Azidobenziodoxolone, also known as Zhdankin reagent, has often been used under thermal or photochemical conditions to generate the desired azide radical in a controlled fashion. However, recent safety issues arising from the shock and impact sensitivity of the compound led to the development of the azidobenziodazolone scaffold [17]. This class of derivative showed an improved safety profile while retaining the redox properties of the original reagent.

A single example of azido-alkynylation has been reported by Ramasastry and co-workers during a mechanistic study for an azido-hydration reaction [18]. The homopropargylic azide was obtained in only 28% yield using phenyl vinyl ketone. Based on reported aza-alkynylation reactions [19–23] and modern azidation methods using radical chemistry [17,24–26] three approaches could be envisaged. All of them would initially involve the addition of azide radicals to an alkene, generating a carbon-centered radical. Then, different trapping of this intermediate could be performed (Scheme 1B).

First, C-centered radicals are known to recombine with metal-acetylides, in particular copper [27]. Reductive elimination of the organometallic intermediate would lead to the desired product (Scheme 1B, reaction 1). Unfortunately, this approach will not be compatible in the case of azidation since the copper, azides and alkynes present in the mixture are expected to undergo alkyne–azide cycloaddition reactions [28]. Moreover, different azide sources are known to efficiently promote the diazidation of alkenes in the presence of a copper catalyst, often proceeding via a radical mechanism [24,29–31].

A second approach would involve SOMOphilic alkynes to trap the radical by a purely open-shell mechanism (Scheme 1B, reaction 2). Two classes of reagents are commonly used: ethynylbenziodoxolones (EBXs) [32–33] and alkynylsulfones [34]. A potential limitation of this method lies in the substitution of the transferred alkyne. The efficiency of the radical addition to those reagents is known to be highly dependent on the alkyne substituent. Arylalkynes are expected to perform well but in multiple cases alkyl substituents were reported to afford low yields or no reaction [35–38].

Finally, a radical-polar crossover (RPC) approach could be envisaged [39–40]. Instead of attempting to trap the C-centered radical, further oxidation would generate the corresponding carbocation, which upon reaction with a nucleophilic alkyne would form the product (Scheme 1B, reaction 3). Based on precedence in the literature, this method should allow to transfer efficiently both aryl- and alkyl-substituted alkynes [41–44]. On the other hand, the nature of the alkene might be limited as it would strongly influence the oxidation potential of the carbon radical and the stability of the resulting carbocation. Recently, we reported the first successful application of an RPC strategy for the azido-alkynylation of styrenes [45].

Herein, we describe our initial effort towards developing an azido-alkynylation of alkenes using the SOMOphilic alkyne approach instead. Then, the optimization of the RPC strategy will be discussed in detail, giving insights into the different steps of the optimization, which were available only as raw data in our previous work [45].

## Results and Discussion

### SOMOphilic alkynes

We started to investigate the azido-alkynylation of styrene (**1a**) using EBX reagent **2** as SOMOphilic alkyne (Table 1). Tosyl-azidobenziodazolone (Ts-ABZ, **3**), previously developed by our group [17], was selected as an azide source. Upon light irradiation, it can release an azide radical by homolysis of the I−N_3_ bond [46]. We were pleased to see that irradiation of a mixture of styrene (**1a**), Ph-EBX (**2**) and Ts-ABZ (**3**) afforded 17% isolated yield of the desired homopropargylic azide **4a** (Table 1, entry 1). Heating the reaction to 80 °C instead of using light to form the radical only afforded traces of the product (Table 1, entry 2). Changing the solvent to DCE slightly increased the yield (Table 1, entry 3). Blue light with an emission spectrum centered around 467 nm was initially selected since Ph-EBX is known to absorb light of lower wavelength, which is expected to cause degradation [47]. Indeed, when the reaction was carried out using 440 nm blue light a lower yield of 10% was obtained and full conversion of the EBX reagent was observed (Table 1, entry 4). Next, we wanted to test different additives in the transformation in an attempt to increase the yield. Addition of acetoxybenziodoxolone (AcOBX) has previously been reported to help initiating the degradation of Ts-ABZ (**3**) to the azidyl radical [46]. In our case, no difference in yield was observed (Table 1, entry 5). Currently, the reaction is expected to generate a large quantity of iodanyl radical from Ts-ABZ (**3**) homolysis and from the addition–elimination on Ph-EBX (**2**). Since no quencher is present in the mixture, we wondered if the accumulation of those radicals could be responsible for the low yields obtained. Addition of (TMS)_3_SiH, a H^•^ donor, suppressed the reaction by reducing Ph­EBX (**2**) (Table 1, entry 6). Using diisopropyl ether as a milder donor had no effect on the reaction (Table 1, entry 7). Next, we tested reducing agents expected to react with the iodanyl radical. The addition of ʟ-ascorbic acid led to no improvement of yield and Hantzsch ester suppressed the formation of the desired product (Table 1, entries 8 and 9). Carrying out the reaction in the presence of sodium formate, which can play a dual role of H^•^ donor and reductant [48–49], only led to a decreased of Ph-EBX (**2**) conversion, along with a diminished yield (Table 1, entry 10). The addition of DABCO [18] or TBAI [50], two additives known to activate azidobenziodoxolone (ABX), afforded complex mixtures with no trace of **4a** (Table 1, entry 11). Acids or fluorinated alcohols were tested to activate the different hypervalent iodine reagents. While AcOH, TFA and TFE had no impact on the reaction (Table 1, entry 12), the presence of 1.5 equivalents of HFIP slightly improved the yield (Table 1, entry 13). Increasing the amount of styrene in the reaction had no impact (Table 1, entry 14), highlighting that the issue might come from an inefficient trapping of the C-centered radical by Ph-EBX (**2**) and not from a sluggish addition of azide radicals to the double bond. We attempted to solve this issue by using Ts-ABZ (**3**) in excess, which should increase the overall quantity of carbon radicals formed, doing so slightly improved the yield of **4a** to 29% with no styrene remaining (Table 1, entry 15).

**Table 1:** Optimization of the azido-alkynylation using Ph-EBX.

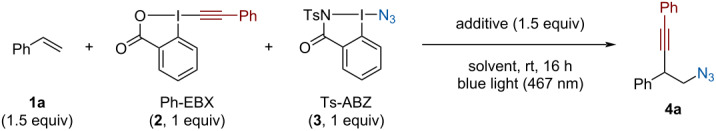				

Entry	Solvent	Additive	Equiv variation	Yield **4a** (%)^a^

1	CH_3_CN	–	–	(17)
2^b^	CH_3_CN	–	–	<5
3	DCE	–	–	19
4^c^	DCE	–	–	10
5	DCE	AcOBX		18
6	DCE	(TMS)_3_SiH	–	–
7	DCE	(iPr)_2_O	–	16
8	DCE	ʟ-ascorbic acid	–	16
9	DCE	Hantzsch ester	–	–
10	CH_3_CN	HCOONa	–	8
11	DCE	DABCO, TBAI	additive (1.1 equiv)	–
12	DCE	AcOH, TFA, TFE	–	20
13	DCE	HFIP	–	24
14	DCE	HFIP	styrene (2 equiv)	24
15	DCE	HFIP	Ts-ABZ (1.5 equiv)	29

^a^NMR yield determined using CH_2_Br_2_ as internal standard, yield in parenthesis correspond to the isolated yield. ^b^Reaction was heated to 80 °C without light irradiation. ^c^Reaction carried out using blue light (440 nm).

Styrene was initially selected as model substrate since the addition of azide radicals generated by ABX was well reported [24,29]. We wanted to explore different classes of alkenes as the double bond substitution would greatly impact both the azide radical addition and the reactivity of the C-centered radical with Ph-EBX (**2**). Aliphatic alkenes, enamides, enol ethers and acrylates were tested in the reaction but did not lead to formation of the desired products (Scheme S1, Supporting Information File 1). In almost all cases >70% of the EBX reagent was left after 16 hours of reaction.

### Radical-polar crossover

Due to the disappointing results obtained with EBX reagents as SOMOphilic alkynes, we turned our attention to the development of a radical-polar crossover approach using photoredox catalysis. The final results obtained were described in our previous publication [45], but only the raw data for optimization was given in the form of tables in the Supporting Information. We now provide full insights into the different steps of the optimization process, highlighting the decisions taken and the unexpected results obtained.

Ts-ABZ (**3**) was kept as the azide radical source since it is known to be reduced by photocatalysts such as Cu(dap)_2_Cl [17]. This perfectly fits a catalytic cycle involving the reduction of Ts-ABZ (**3**) followed by oxidation of the carbon radical to form a carbocation and regenerate the ground state catalyst. Styrene (**1a**) was used as model substrate since the formation of a stabilized carbocation might be required for the reaction to occur. Xu [41] and Molander [42] previously reported the quenching of similar cationic species by alkynyl-BF_3_K salts. Boronate **5a** was therefore selected as nucleophilic alkyne. Gratifyingly, using Cu(dap)_2_Cl in DCE under blue light irradiation afforded **4a** in 17% NMR yield (Table 2, entry 1). The major byproduct formed during the transformation was identified as diazide **6**. When a copper photocatalyst is involved, a lot of diazidation can be observed. We assumed it could be caused by the reaction of Ts-ABZ (**3**) with a non-complexed copper catalyst formed during the transformation [24,51]. When iridium-based photocatalysts were tested, no product formation or only traces were observed (Table 2, entries 2 and 3). Using Ru(bpy)_3_Cl_2_·6H_2_O afforded 17% of **4a**, a similar yield as with Cu(dap)_2_Cl with a reduced formation of **6** (Table 2, entry 4). In contrast, Ru(bpz)_3_(PF_6_)_2_ did not form the desired product probably due to its too high reduction potential compared to Ts-ABZ (**3**) (value reported for ABX: *E*_1/2_(ABX) = −0.43 V vs SCE) [52] (Table 2, entry 5). In general, organic dyes could not catalyze the transformation except for 4ClDPAIPN which afforded 10% of yield of **4a** (Table 2, entries 6–10).

**Table 2:** Photocatalyst screening.^a^

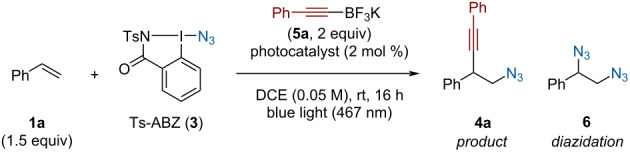				

Entry	Photocatalyst	*E*_1/2_ [PC^•+^/PC*] (V vs SCE)	Yield **4a** (%)^b^	Yield **6** (%)^b^

1	Cu(dap)_2_Cl	−1.43^c^	17	14
2	Ir(ppy)_3_	−1.88^c^	–	7
3	[Ir(ppy)_2_(dtbbpy)]PF_6_	−0.96^c^	<5	7
**4**	**Ru(bpy)** **_3_** **Cl** **_2_** **·6H** **_2_** **O**	**−0.81** **^c^**	**17**	**6**
5	Ru(bpz)_3_(PF_6_)_2_	−0.26^c^	–	<5
6	4*t-*BuCzIPN	−1.31^c^	–	<5
7	4DPAIPN	−1.52^d^	–	6
8	4ClDPAIPN	−1.30^d^	10	8
9	DPZ	−1.17^c^	<5	6
10	rose bengal	−0.68^c^	–	<5

^a^The data were already published in the supporting information of ref. [45] except for the yield of **6**. ^b^NMR yield determined using CH_2_Br_2_ as internal standard. ^c^Value taken from reference [53]. ^d^Value taken from reference [54].

No correlation between the different redox potentials of the photocatalysts and the yield of the reaction could be established. Ru(bpy)_3_Cl_2_·6H_2_O was selected as the optimal catalyst since it afforded the highest yield and minimized diazide formation.

Next, a solvent screening was performed as it can vastly influence reaction proceeding via a carbocation intermediate. Alkynyltrifluoroborates have a low solubility in chlorinated solvents but are well soluble in acetonitrile. Although this solvent has been used in similar transformation before [41–42,44], in our case only 9% of the desired product was obtained (Table 3, entry 2). A large quantity of product resulting from a Ritter-type reaction between acetonitrile and the carbocation intermediate could be observed by NMR [55]. Other highly polar solvents such as DMF and DMSO did not afford the homopropargylic azide (Table 3, entry 3). While alkynyl-BF_3_K salts are usually well soluble in acetone, carrying the reaction in this solvent only afforded traces of the product (Table 3, entry 4). Ethyl acetate or ether solvents such as dioxane and THF led to similar or slightly reduced yields (Table 3, entry 5). We were pleased to see that running the reaction in DME afforded 36% of **4a** (Table 3, entry 6). A mixture of DME with HFIP, known to stabilize carbocationic intermediates [56], slightly increased the yield (Table 3, entry 7).

**Table 3:** Solvent screening.^a^

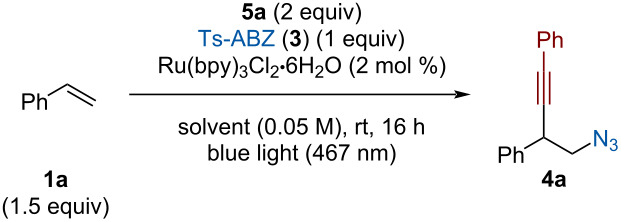		

Entry	Solvent	Yield **4a** (%)^b^

1	DCE	17
2	CH_3_CN	9
3	DMF, DMSO	–
4	acetone	<5
5	EtOAc, dioxane, THF	8–15
**6**	**DME**	**36**
7	DME/HFIP (9:1)	39

^a^The data were already published in the supporting information of ref. [45]. ^b^NMR yield determined using CH_2_Br_2_ as internal standard.

DME was selected for further optimization as the increased yield with the addition of HFIP was not significant enough to compensate the downside of having an expensive co-solvent. Next, the stoichiometry of the different reaction components was examined. When styrene (**1a**) was used as limiting reagent instead of Ts-ABZ (**3**), a slightly higher yield was observed (Table 4, entries 1 and 2). Increasing the excess of Ts-ABZ (**3**) from 1.25 to 1.5 equivalents had no impact on the reaction (Table 4, entry 3). Reducing the equivalents of **5a** to 1.25 only slightly diminished the yield while using 3 equivalents increased it to 44% (Table 4, entries 4 and 5). Surprisingly, **5a** could be used as limiting reagent without impacting the reaction (Table 4, entry 6). Carrying out the azido-alkynylation at low or high photocatalyst loading had no impact (Table 4, entry 7).

**Table 4:** Equivalent screening.^a^

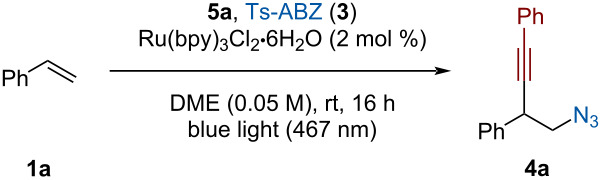		

Entry	Ts-ABZ/**1a**/**5a** (equiv)	Yield **4a** (%)^b^

1	1/1.5/2	36
**2**	**1.25/1/2**	**42**
3	1.5/1/2	41
4	1.25/1/1.25	39
5	1.25/1/3	44
6	1.5/1.5/1	43
7^c^	1.25/1/2	39–40

^a^The data were already published in the supporting information of ref. [45]. ^b^NMR yield determined using CH_2_Br_2_ as internal standard. ^c^1 or 5 mol % of photocatalyst were used.

Considering the robustness of the reaction to fluctuation in stoichiometry, conditions using styrene (**1a**) as limiting reagent, slight excess of Ts-ABZ (**3**) and 2 equivalents of **5a** were selected, while keeping in mind that further fine-tuning can be done (Table 4, entry 2). They offered the best compromise of yield, waste of materials and reproducibility at this scale. So far, the reactions were carried out for 16 hours as it is typical for photocatalyzed reaction to be slow. We realized that full conversion of the alkene was reached very rapidly. In fact, the reaction could be carried out for 1.5 h with no difference in yield (Table 5, entries 1 and 2). It is only below this reaction time that a difference was observed, 21% of product was still formed after 10 min of reaction (Table 5, entries 3 and 4).

**Table 5:** Reaction time, light source and concentration screening.^a^

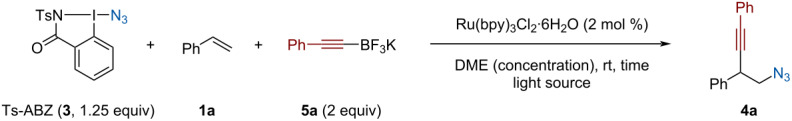				

Entry	Time (h)	Light source	*c* (M)	Yield **4a** (%)^b^

1	16	blue LEDs 467 nm (40 W)	0.05	42
**2**	**1.5**	**blue LEDs 467 nm (40 W)**	**0.05**	**42**
3	1	blue LEDs 467 nm (40 W)	0.05	39
4	0.17	blue LEDs 467 nm (40 W)	0.05	21
5	1.5	Kessil 467 nm (44 W)	0.05	42
**6**	**1.5**	**Kessil 467 nm (22 W)**	**0.05**	**42**
7	1.5	Kessil 440 nm (45 W)	0.05	41
8	1.5	green LEDs 525 nm (40 W)	0.05	35
9	4	CFL (8 W)	0.05	38
10	1.5	blue LEDs 467 nm (40 W)	0.025	36
11	1.5	blue LEDs 467 nm (40 W)	0.1-0.2	41

^a^Blue/green LEDs refers to LED strips attached to a crystallization flask. The data were already published in the supporting information of ref. [45]. ^b^NMR yield determined using CH_2_Br_2_ as internal standard.

We then turned our attention to the light source used to irradiate the reaction. Initially, a homemade set-up using blue LED strips was used. When it was replaced by a commercially available Kessil lamp of the same wavelength and intensity we observed a similar yield (Table 5, entry 5). Reducing the strength of the irradiation from 44 to 22 W had no impact, similarly using a more energetic wavelength afforded the same yield (Table 5, entries 6 and 7). When lower energy light source such as green LEDs or a compact fluorescent lamp (CFL) were used only a small decrease in yield could be observed, although 4 hours of irradiation were needed to reach full conversion using CFL (Table 5, entries 8 and 9). The concentration, a factor expected to play an important role in a three-component reaction, had surprisingly little influence on the transformation. At lower concentration only a slight decrease of yield was observed, whereas higher concentration led to a similar yield (Table 5, entries 10 and 11).

**Scheme 2:**
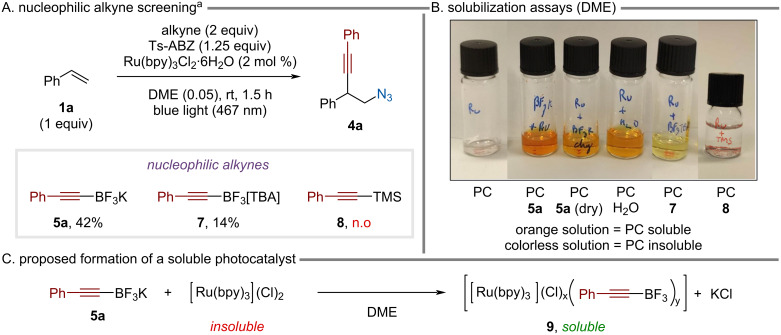
Screening of nucleophilic alkynes and investigation of the photocatalyst solubility. n.o = not observed, PC = photocatalyst. ^a^The data were already published in the supporting information of ref. [45].

The source of nucleophilic alkyne was evaluated, changing the counter ion from potassium to tetrabutylammonium (**7**) reduced the yield to 14% (Scheme 2A). When TMS-alkyne **8** was used, no product formation occurred. In this case, we observed that the initial reaction mixture before light irradiation was colorless. This was surprising, as in all the previous experiments a yellow/orange mixture was obtained due to the presence of the photocatalyst. Further investigation revealed that Ru(bpy)_3_Cl_2_·6H_2_O is not soluble in DME (Scheme 2B). In contrast, when it is in the presence of alkynyl-BF_3_K it readily dissolves. The addition of a couple of water drops to a suspension of photocatalyst in DME also allowed solubilization. The solubilization due to residual water coming from the alkynyl-BF_3_K was ruled out by careful drying of **5a**. We postulated that an ion exchange between Ru(bpy)_3_Cl_2_ and **5a** can occur to afford a more soluble photocatalyst with the general formula **9** (Scheme 2C). Interestingly, when the solubilization experiment was carried out with the tetrabutylammonium salt **7** only moderate solubilization occurred (Scheme 2B), which could explain the lower yield previously observed (Scheme 2A). Neutral TMS-alkyne **8** cannot be involved in ion exchange and therefore this could be one of the reasons why no reaction occurred.

Next, we turned our attention to the temperature of the reaction, a factor rarely explored in photocatalyzed reaction due to the lack of available set-ups allowing an efficient light irradiation in addition to a proper temperature control. In our case, since the reaction previously showed to be very tolerant to different light intensity we could attempt to cool the reaction. Carrying out the transformation with the reaction vessel placed in an EtOAc bath cooled to 0 °C by an immersion cooler allowed to increase the yield to 50% (Table 6, entries 1 and 2). Decreasing the temperature to −20 °C slightly improved the yield and the mass balance of the reaction (Table 6, entry 3). At −41 °C the reaction time had to be increased to reach full conversion of styrene (**1a**), and the yield slightly decreased (Table 6, entry 4). As full conversion is still reached rapidly at −20 °C, we were interested to use a more convenient cooling bath made from ice and salt rather than the immersion cooler. We were pleased to see that running the reaction with this method of cooling afforded similar yield (Table 6, entry 5) even with a significantly more opaque bath mixture.

**Table 6:** Temperature screening.^a^

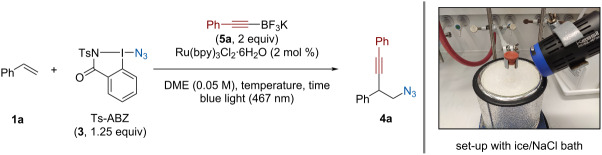				

Entry	*T* (°C)	Method of cooling	Yield **4a** (%)^b^	Mass balance (%)^c^

1	rt	–	42	54
2	0	immersion cooler	50	64
3	−20	immersion cooler	52	71
4^d^	−41	immersion cooler	46	71
**5**	**−20**	**ice and NaCl bath**	**53**	**69**

^a^In all experiment the Kessil lamp was pointed diagonally towards the reaction vessel immersed in a cooling bath contained in a Dewar. An immersion cooler was placed in an EtOAc bath. The data were already published in the supporting information of ref. [45] except for the mass balance. The photo is a cropped version taken from ref. [45]. ^b^NMR yield determined using CH_2_Br_2_ as internal standard. ^c^The mass balance corresponds to the quantity of product + byproducts that could be determined by NMR integration. ^d^Reaction was carried out for 3 h.

Having already modified most of the reaction parameters available we decided to explore the effect of different additives. In most reactions, **10** resulting from water addition was observed to some extent (4–8%), the presence of 4 Å molecular sieves effectively suppressed the formation of **10** (Table 7, entries 1 and 2). Unfortunately, the yield did not increase and only a slightly worse mass balance was observed. Multiple reagents are known to abstract fluorine from trifluoroborate salts to form the corresponding difluoroborate species [57–60]. This approach has previously been successful to increase the yield of different transformations. Using fluoride scavenger such as TMSCl, TFAA or TMS_2_(O) led to similar or lower yields (Table 7, entries 3–5). We were pleased to see that in the presence of BF_3_·Et_2_O, **4a** was obtained in 75% yield (Table 7, entry 6). Addition of a less acidic boron Lewis acid B(OTFE)_3_ had a detrimental effect (Table 7, entry 7). Increasing the stoichiometry of BF_3_ to 2 equivalents lowered the yield significantly (Table 7, entry 8). In contrast, the loading could be reduced to 30 mol % without impacting the formation of **4a** (Table 7, entries 9 and 10).

**Table 7:** Additive screening.

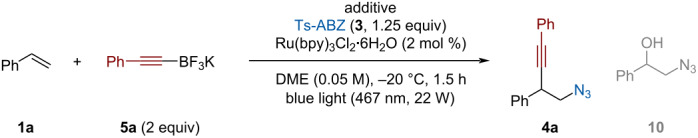				

Entry	Additive	Equivalent	Yield **4a** (%)^b^	Yield **10** (%)^b^

1	–	–	53	8
2	MS 4 Å	50 mg/0.1 mmol	51	<5
3	TMSCl	1 equiv	40	–
4	TFAA	1 equiv	54	–
5	(TMS)_2_O	1 equiv	48	13
6	BF_3_·Et_2_O	1 equiv	75	–
7	B(OTFE)_3_	1 equiv	53	10
8	BF_3_·Et_2_O	2 equiv	37	n.d.
**9**	**BF** **_3_** **·Et** **_2_** **O**	**0.3 equiv**	**72**	**9**
10	BF_3_·Et_2_O	0.1 equiv	67	13

^a^The data were already published in the supporting information of Ref. [45] except for the yield of **10** and for entry 10. ^b^NMR yield determined using CH_2_Br_2_ as internal standard. n.d. = not determined.

Finally, a fine-tuning of the reaction conditions was performed. Increasing the concentration to 0.1 M afforded a comparable yield (Table 8, entries 1 and 2). Similarly, the equivalents of alkynyl-BF_3_K **5a** could be reduced to 1.5, a further decrease started to impact the yield (Table 8, entries 3 and 4). Carrying out the reaction in degassed solvent is not mandatory but affords a better result (Table 8, entry 5). The photocatalyst could be changed to Ru(bpy)_3_(PF_6_)_2_ without impacting the reaction (Table 8, entry 6). This photocatalyst was ultimately chosen since we observed a sharp decrease in yield when Ru(bpy)_3_Cl_2_·6H_2_O was used with alkyl-substituted alkynyl-BF_3_K, probably due to the poor solubilization of the catalyst. Finally, control experiments in the absence of photocatalyst or light only afforded traces of product (Table 8, entry 7).

**Table 8:** Fine-tuning of the reaction conditions.

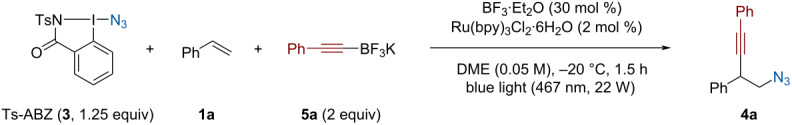		

Entry	Change from standard conditions	Yield **4a** (%)^b^

1	none	72
2	*c* = 0.1 M	75
3	*c* = 0.1 M, **5a** (1.5 equiv)	74
4	*c* = 0.1 M, **5a** (1.25 equiv)	71
5	*c* = 0.1 M, **5a** (1.5 equiv), no degassing	65
**6**	***c*** ** = 0.1 M, 5a (1.5 equiv), Ru(bpy)** **_3_** **(PF** **_6_** **)** **_2_** ** (2 mol %)**	**74**
7	no photocatalyst or light, 16 h	<5

^a^The data were already published in the supporting information of ref. [45]. ^b^NMR yield determined using CH_2_Br_2_ as internal standard.

**Scheme 3:**
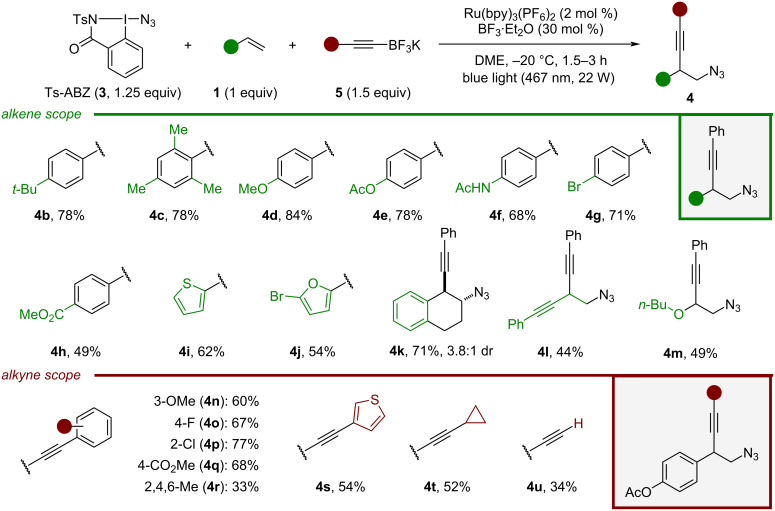
Selected scope entries of the azido-alkynylation. The data were already published in ref. [45].

The successful scope of the reaction was already described in our previous work [45], and will be only shortly described in a summarized form (Scheme 3). Styrene substituted with a *tert*-butyl at the *para* position afforded **4b** in 78% yield. Using a styrene bearing a sterically hindered aryl afforded **4c** in a similar yield. Homopropargylic azides possessing oxygen substituents such as **4d** and **4e** could be obtained in 84% and 78% yield, respectively. We were pleased to see that the presence of an acetamide did not shut down the reaction, the corresponding product **4f** was obtained in 68%. The amide could have competed with the alkynyl-trifluoroborate for the carbocation trapping. An halogen-substituted styrene could be azido-alkynylated to afford **4g** in 71% yield. Electron-poor aryls are expected to destabilize the carbocation intermediate formed during the reaction. Nevertheless, **4h** possessing a *para* ester could be obtained, albeit with a decreased yield. Homopropargylic azides substituted with heteroaryls such as thiophene (**4i**) or a bromofuran (**4j**) were obtained in 62% and 54% yield, respectively. When the azido-alkynylation was carried out on 1,2-dihydronaphthalene, **4k** could be obtained in 71% yield and 3.8:1 dr. As expected, the configuration of the major diasteroisomer was determined to be *trans*. Diyne **4l** could be accessed in 44% yield from the exclusive 1,2-functionalization of the corresponding ene-yne. Crude NMR of the reaction did not show the presence of an allene product which could have been formed by a 1,4-functionalization. Enol ether could also be involved in the reaction, affording **4m** in 49% yield.

Next, we explored the variety of nucleophilic alkynes that could be introduced using *p*-acetoxystyrene as model substrate. Alkynyl-trifluoroborates bearing an electron-donating group (OMe) or electron-withdrawing groups (F, Cl, CO_2_Me) on the aryl ring worked well in the transformation, affording homopropargylic azides **4n–q** in 60–77% yield. The reaction appears to be sensitive to the steric hindrance of the nucleophile: addition of a mesitylalkyne only formed 33% of **4r**. Pleasingly, heteroaryl substituents were tolerated, **4s** bearing a thiophene was obtained in 54% yield. The reaction could be extended to alkyl-substituted alkynes with little difference in yields. An homopropargylic azides possessing a cyclopropyl (**4t**) was formed in 52% yield. Finally, starting from potassium ethynyltrifluoroborate the free alkyne **4u** could be accessed in 34% yield with potential for further modification by cross-coupling. The full scope of the transformation can be found in Supporting Information File 1, Schemes S2 and S3 [45].

**Scheme 4:**
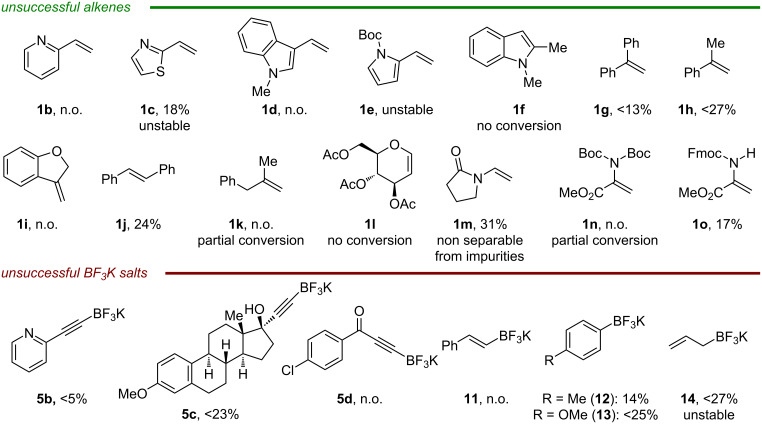
Unsuccessful examples. The conditions used are the same as in Scheme 3. The yields reported were determined by NMR on crude mixtures. n.o. = not observed.

Concerning scope limitations, the azido-alkynylation of vinyl-pyridine **1b** was unsuccessful and thiazole **1c** only afforded 18% of the desired product, which proved to be unstable during purification (Scheme 4). Homopropargylic azides containing electron-rich nitrogen heterocycles could not be obtained. No product was observed by using vinylindole **1d** probably due its high tendency to polymerize. Although a slight amount of homopropargylic azide was formed using pyrrole **1e** it could not be isolated due to its instability. No conversion was observed when the transformation was attempted directly on the indole scaffold **1f**.

Only a small amount of product was formed using styrene **1g** bearing an extra α-phenyl substituent. A slightly higher yield was observed when α-methylstyrene (**1h**) was used instead but it remained low (<27%). In general, when the reaction performed poorly (<30%) a large number of byproducts are formed, making the separation of the desired compound from the impurities impossible. Attempting the reaction on the cyclic substrate **1i** did not afford the desired product. Although β-substitution on the alkene is tolerated, using *trans*-stilbene only afforded 24% yield. Unfortunately, aliphatic alkenes are not tolerated in the reaction. When **1k** was used no product was formed and only partial conversion was observed. As a simple enol ether was reactive in the transformation, we attempted to use glucal **1l** but no conversion was observed. Enamide **1m** could be azido-alkynylated in low yield and the product was inseparable from impurities. When dehydroalanine **1n** was used in the reaction no product was formed and only partial conversion of the starting material was observed. We postulated that the presence of two Boc protecting groups on the nitrogen makes the oxidation of the C-centered radical challenging. By using **1o** only bearing one protecting group the desired product could be obtained, albeit in only 17% yield.

Pyridine-substituted alkynyl-BF_3_K **5b** could not be transferred, and only traces of the product were observed. When **5c** derived from mestranol was involved in the reaction, the corresponding homopropargylic azide was obtained in <23% yield. We noticed that this particular alkynyltrifluoroborate salt has a low solubility in DME, which might cause the low yield observed. Ynonetrifluoroborate **5d** could not be introduced as nucleophile, probably due to its reduced reactivity caused by the electron-withdrawing acyl group. Unfortunately, although Molander previously reported the use of vinyl-trifluoroborate **11** to trap benzylic carbocation [42], in our case no product was observed. Since β-substituted styrenes can be involved in the transformation it is possible that the azide radicals can react either with the vinylic product formed in the reaction or with the starting vinyl-BF_3_K. Simple aryl-BF_3_K **12** only afforded 14% yield of the azido-arylated product, and using the more nucleophilic **13** only slightly improved the yield. Finally, a small amount of product resulting from the addition of allyl-BF_3_K **14** could be observed but decomposed during purification.

**Scheme 5:**
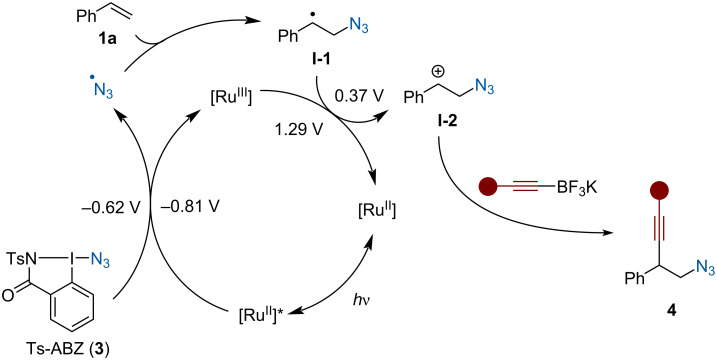
Proposed mechanism.

Based on literature precedence [41–42,44] and experimental studies [45] a plausible photocatalytic cycle could be proposed (Scheme 5). Upon light irradiation, single-electron reduction of Ts-ABZ (**3**) (*E*_1/2_^red^ = −0.62 V vs SCE) [45] by the excited state photocatalyst (*E*_1/2_ [Ru^III^/Ru^II^*] = −0.86 V vs SCE) [53] can occur to form an azide radical, which upon addition to styrene would generate intermediate **I-1**. Oxidation of the benzylic radical (*E*_1/2_^ox^ = +0.37 V vs SCE for the corresponding radical without the azide) [61] by the previously formed Ru(bpy)^3+^ (*E*_1/2_ [Ru^III^/Ru^II^] = +1.29 V vs SCE) [53] regenerates the ground state photocatalyst and forms carbocation **I-2**. Finally, nucleophilic addition of the alkynyl-trifluoroborate onto the benzylic carbocation would afford homopropargylic azide **4** [45].

## Conclusion

In summary, an azido-alkynylation of styrenes to access homopropargylic azides was developed. The reaction was initially investigated using EBX reagents as SOMOphilic alkynes but this only afforded a 29% yield of the desired product. After switching to a radical-polar crossover approach the yield could be significantly improved. Key to the reaction optimization was the reduction of the temperature to −20 °C and the addition of BF_3_·Et_2_O. A large variety of homopropargylic azides could be obtained in 30–84% yield using Ts-ABZ (**3**) as azide radical precursor and alkynyltrifluoroborates as nucleophilic alkyne source [62].

## Supporting Information

File 1 General methods, photochemistry set-up, reaction optimization, experimental procedures and compounds characterization.

## Data Availability

All data that supports the findings of this study is available in the published article and/or the supporting information to this article.
