# Supplementary material for: SOMOphilic alkyne vs radical-polar crossover approaches: The full story of the azido-alkynylation of alkenes
Source: Beilstein J Org Chem. 2024 Apr 3;20:701–13. doi: 10.3762/bjoc.20.64 (PMC10999984; doi:10.3762/bjoc.20.64)
Supplement: File 1 — General methods, photochemistry set-up, reaction optimization, experimental procedures and compounds characterization. [file Beilstein_J_Org_Chem-20-701-s001.pdf]

BEILSTEIN JOURNAL OF ORGANIC CHEMISTRY

Supporting Information

for

SOMOphilic alkyne vs radical-polar crossover approaches: The full story of the azido-alkynylation of alkenes

Julien Borrel and Jerome Waser

Beilstein J. Org. Chem. **2024,** *20,* 701–713. doi:10.3762/bjoc.20.64

General methods, photochemistry set-up, reaction optimization, experimental procedures and compounds characterization

License and Terms: This is a supporting information file under the terms of the Creative Commons Attribution License (https://creativecommons.org/licenses/by/4.0). Please note that the reuse, redistribution and reproduction in particular requires that the author(s) and source are credited and that individual graphics may be subject to special legal provisions.
The license is subject to the *Beilstein Journal of Organic Chemistry* terms and conditions: (https://www.beilstein-journals.org/bjoc/terms)

Table of Contents

1. General methods S2

2. Picture of the photochemistry set-up S4

3. Additional schemes S5

4. Reaction optimization S6

5. Synthesis of alkenes S6

6. Synthesis of potassium trifluoroborate salts S9

7. Annexes S11

   7.1 Synthesis of HIRs previously reported by our group S11

   7.2 Synthesis of alkenes previously reported by our group S14

   7.3 Synthesis of potassium trifluoroborate salts previously reported by our group S20

   7.4 Scope of the azido-alkynylation previously reported by our group S29

1. General methods

All reactions were carried out under air unless stated otherwise. Reactions requiring heating were carried out using DrySyn heating block. For flash chromatography, distilled technical grade solvents were used. THF, toluene, Et_2O and CH_2Cl_2 were dried by passage over activated alumina under nitrogen atmosphere (H_2O content <10 ppm, Karl-Fischer titration). Solvents were degassed by bubbling with a balloon of argon. All chemicals were purchased from Acros, Aldrich, Combi-blocks, Fluka, Fluorochem, Merck, TCI or VWR and used as such unless stated otherwise.

Chromatographic purification was performed as flash chromatography using Silicycle silica 40–63 µm (230–400 mesh), using the solvents indicated as eluent with 0.1–0.5 bar pressure or using Biotage Isolera Spektra One with pre-packaged silica cartridges purchased from Buchi, models: Sepacore or GraceResolve (4 g, 12 g, 25 g, 40 g, 80 g, 120 g). When indicated purification were performed on activated neutral aluminium oxide (Brockmann activity I). TLC was performed on Merck silica gel 60 F_{254} TLC glass plates and visualized with UV light and potassium permanganate or *p*-anisaldehyde stain.

^1H NMR spectra were recorded on a Bruker DPX-400 400 MHz spectrometer in chloroform-d, DMSO-d_6 or acetone-d_6. All signals are reported in ppm using the residual solvent signal as internal reference (chloroform-*d*: 7.26 ppm, DMSO-d_6: 2.50 ppm, acetone-d_6: 2.06 ppm). The data is being reported as (s = singlet, d = doublet, t = triplet, q = quadruplet, qi = quintet, m = multiplet or unresolved, bs = broad signal, coupling constant(s) in Hz, integration, assignment). ^{13}C NMR spectra were recorded with {^1H} decoupling on a Bruker DPX-400 101 MHz spectrometer in chloroform-*d*, DMSO-d_6 or acetone-d_6. All signals are reported in ppm using the residual solvent signal as internal reference (chloroform-*d*: 77.16 ppm, DMSO-d_6: 39.52 ppm, acetone-d_6: 206.26 and 29.84 ppm). ^{19}F NMR spectra were recorded with {^1H} decoupling on a Bruker DPX-400 376 MHz spectrometer in chloroform-*d*, DMSO-d_6 or acetone-d_6. ^{11}B NMR spectra were recorded on a Bruker DPX-400 128 MHz spectrometer in DMSO-d_6 or acetone-d_6.

High-resolution mass spectrometric measurements were performed by the mass spectrometry service of ISIC at the EPFL. Electrospray-ionisation HRMS data were acquired on a Q-Tof Ultima mass spectrometer (Waters) or a Q-Tof 6530 Accurate mass spectrometer (Agilent) operated in the positive ionization mode and fitted with a standard Z-spray ion source equipped with the Lock-Spray interface. Data from the Lock-Spray were used to calculate a correction factor for the mass scale and provide accurate mass information of the analyte. Data were processed using the MassLynx 4.1 software. Atmospheric pressure photo-ionisation (APPI) HRMS measurements were done on a LTQOrbitrap Elite instrument (Thermofisher) operated in the positive ionization mode.

Reactions under “blue LEDs irradiation” (440 nm, 40 W) were performed in test tubes (14 mL, soda-lime glass, wall thickness = 0.8 mm) which were placed at the center of a crystallization flask. On this flask were attached the blue LEDs (RUBAN LED 5MÈTRES - 60LED/M -3528 BLEU - IP65 with Transformateur pour Ruban LED 24W/2A/12V, bought directly on RubanLED.com). The distance between the LEDs and the test tubes was approximatively 3

cm. Long irradiation resulted in temperature increasing up to 35 °C during overnight reactions. Reactions using “Kessil lamp” of 467 nm or 440 nm used the corresponding models PR160L-467 or PRL160L-440.

2. Picture of the photochemistry set-up

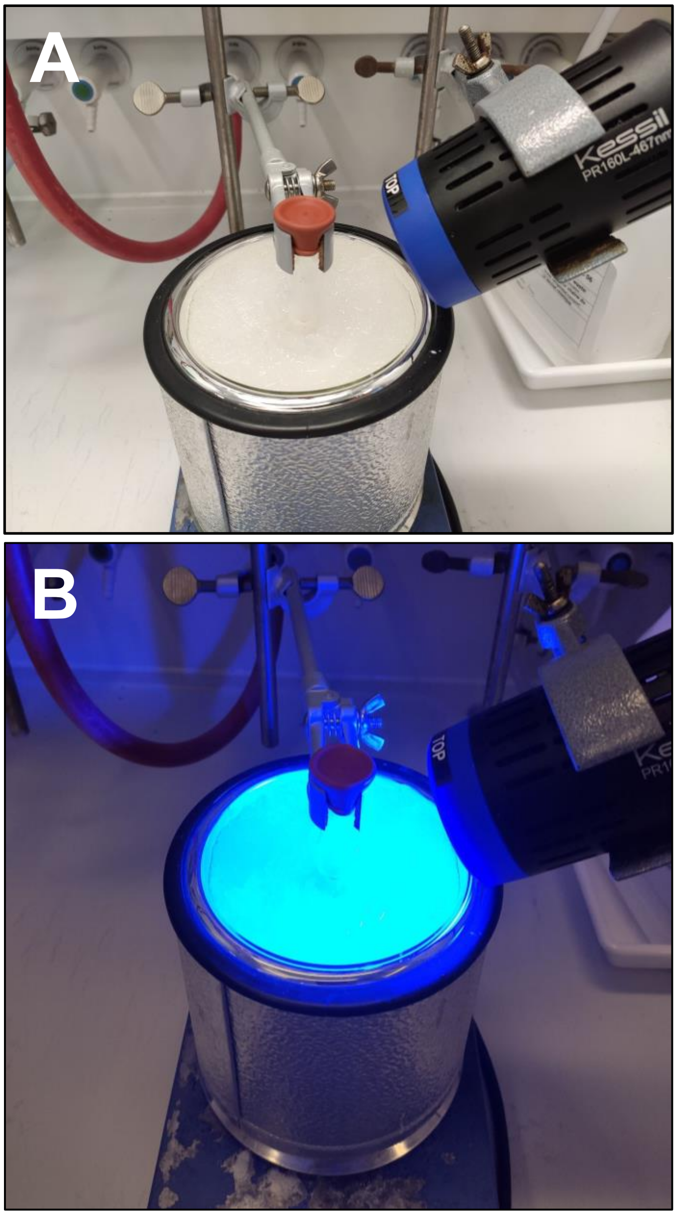

Figure S1: A) Picture of the set-up before turning on the Kessil lamp. B) Picture of the set-up during the reaction. The photos were taken from Ref. 13.

The Kessil lamp was placed diagonally at a distance of ≈4 cm from the top of the reaction vessel (test tube or round-bottom flask). The latter was immersed in a bath of ice and salt (–20 °C) contained in a Dewar. For the optimized conditions the lamp intensity was set at 50% (22 W).

3. Additional schemes

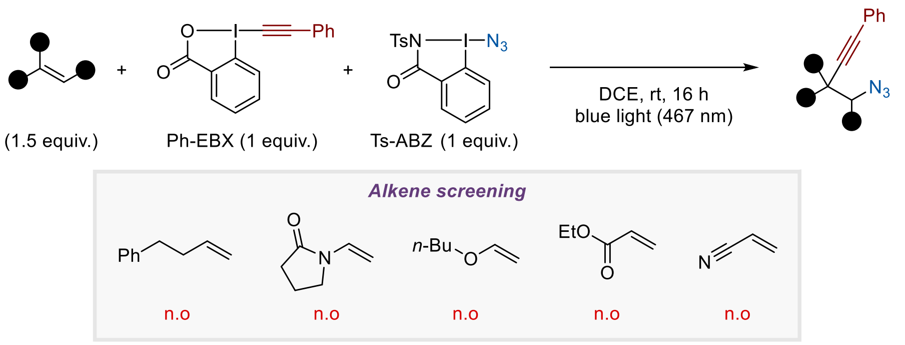

Scheme S1: Alkenes screening for the SOMOphilic alkyne approach.

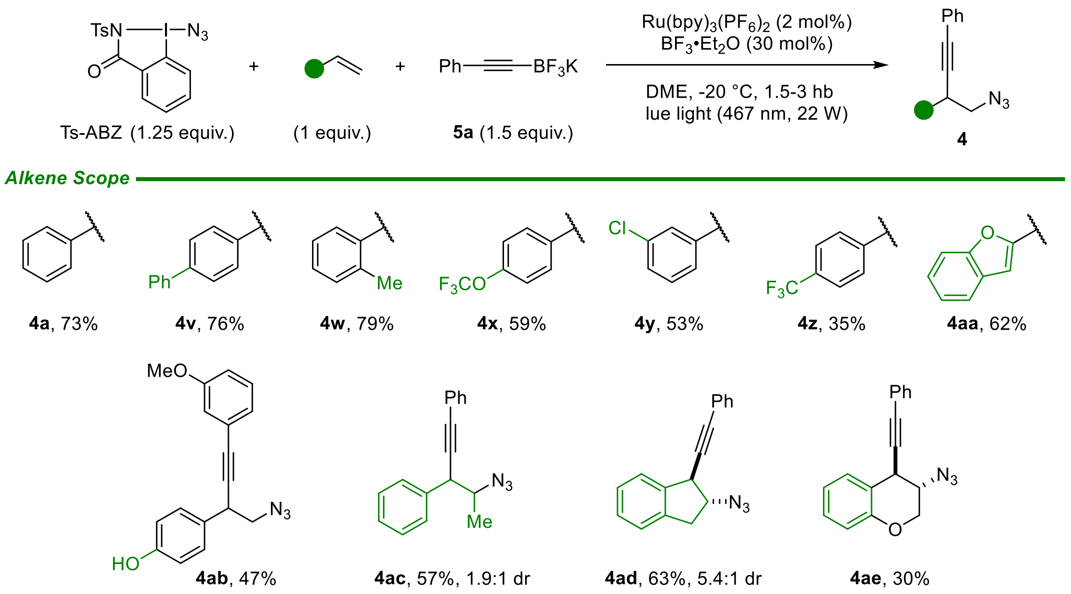

Scheme S2: Additional alkene scope.

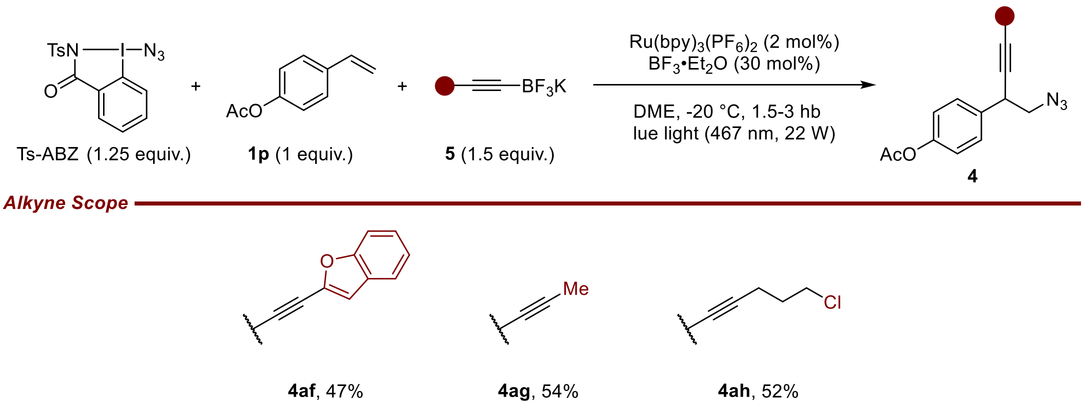

Scheme S3: Additional alkyne scope.

4. Reaction optimization

Optimization protocol for the SOMOphilic approach (0.1 mmol scale):

An oven-dried test tube charged with the solid reagents was evacuated and backfilled with N_2 (3×). Dry degassed solvent and the liquid reagents and additives were added. The reaction was stirred under blue light irradiation then concentrated in vacuo. ^1H NMR yield was determined by dissolving crude **4a** in $CDCl_3$ and adding CH_2Br_2 (3.5 µL, 0.049 mmol, 0.49 equiv) as internal standard. The signal at 4.13 ppm was used to determine the yield.

Optimization protocol for the radical-polar crossover approach (0.1 mmol scale):

An oven-dried test tube charged with the solid reagents was evacuated and backfilled with N_2 (3×). Dry degassed solvent and the liquid reagents were added and the mixture was cooled to the desired temperature (if relevant). Then, additive (if relevant) was added and the reaction was stirred under light irradiation. The reaction mixture was filtered through a short plug of silica and eluted with DCM then concentrated in vacuo. ^1H NMR yield was determined by dissolving crude **4a** in $CDCl_3$ and adding CH_2Br_2 (3.5 µL, 0.049 mmol, 0.49 equiv) as internal standard. The signal at 4.13 ppm was used to determine the yield.

5. Synthesis of Alkenes

General procedure A:

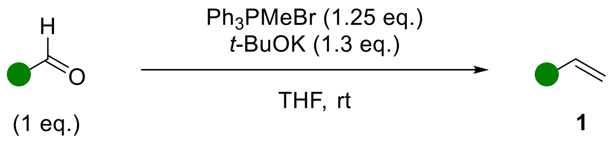

Following a reported procedure,[1] an oven dried round-bottom flask charged with methyltriphenylphosphonium bromide (1.79 g, 5.00 mmol, 1.25 equiv) and potassium *tert*-butoxide (584 mg, 5.20 mmol, 1.30 equiv) was evacuated and backfilled with N_2. Dry THF (11 mL) was added and the mixture was stirred at rt for 30 min. A solution of aldehyde (4.00 mmol, 1 equiv) in dry THF (5 mL) was added dropwise over 5 min and the reaction was stirred at rt under N_2 until full conversion was observed by TLC. The reaction was quenched with 35 mL of a sat. sol. of NH_4Cl and the mixture was extracted with 3 × 40 mL of Et_2O or EtOAc. The combined organic layers were washed with brine, dried over $MgSO_4$, filtered and concentrated in vacuo. The crude product was purified by column chromatography to obtain alkene **1**.

2-Vinylthiazole (1c):

[1] R. J. Maza, E. Davenport, N. Miralles, J. J. Carbó, E. Fernández, *Org. Lett.* **2019**, *21*, 2251–2255.

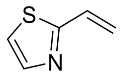

1c

Synthesized following general procedure **A** starting from thiazole-2-carbaldehyde (0.35 mL, 4.0 mmol). The reaction was carried out for 1 h and extractions were performed with Et_2O. The crude product was loaded on celite and purified by column chromatography (pentane/Et_2O, 1:0 to 9:1) to afford 2-vinylthiazole (**1c**) (153 mg, 1.37 mmol, 34%) as a light-yellow oil.

^{1}H NMR (400 MHz, $CDCl_3$) δ 7.77 (d, *J* = 3.3 Hz, 1H, Ar*H*), 7.24 (d, *J* = 3.2 Hz, 1H, Ar*H*), 6.94 (dd, *J* = 17.5, 11.0 Hz, 1H, *H*C=CH_2), 6.05 (d, *J* = 17.5 Hz, 1H, HC=CH_2), 5.55 (d, *J* = 10.9 Hz, 1H, HC=CH_2). ^{13}C NMR (101 MHz, $CDCl_3$) δ 167.4, 143.5, 130.6, 119.9, 118.6. Spectroscopic data was consistent with the values reported in the literature.[2]

Note: Product was stored at −20 °C after isolation. It was used the next day in the azido-alkynylation reaction.

1-Methyl-3-vinyl-1*H*-indole (1d):

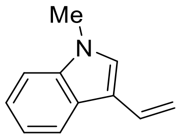

1d

Synthesized following general procedure **A** starting from 1-methyl-1*H*-indole-3-carbaldehyde (637 mg, 4.00 mmol). The reaction was carried out for 1 h and extractions were performed with EtOAc. The crude product was purified by column chromatography (pentane/Et_2O, 9:1) to afford 1-methyl-3-vinyl-1*H*-indole (**1d**) (559 mg, 3.56 mmol, 89%) as a colorless oil.

^{1}H NMR (400 MHz, $CDCl_3$) δ 7.88 (d, *J* = 7.9 Hz, 1H, Ar*H*), 7.34 – 7.23 (m, 2H, Ar*H*), 7.18 (td, *J* = 7.5, 1.2 Hz, 1H, Ar*H*), 7.12 (s, 1H, C=C*H*N), 6.88 (dd, *J* = 17.8, 11.3 Hz, 1H, *H*C=CH_2), 5.67 (dd, *J* = 17.8, 1.4 Hz, 1H, HC=CH_2), 5.14 (dd, *J* = 11.3, 1.4 Hz, 1H, HC=CH_2), 3.77 (s, 3H, CH_3). ^{13}C NMR (101 MHz, $CDCl_3$) δ 137.7, 129.4, 128.3, 126.3, 122.2, 120.3, 120.0, 114.4, 110.1, 109.6, 32.9. Spectroscopic data was consistent with the values reported in the literature.[3]

Note: Product was stored at −20 °C after isolation. It was used the next day in the azido-alkynylation reaction. We observed substantial degradation after 2–3 days of storage.

---

[2] T. Kobayashi, H. Yorimitsu, K. Oshima, *Chem. - Asian J.* **2011**, *6*, 669–673.

[3] T. Boi, A. Deagostino, C. Prandi, S. Tabasso, A. Toppino, P. Venturello, *Org. Biomol. Chem.* **2010**, *8*, 2020–2027.

tert-Butyl 2-vinyl-1_H_-pyrrole-1-carboxylate (1e):

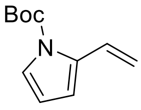

1e

Synthesized following general procedure **A** starting from _tert_-butyl 2-formyl-1_H_-pyrrole-1-carboxylate (781 mg, 4.00 mmol). The reaction was carried out for 2 h and extractions were performed with Et_2O. The crude product was loaded on celite and purified by column chromatography (pentane/Et_2O, 98:2) to afford _tert_-butyl 2-vinyl-1_H_-pyrrole-1-carboxylate (**1e**) (569 mg, 2.94 mmol, 74%) as a slightly yellow oil.

^{1}H NMR (400 MHz, $CDCl_3$) δ 7.26 – 7.18 (m, 2H, Ar_H_ + _H_C=CH_2), 6.44 – 6.39 (m, 1H, Ar_H_), 6.13 (t, _J_ = 3.3 Hz, 1H, Ar_H_), 5.52 (dd, _J_ = 17.6, 1.7 Hz, 1H, HC=CH_2), 5.11 (dd, _J_ = 11.1, 1.6 Hz, 1H, HC=CH_2), 1.60 (s, 9H, C(CH_3)$_3$). Spectroscopic data was consistent with the values reported in the literature.[4]

<u>Note:</u> Product was stored at −20 °C after isolation. It was used the next day in the azido-alkynylation reaction.

3-Methylene-2,3-dihydrobenzofuran (1i):

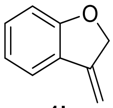

1i

Synthesized following general procedure **A** starting from benzofuran-3(2_H_)-one (537 mg, 4.00 mmol). The reaction was carried out for 3 h and extractions were performed with Et_2O. The crude product was purified by column chromatography (pentane) to afford 3-methylene-2,3-dihydrobenzofuran (**1i**) (292 mg, 2.21 mmol, 55%) as a colorless oil which solidify in the fridge.

^{1}H NMR (400 MHz, $CDCl_3$) δ 7.41 (dd, _J_ = 7.6, 1.0 Hz, 1H, Ar_H_), 7.23 – 7.17 (m, 1H, Ar_H_), 6.90 (td, _J_ = 7.5, 0.9 Hz, 1H, Ar_H_), 6.86 (d, _J_ = 8.1 Hz, 1H, Ar_H_), 5.40 (t, _J_ = 3.2 Hz, 1H, CH_2), 5.10 (t, _J_ = 3.0 Hz, 2H, C=CH_2), 4.99 (t, _J_ = 2.7 Hz, 1H, CH_2). ^{13}C NMR (101 MHz, $CDCl_3$) δ 163.9, 144.0, 130.6, 126.0, 121.0, 120.8, 110.8, 99.6, 75.1. Spectroscopic data was consistent with the values reported in the literature.[5]

[4] J. Waser, B. Gaspar, H. Nambu, E. M. Carreira, _J. Am. Chem. Soc._ **2006**, _128_, 11693–11712.
[5] S. Braune, M. Pohlman, U. Kazmaier, _J. Org. Chem._ **2004**, _69_, 468–474.

6. Synthesis of potassium trifluoroborate salts

General note: It is known that carbons linked to the boron atom are difficult to be observed by ^{13}C NMR due to a broadening of the signal caused by the quadrupole moment of ^{11}B nuclei. This implies that the two carbons of the alkyne (in alkynyl-BF_3K) are too broad to be properly visible.[6] Therefore, they are not listed in the characterization data.

General procedure B:

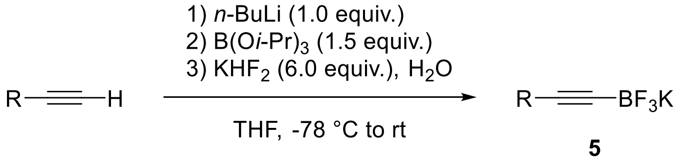

Following a reported procedure,[7,8] an oven-dried round-bottom flask (PFA), charged with alkyne (1.0 equiv) if solid, was evacuated and backfilled with N_2 (3×). Then, alkyne (if liquid) and dry THF (0.3 M) were added. The mixture was cooled to −78 °C and a solution of *n*-BuLi (2.5 M, 1.0 equiv) in hexane was added dropwise under N_2. The reaction was stirred at −78 °C for 1 h and B(O*i*-Pr)$_3$ (1.5 equiv) was added quickly. The reaction was stirred 10 min at −78 °C then 2 h at rt. The mixture was cooled to 0 °C and a saturated solution of KHF_2 (6.0 equiv) in water (40% of THF volume + additional 40% to rinse the remaining solid) was added. The reaction was stirred at rt open to air for 2 h then concentrated in vacuo. The wet solid obtained was further dried by co-evaporation with toluene (3×). To the dry solid was added acetone (≈50 mL) and the resulting mixture was placed on a rotary evaporator and rotated rapidly at atmospheric pressure with the bath set at 45 °C for 15 minutes. The flask was removed and the mixture carefully filtered taking care to leave the insoluble material (KHF_2) in the reaction flask. Acetone was once again added and the process (heating for 15 min then collection of the liquid) was repeated 2 more times. The combined acetone filtrates were concentrated in vacuo to approximately 1/3 of the initial volume. Et_2O (≈60 mL) was added causing a white solid to precipitate. The mixture was cooled to 0 °C for 10 min then filtered. The solid obtained was washed with Et_2O and dried in vacuo to afford the desired potassium alkynyl-trifluoroborate **5**.

Note: This purification procedure usually affords the pure desired product. If it is not the case a more classical recrystallization from acetone/Et_2O can be performed.

[6] R. A. Oliveira, R. O. Silva, G. A. Molander, P. H. Menezes, *Magn. Reson. Chem.* **2009**, *47*, 873–878.
[7] D. A. Mundal, K. E. Lutz, R. J. Thomson, *J. Am. Chem. Soc.* **2012**, *134*, 5782–5785.
[8] J. Borrel, J. Waser, *Org. Lett.* **2022**, *24*, 142–146.

Potassium trifluoro(pyridin-2-ylethynyl)borate (5b):

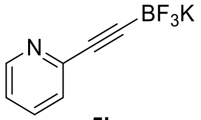

5b

Synthesized following general procedure **B** starting from 2-ethynylpyridine (0.80 mL, 7.5 mmol). Potassium trifluoro(pyridin-2-ylethynyl)borate (**5b**) (547 mg, 2.62 mmol, 35%) was obtained as a brown solid.

^{1}H NMR (400 MHz, acetone-d$_6$) δ 8.43 (ddd, *J* = 4.9, 1.8, 0.9 Hz, 1H, Ar*H*), 7.63 (td, *J* = 7.7, 1.9 Hz, 1H, Ar*H*), 7.30 (dt, *J* = 7.9, 1.1 Hz, 1H, Ar*H*), 7.16 (ddd, *J* = 7.6, 4.9, 1.2 Hz, 1H, Ar*H*). ^{13}C NMR (101 MHz, acetone-d$_6$) δ 150.3, 146.9, 136.5, 127.2, 122.1. ^{19}F NMR (377 MHz, acetone-d$_6$) δ -135.5. Spectroscopic data was consistent with the values reported in the literature.[9]

Tetrabutylammonium trifluoro(phenylethynyl)borate (7):

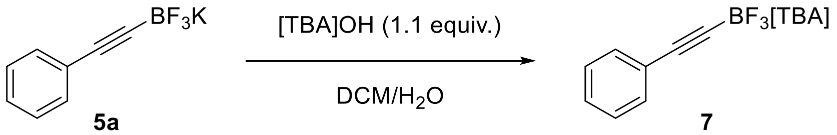

5a **7**

Synthesized following a reported procedure.[10] To a suspension of potassium trifluoro(phenylethynyl)borate (**5a**) (208 mg, 1.00 mmol, 1.00 equiv) in a mixture of DCM (4 mL) and water (1 mL) was added a tetrabutylammonium hydroxide solution (1.5 M, 0.72 mL, 1.1 mmol, 1.10 equiv) in water. The biphasic mixture was vigorously stirred for 1.5 h. The mixture was extracted with 3 × 10 mL of DCM. The combined organic layers were dried over $MgSO_4$, filtered and concentrated in vacuo. Addition of pentane to the oil obtained made the product precipitate. The suspension was concentrated in vacuo to afford tetrabutylammonium trifluoro(phenylethynyl)borate (**7**) (355 mg, 0.864 mmol, 86% yield) as a white solid.

^{1}H NMR (400 MHz, $CDCl_3$) δ 7.40 – 7.34 (m, 2H, Ar*H*), 7.23 – 7.14 (m, 3H, Ar*H*), 3.25 – 3.14 (m, 8H, NC*H$_2$*), 1.62 – 1.52 (m, 8H, C*H$_2$*), 1.39 (h, *J* = 7.3 Hz, 8H, C*H$_2$*), 0.94 (t, *J* = 7.3 Hz, 12H, CH_2C*H$_3$*). ^{13}C NMR (101 MHz, $CDCl_3$) δ 131.8, 128.0, 126.8, 126.1, 58.6, 24.0, 19.7, 13.7. ^{19}F NMR (376 MHz, $CDCl_3$) δ -133.8. Spectroscopic data was consistent with the values reported in the literature.[10]

[9] J. H. Song, P. Choi, S. E. Lee, K. H. Jeong, T. Kim, K. S. Kang, Y. S. Choi, J. Ham, *Eur. J. Org. Chem.* **2013**, *2013*, 6249–6253.
[10] Z. Zawada, Z. Guo, B. L. Oliveira, C. D. Navo, H. Li, P. M. S. D. Cal, F. Corzana, G. Jiménez-Osés, G. J. L. Bernardes, *Bioconjug. Chem.* **2021**, *32*, 1812–1822.

7. Annexes

The synthesis of all the compounds listed in this section as well as their corresponding characterization data were taken from previous publications of our group.[8,11,12,13,14]

Ref. 8: Compounds **5a**, **5c**, **5e**, **5f**, **5g**, **5i**, **5j**, **5m**, **5n**, **5o**, **16**, **17**.

Ref. 11: Compound **2**.

Ref. 12: Compounds **1n**, **1o**, **21**.

Ref. 13: Compounds **1q-w**, **3**, **4a-ah**, **5h**, **5k**, **5l**, **19**, **29**, **37**.

Ref. 14: Compounds **5d**, **33**, **34**.

7.1 Synthesis of HIRs previously reported by our group

1-Hydroxy-1,2-benziodoxol-3-(1*H*)-one (HOBX, 16):

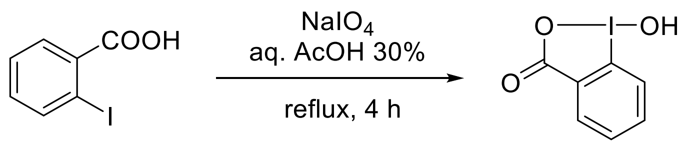

15 HOBX (**16**)

Following an adapted version of a reported procedure,[15] $NaIO_4$ (18.1 g, 84.7 mmol, 1.05 equiv) and 2-iodobenzoic acid (**15**) (20.0 g, 80.6 mmol, 1.00 equiv) were suspended in a mixture of AcOH (36 mL) and water (84 mL). The mixture was vigorously stirred and refluxed for 4 h. The reaction mixture was then diluted with cold water (100 mL) and allowed to cool to room temperature protected from light. The crude product was collected by filtration, washed on the filter with cold water (3 × 50 mL) and cold acetone (3 × 50 mL), and air-dried in the dark overnight to give the pure 1-hydroxy-1,2-benziodoxol-3-(1*H*)-one (HOBX, **16**) (20.0 g, 75.7 mmol, 94%) as a white solid.

^1H NMR (400 MHz, DMSO-d_6): δ 8.02 (dd, *J* = 7.7, 1.4 Hz, 1H, Ar*H*), 7.97 (m, 1H, Ar*H*), 7.85 (dd, *J* = 8.2, 0.7 Hz, 1H), 7.71 (td, *J* = 7.6, 1.2 Hz, 1H, Ar*H*); ^{13}C NMR (100 MHz, DMSO-d_6): δ 167.7, 134.5, 131.5, 131.1, 130.4, 126.3, 120.4. Spectroscopic data was consistent with the values reported in the literature.[15]

---

[11] S. G. E. Amos, S. Nicolai, J. Waser, *Chem. Sci.* **2020**, *11*, 11274–11279.
[12] P. Palamini, E. M. D. Allouche, J. Waser, *Org. Lett.* **2023**, *25*, 6791–6795.
[13] J. Borrel, J. Waser, *Chem. Sci.* **2023**, *14*, 9452–9460.
[14] P. Palamini, J. Borrel, M. Djaïd, M. Delattre, J. Waser, *Org. Lett.* **2023**, *25*, 7535–7539.
[15] L. Kraszkiewicz, L. Skulski, *Arkivoc* **2003**, *2003*, 120.

1-[Phenylethynyl]-1,2-benziodoxol-3-(1*H*)-one (Ph-EBX, 2):

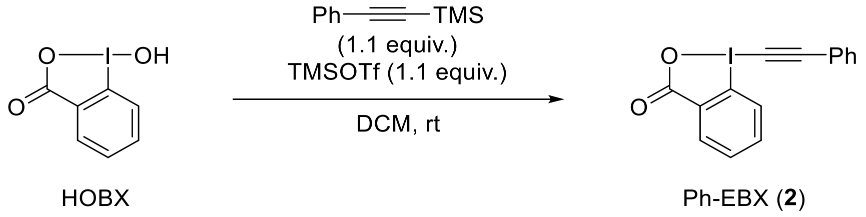

Following a reported procedure,[11] trimethylsilyltriflate (9.1 mL, 50 mmol, 1.1 equiv) was added dropwise to a suspension of HOBX (12.1 g, 45.8 mmol, 1.0 equiv) in CH_2Cl_2 (120 mL) at 0 °C. The mixture was stirred for 1 h, followed by the dropwise addition of trimethyl(phenylethynyl)silane (8.8 mL, 50 mmol, 1.1 equiv) (slightly exothermic). The resulting suspension was stirred for 6 h at rt, during this time a white solid was formed. A saturated solution of $NaHCO_3$ (120 mL) was added and the mixture was stirred vigorously for 30 min. The two layers of the mother liquors were separated and the organic layer was washed with sat. $NaHCO_3$ (2 × 50 mL), dried over $MgSO_4$, filtered and evaporated under reduced pressure. The resulting solid was recrystallized in EtOAc/MeOH (7:3) (ca. 20 mL). The solution was left to cool to rt then in the freezer overnight, filtered and dried under high vacuum to afford Ph-EBX (**2**) (6.8 g, 25 mmol, 43%) as colorless crystals.

^{1}H NMR (400 MHz, $CDCl_3$) δ 8.46 (m, 1H, Ar*H*), 8.28 (m, 1H, Ar*H*), 7.80 (m, 2H, Ar*H*), 7.63 (m, 2H, Ar*H*), 7.48 (m, 3H, Ar*H*). ^{13}C NMR (101 MHz, $CDCl_3$) δ 163.9, 134.9, 132.9, 132.5, 131.6, 131.3. 130.8, 128.8, 126.2, 120.5, 116.2, 106.6, 50.2. Spectroscopic data was consistent with the values reported in the literature.[11]

1-Acetoxy-1,2-benziodoxol-3-(1*H*)-one (AcOBX, 17):

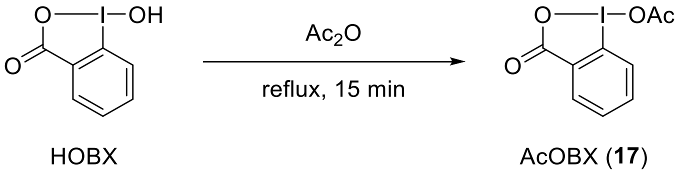

Following a reported procedure,[16] a suspension of HOBX (5.0 g, 19 mmol, 1 equiv) in acetic anhydride (19 mL) was refluxed until total dissolution (≈15 min). The resulting clears solution was allowed to cool to room temperature and then cooled to 5 °C in the fridge. The white crystals were filtered, washed with pentane (3 × 30 mL) and dried under reduced pressure to afford 1-acetoxy-1,2-benziodoxol-3-(1*H*)-one (AcOBX, **17**) (5.0 g, 16 mmol, 86%) as a white solid.

[16] E. Grenet, J. Waser, *Org. Lett.* **2018**, *20*, 1473–1476.

^1H NMR (400 MHz, $CDCl_3$) δ 8.26 (dd, *J* = 7.6, 1.6 Hz, 1H, Ar*H*), 8.01 (dd, *J* = 8.1, 0.9 Hz, 1H, Ar*H*), 7.96 – 7.90 (m, 1H, Ar*H*), 7.72 (td, *J* = 7.4, 1.0 Hz, 1H, Ar*H*), 2.26 (s, 3H, C*H*$_3$). Spectroscopic data was consistent with the values reported in the literature.[17]

3-Oxo-2-tosyl-2,3-dihydro-1*H*-1λ^3-benzo[*d*][1,2]iodazol-1-yl acetate (19):

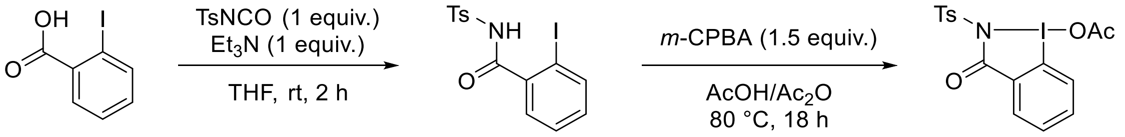

15 **18** **19**

Following a reported procedure,[18] an oven dried round-bottom flask charged with 2-iodobenzoic acid (**15**) (10 g, 40 mmol, 1.0 equiv) was evacuated and backfilled with N_2 (3×) then dry THF (115 mL) and *p*-toluenesulfonyl isocyanate (6.2 mL, 40 mmol, 1.0 equiv) were added. Finally, triethylamine (5.6 mL, 40 mmol, 1.0 equiv) was added dropwise under N_2, a slightly exothermic reaction began in addition to gas release. The reaction was stirred at rt for 2 h. The mixture was diluted with EtOAc (150 mL) and washed with 1 M aq. HCl (2 × 100 mL) and brine (50 mL). The organic layer was dried over $MgSO_4$, filtered and concentrated in vacuo. The crude product was used in the next step without further purification.

Following a reported procedure,[19] to a round-bottom flask charged with a solution of crude 2-iodo-*N*-tosylbenzamide (**18**) in a mixture of AcOH (70 mL) and Ac_2O (70 mL) was added *m*-CPBA (13.5 g, 60.5 mmol, 1.50 equiv, 77%). The reaction was stirred at 80 °C for 18 h covered from light. The mixture was left to cool to rt then pentane (100 mL) was added. The precipitate was filtered and washed with pentane (2 × 30 mL) and Et_2O (2 × 30 mL) and dried on the frit to afford 3-oxo-2-tosyl-2,3-dihydro-1*H*-1λ^3-benzo[*d*][1,2]iodazol-1-yl acetate (**19**) (7.74 g, 16.8 mmol, 42%) as an off white solid.

^1H NMR (400 MHz, $CDCl_3$) δ 8.12 (dd, *J* = 7.7, 1.6 Hz, 1H, Ar*H*), 8.04 (d, *J* = 8.3 Hz, 2H, Ar*H*), 7.99 (d, *J* = 8.4 Hz, 1H, Ar*H*), 7.87 – 7.81 (m, 1H, Ar*H*), 7.67 – 7.62 (m, 1H, Ar*H*), 7.33 (d, *J* = 8.1 Hz, 2H, Ar*H*), 2.42 (s, 3H, C*H*$_3$), 2.25 (s, 3H, C*H*$_3$). ^{13}C NMR (101 MHz, $CDCl_3$) δ 176.3, 162.3, 145.2, 136.4, 135.8, 133.0, 132.5, 131.3, 129.8, 129.8, 128.7, 116.8, 21.8, 20.9. Spectroscopic data was consistent with the values reported in the literature.[19]

1-Azido-2-tosyl-1,2-dihydro-3*H*-1λ^3-benzo[*d*][1,2]iodazol-3-one (Ts-ABZ, 3):

Caution: Even though Ts-ABZ has a much safer safety profile than the most commonly used azidobenziodoxolone (ABX) care has to be taken when preparing it.[20] The synthesis and filtration were carried out behind a blast shield wearing anti cut gloves (HyFlex 11-541) below regular nitrile gloves. The scale described in the procedure below was the largest scale the

---

[17] S. Bertho, R. Rey-Rodriguez, C. Colas, P. Retailleau, I. Gillaizeau, *Chem. - Eur. J.* **2017**, *23*, 17674–17677.
[18] V. Smyrnov, B. Muriel, J. Waser, *Org. Lett.* **2021**, *23*, 5435–5439.
[19] X.-G. Yang, F.-H. Du, J.-J. Li, C. Zhang, *Chem. - Eur. J.* **2022**, *28*, e202200272.
[20] S. Alazet, J. Preindl, R. Simonet-Davin, S. Nicolai, A. Nanchen, T. Meyer, J. Waser, *J. Org. Chem.* **2018**, *83*, 12334–12356.

reaction was carried on. To synthesize larger amount of the reagent we performed the reaction in multiple batches in parallel and filtered them individually. Ts-ABZ batches were stored in plastic containers and kept in the fridge at 4 °C. During the course of this project this synthesis was performed 34 times without incident with an average yield of 86%.

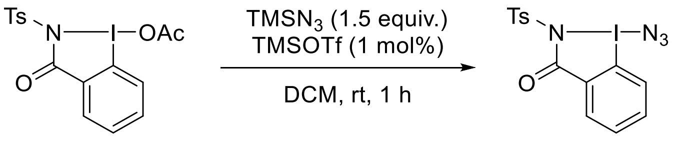

Following a reported procedure,[18] an oven dried flask containing a solution of 3-oxo-2-tosyl-2,3-dihydro-1*H*-1λ^3-benzo[*d*][1,2]iodazol-1-yl acetate (**19**) (919 mg, 2.00 mmol, 1.0 equiv) in dry DCM (10 mL) was cooled to 0 °C then $TMSN_3$ (0.40 mL, 3.0 mmol, 1.5 equiv) was added dropwise followed by two drops of TMSOTf (approximation: 4.0 µL, 20 µmol, 1 mol%). The reaction was stirred at rt for 1 h and pentane (30 mL) was added to induce further precipitation (usually precipitation already starts to occur during the reaction). The solid was filtered, washed with pentane (3 × 20 mL) and dried on the frit for 2 min to afford Ts-ABZ (778 mg, 1.76 mmol, 88% yield) as an off white solid.

^{1}H NMR (400 MHz, $CDCl_3$) δ 8.17 (dd, *J* = 7.6, 1.6 Hz, 1H, Ar*H*), 8.07 – 8.00 (m, 3H, Ar*H*), 7.93 – 7.86 (m, 1H, Ar*H*), 7.70 (td, *J* = 7.6, 0.7 Hz, 1H, Ar*H*), 7.33 (d, *J* = 8.1 Hz, 2H, Ar*H*), 2.42 (s, 3H, C*H*$_3$). ^{13}C NMR (101 MHz, $CDCl_3$) δ 161.3, 145.1, 136.5, 136.0, 133.0, 132.8, 131.8, 129.8, 128.6, 127.3, 116.2, 21.8.

7.2 Synthesis of alkenes previously reported by our group

*N*HBoc-Dha-*O*Me (21):

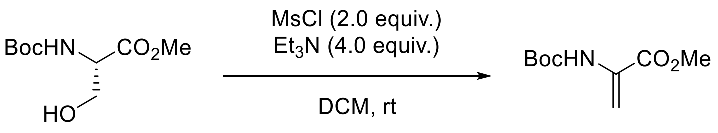

Following a reported procedure,[21] MsCl (0.73 mL, 9.4 mmol, 2.0 equiv) was added dropwise to a solution of Boc-Ser-OMe (**20**) (1.00 mL, 4.69 mmol, 1.0 equiv) and Et_3N (2.61 mL, 18.8 mmol, 4.0 equiv) in DCM (28 mL). The reaction was stirred at rt for 3 h, then quenched with a sat. sol. of $NaHCO_3$ and extracted with DCM (3 × 30 mL). The combined organic layer was dried over anhydrous $MgSO_4$ and concentrated in vacuo. The crude product was purified by column chromatography (pentane/EtOAc, 100:0 to 8:2) to afford *N*HBoc-Dha-*O*Me (**21**) (940 mg, 4.67 mmol, 100%) as a colorless oil.

^{1}H NMR (400 MHz, $CDCl_3$) δ 7.00 (s, 1H, N*H*), 6.15 (s, 1H, C=C*H*$_2$), 5.72 (d, *J* = 1.5 Hz, 1H, C=C*H*$_2$), 3.82 (s, 3H, OC*H*$_3$), 1.47 (s, 9H, C(C*H*$_3$)$_3$). ^{13}C NMR (101 MHz, $CDCl_3$) δ 164.6, 152.7,

[21] N. Sabat, F. Soualmia, P. Retailleau, A. Benjdia, O. Berteau, X. Guinchard, *Org. Lett.* **2020**, *22*, 4344–4349.

131.4, 105.3, 80.8, 53.0, 28.4. Spectroscopic data was consistent with the values reported in the literature.[22]

NBoc$_2$-Dha-OMe (1n):

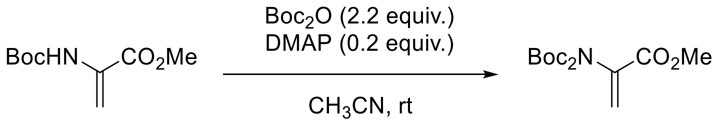

Following a reported procedure,[23] to a solution of *N*HBoc-Dha-*O*Me (**21**) (1.18 g, 5.86 mmol, 1.00 equiv) in CH_3CN (59 mL) were added Boc_2O (2.81 g, 12.9 mmol, 2.20 equiv) and DMAP (143 mg, 1.17 mmol, 0.20 equiv). The reaction was stirred at rt for 16 h. The solvent was removed under reduced pressure and the crude material was purified by column chromatography (pentane/EtOAc, 9:1) to afford NBoc$_2$-Dha-OMe (**1n**) (1.43 g, 4.75 mmol, 81%) as a white solid.

^{1}H NMR (400 MHz, $CDCl_3$) δ 6.34 (s, 1H, C=C*H$_2$*), 5.64 (s, 1H, C=C*H$_2$*), 3.79 (s, 3H, OC*H$_3$*), 1.46 (s, 18H, C(C*H$_3$*)$_3$). ^{13}C NMR (101 MHz, $CDCl_3$) δ 164.2, 150.8, 136.2, 124.8, 83.3, 52.5, 28.0. Spectroscopic data was consistent with the values reported in the literature.[23]

***N*HFmoc-Dha-*O*Me (1o):**

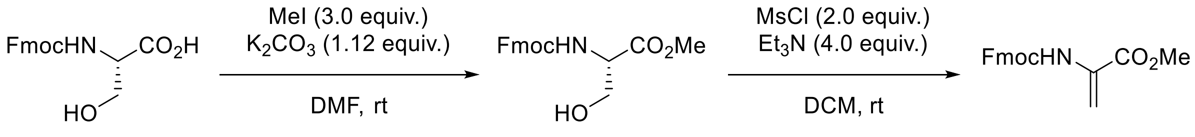

Following a reported procedure,[24] Fmoc-Ser-OH (**22**) (1.00 g, 3.10 mmol, 1.00 equiv) and potassium carbonate (498 mg, 3.60 mmol, 1.12 equiv) were dissolved in DMF (10 mL). The mixture was stirred for 30 min at rt and cooled to 0 °C. Then, methyl iodide (0.56 mL, 9.0 mmol, 3.00 equiv) was added. After 16 h, the reaction mixture was diluted with water (100 mL) and extracted with Et_2O (3 × 50 mL). The organic layer was combined, dried over $MgSO_4$ and concentrated under reduced pressure to afford Fmoc-Ser-OMe (**23**). The crude product was used without any further purification.

Following a reported procedure,21 MsCl (0.47 mL, 6.1 mmol, 2.0 equiv) was added dropwise to a solution of crude **23** and Et_3N (1.73 mL, 12.4 mmol, 4.0 equiv) in DCM (18 mL). The reaction was stirred at rt for 3 hours, then quenched with a sat. sol. of $NaHCO_3$ and extracted with DCM (3 × 20 mL). The combined organic layer was dried over anhydrous $MgSO_4$ and concentrated in vacuo. The crude product was purified by column chromatography

[22] J.-A. Shin, J. Kim, H. Lee, S. Ha, H.-Y. Lee, *J. Org. Chem.* **2019**, *84*, 4558–4565.
[23] R. Petracca, K. A. Bowen, L. McSweeney, S. O'Flaherty, V. Genna, B. Twamley, M. Devocelle, E. M. Scanlan, *Org. Lett.* **2019**, *21*, 3281–3285.
[24] F. Zhang, W. Zhang, Y. Zhang, D. P. Curran, G. Liu, *J. Org. Chem.* **2009**, *74*, 2594–2597.

(pentane/EtOAc, 100:0 to 8:2) to afford *N*HFmoc-Dha-*O*Me (**1o**) (527 mg, 1.63 mmol, 53%) as a white solid.

R_f (pentane/EtOAc, 95:5): 0.23; ^{1}H NMR (400 MHz, $CDCl_3$) δ 7.78 (d, 2H, Ar*H*), 7.60 (d, *J* = 0.9 Hz, 2H, Ar*H*), 7.42 (t, *J* = 0.9 Hz, 2H, Ar*H*), 7.33 (td, *J* = 7.4, 1.2 Hz, 2H, Ar*H*), 6.24 (s, 1H, C=C*H₂*), 5.80 (s, 1H, C=C*H₂*), 4.46 (d, *J* = 7.0 Hz, 2H, OC*H₂*), 4.26 (t, 1H, OCH_2CH), 3.86 (s, 3H, OC*H₃*). ^{13}C NMR (101 MHz, $CDCl_3$) δ 164.5, 158.0, 143.8, 141.5, 131.1, 128.0, 127.3, 125.2, 120.2, 106.4, 67.3, 53.2, 47.1. Spectroscopic data was consistent with the values reported in the literature.[25]

But-3-en-1-yn-1-ylbenzene (1q):

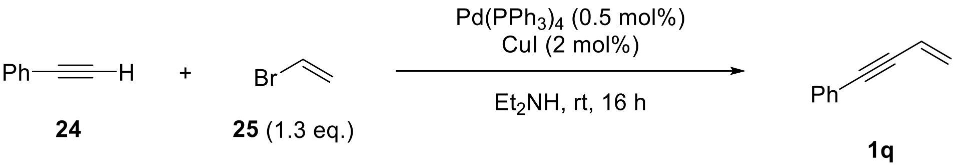

Compound 1b was synthesized following a reported procedure.[26] An oven dried round-bottom flask charged with CuI (19 mg, 0.10 mmol, 2 mol%) and $Pd(PPh_3)_4$ (30 mg, 25 µmol, 0.5 mol%) was evacuated and backfilled with N_2 (3×) then degassed Et_2NH (2.5 mL) was added. The mixture was cooled to 0 °C and phenylacetylene (**24**) (0.55 mL, 5.0 mmol, 1.00 equiv) and a solution of vinyl bromide (**25**) (6.5 mL, 6.5 mmol, 1 M in THF, 1.30 equiv) were added. The reaction was stirred at rt for 16 h under N_2 atmosphere. The reaction was quenched with water (≈15 mL) then extracted with 3 × 15 mL of a mixture of pentane/Et_2O (1:1). The combined organic layers were washed with 20 mL of 1 M aq. HCl, dried over $MgSO_4$, filtered and concentrated in vacuo. The crude product was loaded on celite and purified by column chromatography (pentane) to afford but-3-en-1-yn-1-ylbenzene (**1q**) (566 mg, 4.42 mmol, 88%) as a colorless oil.

^{1}H NMR (400 MHz, $CDCl_3$) δ 7.49 – 7.41 (m, 2H, Ar*H*), 7.35 – 7.29 (m, 3H, Ar*H*), 6.03 (dd, *J* = 17.5, 11.1 Hz, 1H, *H*C=CH_2), 5.74 (dd, *J* = 17.5, 2.1 Hz, 1H, HC=C*H₂*), 5.55 (dd, *J* = 11.1, 2.1 Hz, 1H, HC=C*H₂*). ^{13}C NMR (101 MHz, $CDCl_3$) δ 131.7, 128.4, 128.4, 127.0, 123.3, 117.3, 90.1, 88.2. Spectroscopic data was consistent with the values reported in the literature.[26]

1-(Trifluoromethoxy)-4-vinylbenzene (1r):

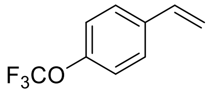

1r

Synthesized following **general procedure A** starting from 4-(trifluoromethoxy)benzaldehyde (0.57 mL, 4.0 mmol). The reaction was carried out for 1 h and extractions were performed with

[25] S. Koch, D. Schollmeyer, H. Löwe, H. Kunz, *Chem. - Eur. J.* **2013**, *19*, 7020–7041.
[26] Y. Zhang, B. Yu, B. Gao, T. Zhang, H. Huang, *Org. Lett.* **2019**, *21*, 535–539.

Et_2O. The crude product was loaded on celite and purified by column chromatography (pentane) to afford 1-(trifluoromethoxy)-4-vinylbenzene (**1r**) (441 mg, 2.34 mmol, 59%) as a colorless oil.

^{1}H NMR (400 MHz, $CDCl_3$) δ 7.46 – 7.39 (m, 2H, Ar*H*), 7.17 (d, *J* = 8.1 Hz, 2H, Ar*H*), 6.70 (dd, *J* = 17.6, 10.9 Hz, 1H, *H*C=CH_2), 5.73 (dd, *J* = 17.6, 0.5 Hz, 1H, HC=C*H₂*), 5.29 (dd, *J* = 10.9, 0.4 Hz, 1H, HC=CH_2). ^{13}C NMR (101 MHz, $CDCl_3$) δ 148.8 (m), 136.4, 135.6, 127.6, 121.2, 120.6 (q, *J* = 257.0 Hz), 115.0. ^{19}F NMR (376 MHz, $CDCl_3$) δ -57.9. Spectroscopic data was consistent with the values reported in the literature.[27]

N-(4-Vinylphenyl)acetamide (1s):

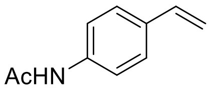

1s

Synthesized following **general procedure A** starting from 4-acetamidobenzaldehyde (653 mg, 4.00 mmol). The reaction was carried out for 3.5 h and extractions were performed with EtOAc. The crude product was purified by column chromatography (pentane/EtOAc, 6:4) to afford *N*-(4-vinylphenyl)acetamide (**1s**) (520 mg, 3.00 mmol, 75%) as a white solid.

^{1}H NMR (400 MHz, $CDCl_3$) δ 7.47 (d, *J* = 8.5 Hz, 2H, Ar*H*), 7.39 – 7.30 (m, 3H, Ar*H* + N*H*Ac), 6.67 (dd, *J* = 17.6, 10.9 Hz, 1H, *H*C=CH_2), 5.68 (d, *J* = 17.6 Hz, 1H, HC=CH_2), 5.19 (d, *J* = 10.9 Hz, 1H, HC=CH_2), 2.17 (s, 3H, CH_3). ^{13}C NMR (101 MHz, $CDCl_3$) δ 168.4, 137.6, 136.2, 133.9, 127.0, 119.9, 113.2, 24.8. Spectroscopic data was consistent with the values reported in the literature.[28]

4-Vinylphenol (1t):

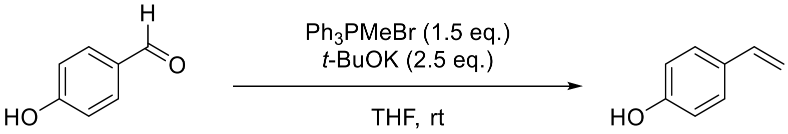

26 **1t**

Compound **1s** was synthesized following a reported procedure.[29] An oven dried round-bottom flask charged with methyltriphenylphosphonium bromide (2.14 g, 6.00 mmol, 1.50 equiv) and potassium *tert*-butoxide (1.12 g, 10.0 mmol, 2.50 equiv) was evacuated and backfilled with N_2. Dry THF (11 mL) was added and the mixture was stirred at rt for 30 min. A solution of 4-hydroxybenzaldehyde (**26**) (488 mg, 4.00 mmol, 1.00 equiv) in dry THF (5 mL) was added dropwise over 5 min and the reaction was stirred at rt for 3.5 h under N_2. The reaction was quenched with 35 mL of a sat. sol. of NH_4Cl and the mixture was extracted with 3 × 40 mL of

---

[27] M. Su, X. Huang, C. Lei, J. Jin, *Org. Lett.* **2022**, *24*, 354–358.
[28] M.-J. Zhou, L. Zhang, G. Liu, C. Xu, Z. Huang, *J. Am. Chem. Soc.* **2021**, *143*, 16470–16485.
[29] F. C. Demidoff, F. P. de Souza, C. D. Netto, *Synthesis* **2017**, *49*, 5217–5223.

Et_2O or EtOAc. The combined organic layers were washed with brine, dried over $MgSO_4$, filtered and concentrated in vacuo. The crude product was purified by column chromatography (pentane/EtOAc, 9:1) to afford 4-vinylphenol (**1t**) (388 mg, 3.23 mmol, 81%) as a white solid.

^{1}H NMR (400 MHz, $CDCl_3$) δ 7.34 – 7.28 (m, 2H, Ar*H*), 6.82 – 6.77 (m, 2H, Ar*H*), 6.65 (dd, *J* = 17.6, 10.9 Hz, 1H, *H*C=CH_2), 5.61 (dd, *J* = 17.6, 0.8 Hz, 1H, HC=CH_2), 5.13 (dd, *J* = 10.9, 0.8 Hz, 1H, HC=CH_2), 4.86 (s, 1H, O*H*). ^{13}C NMR (101 MHz, $CDCl_3$) δ 155.4, 136.3, 130.8, 127.8, 115.5, 111.8. Spectroscopic data was consistent with the values reported in the literature.[28]

2-Vinylbenzofuran (1u):

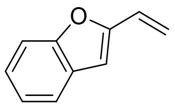

1u

Synthesized following **general procedure A** starting from 2-benzofurancarboxaldehyde (0.49 mL, 4.0 mmol). The reaction was carried out for 1 h and extractions were performed with Et_2O. The crude product was loaded on celite and purified by column chromatography (pentane) to afford 2-vinylbenzofuran (**1u**) (465 mg, 3.23 mmol, 81%) as a colorless oil.

^{1}H NMR (400 MHz, $CDCl_3$) δ 7.55 – 7.50 (m, 1H, Ar*H*), 7.48 – 7.44 (m, 1H, Ar*H*), 7.31 – 7.24 (m, 1H, Ar*H*), 7.20 (td, *J* = 7.5, 1.0 Hz, 1H, Ar*H*), 6.65 (dd, *J* = 17.5, 11.3 Hz, 1H, *H*C=CH_2), 6.60 (s, 1H, OC=C*H*), 5.97 (dd, *J* = 17.5, 0.7 Hz, 1H, HC=CH_2), 5.39 (dd, *J* = 11.2, 1.0 Hz, 1H, HC=CH_2). ^{13}C NMR (101 MHz, $CDCl_3$) δ 155.0, 154.9, 129.0, 125.4, 124.8, 122.9, 121.1, 115.9, 111.2, 104.9. Spectroscopic data was consistent with the values reported in the literature.[30]

Note: Product was stored at −20 °C after isolation.

2-Bromo-5-vinylfuran (1v):

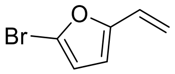

1v

Synthesized following **general procedure A** starting from 5-bromo-2-furaldehyde (700 mg, 4.00 mmol). The reaction was carried out for 1 h and extractions were performed with Et_2O. The crude product was loaded on celite and purified by column chromatography (pentane) to afford 2-bromo-5-vinylfuran (**1v**) (491 mg, 2.84 mmol, 71%) as a light orange oil.

^{1}H NMR (400 MHz, $CDCl_3$) δ 6.40 (dd, *J* = 17.5, 11.3 Hz, 1H, *H*C=CH_2), 6.29 (d, *J* = 3.3 Hz, 1H, Ar*H*), 6.21 (d, *J* = 3.3 Hz, 1H, Ar*H*), 5.65 (dd, *J* = 17.4, 0.7 Hz, 1H, HC=CH_2), 5.17 (dd, *J*

[30] H. Seo, A. Liu, T. F. Jamison, *J. Am. Chem. Soc.* **2017**, *139*, 13969–13972.

= 11.3, 0.9 Hz, 1H, HC=C*H₂*). ^{13}C NMR (101 MHz, $CDCl_3$) δ 155.3, 124.3, 121.8, 113.1, 113.0, 110.4. Spectroscopic data was consistent with the values reported in the literature.[31]

Note: Product was stored at −20 °C after isolation. It was used the next day in the azido-alkynylation reaction. We observed substantial degradation after 2-3 days of storage.

2*H*-Chromene (1w):

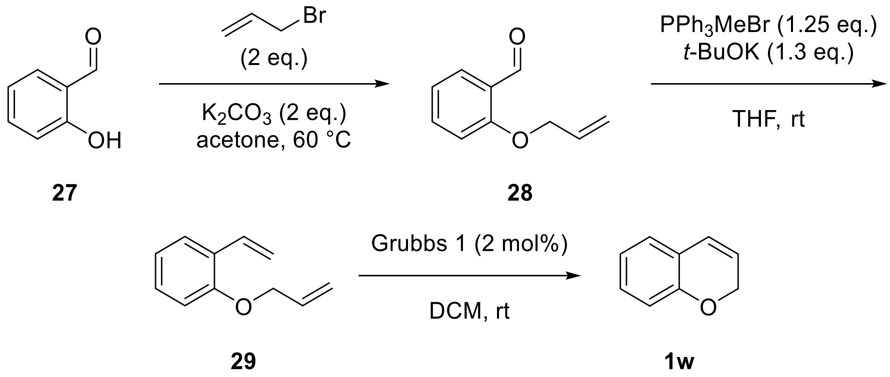

Following a reported procedure,[1,32] to a round-bottom flask charged with a suspension of K_2CO_3 (4.15 g, 30.0 mmol, 2.0 equiv) in acetone (45 mL) were added 2-hydroxybenzaldehyde (**27**) (1.6 mL, 15 mmol, 1.0 equiv) and allyl bromide (2.6 mL, 30 mmol, 2.0 equiv). The reaction was heated to 60 °C for 3 h. The mixture was filtered over a plug of celite, eluted with acetone and concentrated in vacuo to afford 2-(allyloxy)benzaldehyde (**28**). The crude product was used in the next step without further purification.

An oven dried round-bottom flask charged with methyltriphenylphosphonium bromide (6.7 g, 19 mmol, 1.25 equiv) and potassium *tert*-butoxide (2.2 g, 20 mmol, 1.30 equiv) was evacuated and backfilled with N_2. Dry THF (45 mL) was added and the mixture was stirred at rt for 30 min. A solution of crude aldehyde **28** previously prepared in dry THF (15 mL) was added dropwise over 5 min and the reaction was stirred at rt under N_2 for 1 h. The reaction was quenched with 60 mL of a sat. sol. of NH_4Cl and the mixture was extracted with 3 × 60 mL of EtOAc. The combined organic layers were washed with brine, dried over $MgSO_4$, filtered and concentrated in vacuo. The crude product was loaded on celite and purified by column chromatography (pentane) to afford 1-(allyloxy)-2-vinylbenzene (**29**) (1.78 g, 11.1 mmol, 74% over 2 steps) as a colorless oil.

^{1}H NMR (400 MHz, $CDCl_3$) δ 7.49 (dd, *J* = 7.6, 1.7 Hz, 1H, Ar*H*), 7.21 (ddd, *J* = 8.3, 7.5, 1.7 Hz, 1H, Ar*H*), 7.10 (dd, *J* = 17.8, 11.2 Hz, 1H, ArC*H*=CH_2), 6.97 – 6.91 (m, 1H, Ar*H*), 6.87 (dd, *J* = 8.3, 0.8 Hz, 1H, Ar*H*), 6.14 – 6.03 (m, 1H, CH_2C*H*=CH_2), 5.75 (dd, *J* = 17.8, 1.5 Hz, 1H, ArCH=CH_2), 5.43 (dq, *J* = 17.3, 1.7 Hz, 1H, CH_2CH=CH_2), 5.32 – 5.23 (m, 2H, CH_2CH=CH_2 + ArCH=CH_2), 4.57 (dt, *J* = 5.1, 1.6 Hz, 2H, OCH_2CH). ^{13}C NMR (101 MHz, $CDCl_3$) δ 155.9,

[31] Y. Yamamoto, Y. Yamada, H. Sajiki, Y. Sawama, *Bull. Chem. Soc. Jpn.* **2020**, *93*, 1419–1423.
[32] X.-S. Liang, R.-D. Li, W. Sun, Z. Liu, X.-C. Wang, *ACS Catal.* **2022**, *12*, 9153–9158.

133.5, 131.8, 128.9, 127.2, 126.7, 121.0, 117.5, 114.5, 112.5, 69.3. Spectroscopic data was consistent with the values reported in the literature.[33]

Following a reported procedure,[32] to an oven-dried round-bottom flask containing a solution of 1-(allyloxy)-2-vinylbenzene (**29**) (320 mg, 2.00 mmol, 1.0 equiv) in dry DCM (10 mL) was added Grubbs 1 catalyst (33 mg, 40 µmol, 0.02 equiv). The reaction was stirred at rt for 2 h. The crude mixture was concentrated in vacuo, loaded on celite and purified by column chromatography (pentane/Et_2O, 100:0 to 97:3) to afford 2*H*-chromene (**1w**) (180 mg, 1.36 mmol, 68%) as a colorless oil.

^{1}H NMR (400 MHz, $CDCl_3$) δ 7.10 (td, *J* = 7.8, 1.7 Hz, 1H, Ar*H*), 6.96 (dd, *J* = 7.4, 1.7 Hz, 1H, Ar*H*), 6.86 (td, *J* = 7.4, 1.1 Hz, 1H, Ar*H*), 6.77 (d, *J* = 8.1 Hz, 1H, Ar*H*), 6.42 (dt, *J* = 9.9, 1.6 Hz, 1H, ArC*H*=CH), 5.77 (dt, *J* = 9.8, 3.6 Hz, 1H,CH=C*H*CH_2), 4.82 (dd, *J* = 3.6, 1.9 Hz, 2H, OCH_2CH). ^{13}C NMR (101 MHz, $CDCl_3$) δ 154.2, 129.3, 126.7, 124.7, 122.5, 122.1, 121.5, 115.9, 65.7. Spectroscopic data was consistent with the values reported in the literature.[33]

7.3 Synthesis of potassium trifluoroborate salts previously reported by our group

Potassium trifluoro(phenylethynyl)borate (5a):

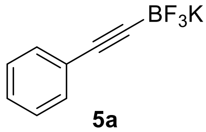

5a

Synthesized following **general procedure B** starting from phenylacetylene (1.53 g, 1.65 mL, 15.0 mmol). Potassium trifluoro(phenylethynyl)borate (**5a**) (2.60 g, 12.5 mmol, 83%) was obtained as a white solid.

^{1}H NMR (400 MHz, acetone-d_6) δ 7.35 – 7.29 (m, 2H, Ar*H*), 7.27 – 7.17 (m, 3H, Ar*H*). ^{13}C NMR (101 MHz, acetone-d_6) δ 132.1, 128.8, 127.4, 127.2. ^{19}F NMR (376 MHz, acetone-d_6) δ -135.0. Spectroscopic data was consistent with the values reported in the literature.[34]

Potassium trifluoro(((8*R*,9*S*,13*S*,14*S*,17*S*)-17-hydroxy-3-methoxy-13-methyl-7,8,9,11,12,13,14,15,16,17-decahydro-6*H*-cyclopenta[*a*]phenanthren-17-yl)ethynyl)borate (5c):

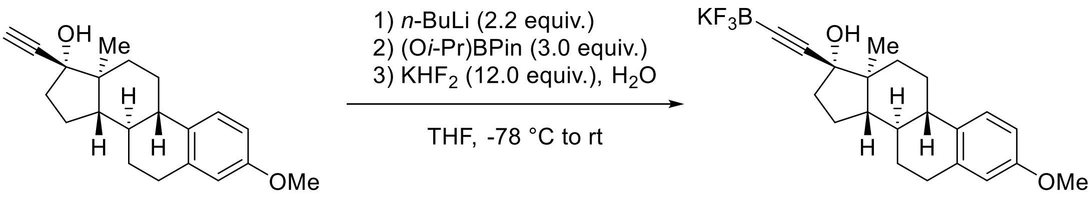

30 **5c**

---

[33] F. Yang, K. Rauch, K. Kettelhoit, L. Ackermann, *Angew. Chem. Int. Ed.* **2014**, *53*, 11285–11288.
[34] G. A. Molander, B. W. Katona, F. Machrouhi, *J. Org. Chem.* **2002**, *67*, 8416–8423.

Compound **5c** was synthesized following an adapted version of a reported procedure.[35] An oven-dried round-bottom flask (PFA) charged with (8*R*,9*S*,13*S*,14*S*,17*R*)-17-ethynyl-3-methoxy-13-methyl-7,8,9,11,12,13,14,15,16,17-decahydro-6*H*-cyclopenta[a]phenanthren-17-ol (**30**) (1.0 g, 3.2 mmol, 1.0 equiv) was evacuated and backfilled with N_2 (3×). Dry THF (22 mL) was added, the mixture was cooled to −78 °C and a solution of *n*-BuLi (2.8 mL, 7.1 mmol, 2.5 M, 2.2 equiv) in hexane was added dropwise under N_2. The reaction was stirred at −78 °C for 1 h and 2-isopropoxy-4,4,5,5-tetramethyl-1,3,2-dioxaborolane (2.0 mL, 9.7 mmol, 3.0 equiv) was added quickly. The reaction was warmed to −20 °C and stirred for 1 h. A saturated solution of KHF_2 (3.02 g, 38.7 mmol, 12.0 equiv) in water (9 mL + additional 9 mL to rinse the remaining solid) was added. The reaction was stirred at rt open air for 1 h then concentrated in vacuo. The wet solid obtained was further dried by co-evaporation with acetone. To the dry solid was added acetone (≈30 mL) and the resulting mixture was placed on a rotary evaporator and rotated rapidly at atmospheric pressure with the bath set at 45 °C for 15 minutes. The flask was removed and the mixture carefully filtered taking care to leave the insoluble material in the reaction flask. Acetone was once again added and the process (heating for 15 min then collection of the liquid) was repeated 2 more times. The combined acetone filtrates were concentrated in vacuo to approximately 1/3 of the initial volume. Et_2O (≈40 mL) was added causing a white solid to precipitate. The mixture was cooled to 0 °C for 10 min then filtered. The solid obtained was washed with Et_2O, dried in vacuo then recrystallized in acetone using Et_2O to induce precipitation to afford potassium trifluoro(((8*R*,9*S*,13*S*,14*S*,17*S*)-17-hydroxy-3-methoxy-13-methyl-7,8,9,11,12,13,14,15,16,17-decahydro-6*H*-cyclopenta[*a*]phenanthren-17-yl)ethynyl)borate (**5c**) (0.53 g, 1.3 mmol, 40%) as a white solid.

Mp (Dec.): 257 °C; ^{1}H NMR (400 MHz, DMSO-d_6) δ 7.18 (d, *J* = 8.6 Hz, 1H, Ar*H*), 6.67 (dd, *J* = 8.6, 2.8 Hz, 1H, Ar*H*), 6.59 (d, *J* = 2.7 Hz, 1H, Ar*H*), 4.79 (s, 1H, O*H*), 3.69 (s, 3H, OCH_3), 2.77 (q, *J* = 4.3 Hz, 2H, CH_2), 2.38 – 2.23 (m, 1H, CH_2), 2.02 (ddd, *J* = 12.9, 9.9, 6.1 Hz, 2H, CH_2 + C*H*), 1.91 (td, *J* = 13.2, 4.2 Hz, 1H, CH_2), 1.85 – 1.72 (m, 2H, CH_2), 1.72 – 1.52 (m, 3H, CH_2 + C*H*), 1.35 – 1.17 (m, 4H, CH_2 + C*H*), 0.71 (s, 3H, C-CH_3). ^{13}C NMR (101 MHz, DMSO-d_6)[36] δ 157.0, 137.4, 132.3, 126.2, 113.4, 111.5, 78.2, 54.9, 48.6, 46.5, 43.3, 32.6, 29.4, 27.0, 26.3, 22.6, 13.0. ^{19}F NMR (376 MHz, DMSO-d_6) δ -130.6. Spectroscopic data was consistent with the values reported in the literature.[8]

Potassium (3-(4-chlorophenyl)-3-oxoprop-1-yn-1-yl)trifluoroborate (5d):

[35] J. D. Kirkham, S. J. Edeson, S. Stokes, J. P. A. Harrity, *Org. Lett.* **2012**, *14*, 5354–5357.
[36] Two carbon signals are hidden under the DMSO signal.

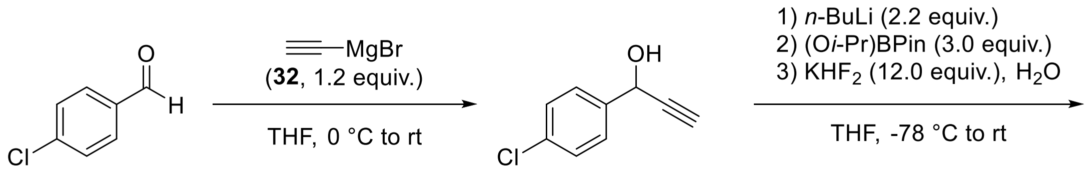

31 **33**

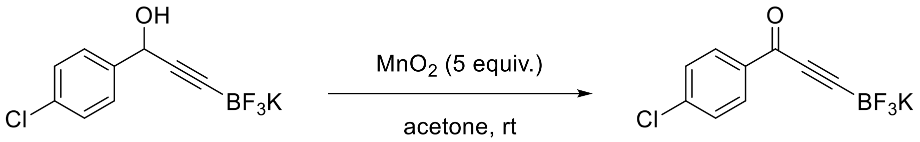

34 **5d**

Following a reported procedure,[37] an oven-dried round-bottom flask, charged with 4-chlorobenzaldehyde (**31**) (1.05 g, 7.50 mmol, 1.0 equiv) was evacuated and backfilled with N_2 (3×). Then, dry THF (22 mL) were added. The mixture was cooled to 0 °C and a solution of ethynylmagnesium bromide (**32**) (0.50 M in THF, 18 mL, 9.0 mmol, 1.2 equiv) was added dropwise under N_2. The mixture was allowed to slowly warm to rt and the reaction was stirred for 3 h. Upon completion, the reaction was quenched with saturated NH_4Cl (8 mL) and extracted with Et_2O (3 × 20 mL). The combined organic layers were washed with brine, dried over $MgSO_4$ and filtered. The solvent was removed under vacuum, and the residue was purified by column chromatography (pentane/EtOAc, 8:2) to afford 1-(chlorophenyl)prop-2-yn-1-ol (**33**) (1.01 g, 6.07 mmol, 81%) as an orange oil.

^{1}H NMR (400 MHz, $CDCl_3$) δ 7.48 – 7.38 (m, 2H, Ar*H*), 7.37 – 7.28 (m, 2H, Ar*H*), 5.38 (d, *J* = 2.4 Hz, 1H, (OH)C*H*C≡C), 3.18 (s, 1H, C≡C*H*), 2.67 (d, *J* = 2.3 Hz, 1H, O*H*). ^{13}C NMR (101 MHz, $CDCl_3$) δ 138.4, 134.3, 128.8, 128.1, 83.1, 75.3, 63.6. Spectroscopic data was consistent with the values reported in the literature.[38]

Following a reported procedure,[39] an oven-dried round bottom flask (PFA) was evacuated and backfilled with N_2 (3×), then 1-(chlorophenyl)prop-2-yn-1-ol (**33**) (1.25 g, 7.50 mmol, 1.0 equiv) and dry THF (20 mL) were added. The solution was cooled to −78 °C and a solution of *n*-BuLi (2.5 M in hexane, 6.60 mL, 16.5 mmol, 2.2 equiv) was added dropwise under N_2. The mixture was stirred at this temperature for 1 h. 4,4,5,5-Tetramethyl-2-propan-2-yloxy-1,3,2-dioxaborolane (4.6 mL, 23 mmol, 3.0 equiv) was added at once at −78 °C and the mixture was allowed to warm to −20 °C (ice/NaCl bath) and stirred for 1 h. A solution of KHF_2 (7.0 g, 90 mmol, 12.0 equiv) in water (13 mL + additional 7 mL to rinse the remaining solid) was added. The reaction was stirred at rt open to air for 1 h. The mixture was concentrated in vacuo, the wet solid obtained was further dried by co-evaporation with toluene (3×). The resulting solid was diluted with acetone (≈30 mL) and was put on the rotavap at P_{atm} with the bath at 45 °C for 10 minutes. The solution was filtered with care to leave the insoluble material in the flask.

[37] D. A. Petrone, M. Isomura, I. Franzoni, S. L. Rössler, E. M. Carreira, *J. Am. Chem. Soc.* **2018**, *140*, 4697–4704.
[38] C.-F. Xu, M. Xu, L.-Q. Yang, C.-Y. Li, *J. Org. Chem.* **2012**, *77*, 3010–3016.
[39] P. Fricero, L. Bialy, A. W. Brown, W. Czechtizky, M. Méndez, J. P. A. Harrity, *J. Org. Chem.* **2017**, *82*, 1688–1696.

This process was repeated 2 more times. Solvents were removed in vacuo. The obtained solid was dissolved in the minimum amount of hot acetone and precipitation of the desired product was performed by addition of diethyl ether. The mixture was cooled down to 0 °C, filtered off, washed with diethyl ether (3 × 20 mL) and dried in vacuo to afford potassium (3-(4-chlorophenyl)-3-hydroxyprop-1-yn-1-yl)trifluoroborate (**34**) (1.64 g, 6.02 mmol, 80%) as a white solid.

^{1}H NMR (400 MHz, DMSO-d$_6$) δ 7.49 – 7.43 (m, 2H, Ar*H*), 7.37 (d, *J* = 8.7 Hz, 2H, Ar*H*), 5.70 (d, *J* = 6.0 Hz, 1H, C*H*OH), 5.16 (dd, *J* = 5.9, 1.7 Hz, 1H, O*H*). ^{13}C NMR (101 MHz, DMSO-d$_6$) δ 142.7, 131.4, 128.3, 127.8, 62.4. ^{19}F NMR (376 MHz, DMSO-d$_6$) δ -131.6. Spectroscopic data were consistent with the values reported in literature.[39]

Following a reported procedure,[35] to a suspension of MnO_2 (2.17 g, 25.0 mmol, 5.00 equiv) in acetone (12 mL) was added portionwise potassium (3-(4-chlorophenyl)-3-hydroxyprop-1-yn-1-yl)trifluoroborate (**34**) (1.36 g, 5.00 mmol, 1.00 equiv). The reaction was stirred at rt and followed by ^{19}F NMR spectroscopy. Upon completion (5 h), the mixture was filtered through a pad of celite and eluted with Et_2O (20 mL). All volatiles were removed in vacuo. The obtained solid was dissolved in the minimum amount of hot acetone (10 mL) and precipitation of the desired product was performed by addition of diethyl ether (150 mL). The mixture was cooled down to 0 °C, filtered off, washed with diethyl ether (3 × 20 mL) and dried in vacuo to afford potassium (3-(4-chlorophenyl)-3-oxoprop-1-yn-1-yl)trifluoroborate (**5d**) (599 mg, 2.21 mmol, 44%) as a white solid.

^{1}H NMR (400 MHz, DMSO-d$_6$) δ 8.07 – 8.00 (m, 2H, Ar*H*), 7.68 – 7.60 (m, 2H, Ar*H*). ^{13}C NMR (101 MHz, DMSO-d$_6$) δ 176.9, 138.83, 135.5, 130.7, 129.0. ^{19}F NMR (376 MHz, DMSO-d$_6$) δ -133.2. Spectroscopic data were consistent with values reported in literature.[39]

Potassium ethynyltrifluoroborate (5e):

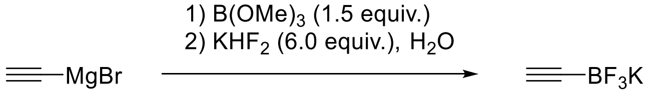

32 5e

Compound **5e** was synthesized following a reported procedure.[40] An oven-dried round-bottom flask (PFA) was evacuated and backfilled with N_2 (3×). Then, a solution of ethynylmagnesium bromide (**32**) (30.0 mL, 15.0 mmol, 0.5 M, 1.0 equiv) in THF and dry THF (30 mL) were added. The solution was cooled to −78 °C and $B(OMe)_3$ (2.5 mL, 22 mmol, 1.5 equiv) was added quickly under N_2. The reaction was stirred 1 h at −78 °C then 1.5 h at −20 °C. A saturated solution of KHF_2 (7.03 g, 90.0 mmol, 6.0 equiv) in water (20 mL + additional 20 mL to rinse the remaining solid) was added. The reaction was stirred at rt open air for 2 h then concentrated in vacuo. The wet solid obtained was further dried by co-evaporation with acetone. To the dry solid was added acetone (≈30 mL) and the resulting mixture was placed on a rotary evaporator and rotated rapidly at atmospheric pressure with the bath set at 45 °C for 15 minutes. The flask

---

[40] P. B. Brady, E. M. Carreira, *Org. Lett.* **2015**, *17*, 3350–3353.

was removed and the mixture carefully filtered taking care to leave the insoluble material in the reaction flask. Acetone was once again added and the process (heating for 15 min then collection of the liquid) was repeated 2 more times. The combined acetone filtrates were concentrated in vacuo to approximately 1/3 of the initial volume. Et_2O (≈30 mL) was added causing a white solid to precipitate. The mixture was cooled to 0 °C for 10 min then filtered. The solid obtained was washed with Et_2O and dried in vacuo to afford potassium ethynyltrifluoroborate (**5e**) (1.17 g, 8.86 mmol, 59%) as a white solid.

^{1}H NMR (400 MHz, acetone-d_6) δ 1.67 (d, *J* = 5.4 Hz, 1H, C≡C*H*). ^{13}C NMR (101 MHz, acetone-d_6) not observed. ^{19}F NMR (376 MHz, acetone-d_6) δ -135.5. Spectroscopic data was consistent with the values reported in the literature.[8]

Potassium trifluoro((3-methoxyphenyl)ethynyl)borate (5f):

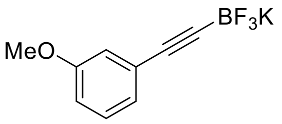

5f

Synthesized following **general procedure B** starting from 1-ethynyl-3-methoxybenzene (1.0 g, 0.97 mL, 7.5 mmol). Potassium trifluoro((3-methoxyphenyl)ethynyl)borate (**5f**) (0.91 g, 3.8 mmol, 51%) was obtained as a white solid.

^{1}H NMR (400 MHz, acetone-d_6) δ 7.15 (t, *J* = 8.0 Hz, 1H, Ar*H*), 6.93 – 6.84 (m, 2H, Ar*H*), 6.78 (ddd, *J* = 8.3, 2.7, 1.0 Hz, 1H, Ar*H*), 3.76 (s, 3H, OC*H₃*). ^{13}C NMR (101 MHz, acetone-d_6) δ 160.3, 129.8, 128.3, 124.5, 117.1, 113.7, 55.4. ^{19}F NMR (377 MHz, acetone-d_6) δ -135.0. Spectroscopic data was consistent with the values reported in the literature.[9]

Potassium trifluoro((4-fluorophenyl)ethynyl)borate (5g):

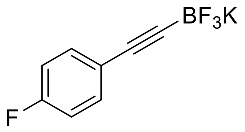

5g

Synthesized following **general procedure B** starting from 1-ethynyl-4-fluorobenzene (0.90 g, 0.86 mL, 7.5 mmol). Potassium trifluoro((4-fluorophenyl)ethynyl)borate (**5g**) (1.11 g, 4.91 mmol, 65%) was obtained as a white solid.

^{1}H NMR (400 MHz, DMSO-d_6) δ 7.36 – 7.28 (m, 2H, Ar*H*), 7.14 – 7.07 (m, 2H, Ar*H*). ^{1}H NMR (400 MHz, acetone-d_6) δ 7.38 – 7.30 (m, 2H, Ar*H*), 7.06 – 6.98 (m, 2H). ^{13}C NMR (101 MHz, acetone-d_6) δ 162.2 (d, *J* = 244.5 Hz), 134.0 (d, *J* = 8.0 Hz), 123.7, 115.8 (d, *J* = 21.9 Hz). ^{19}F NMR (376 MHz, acetone-d_6) δ -115.9, -135.1. Spectroscopic data was consistent with the values reported in the literature.[7]

Potassium ((2-chlorophenyl)ethynyl)trifluoroborate (5h):

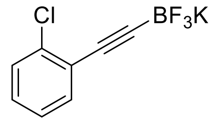

5h

Synthesized following **general procedure B** starting from 1-chloro-2-ethynylbenzene (1.0 g, 0.91 mL, 7.5 mmol). Potassium ((2-chlorophenyl)ethynyl)trifluoroborate (**5h**) (712 mg, 2.94 mmol, 39%) was obtained as a white solid.

Mp (Dec.): 272 °C; ^{1}H NMR (400 MHz, acetone-d_6) δ 7.45 – 7.39 (m, 1H, Ar*H*), 7.39 – 7.33 (m, 1H, Ar*H*), 7.23 – 7.16 (m, 2H, Ar*H*). ^{13}C NMR (101 MHz, acetone-d_6) δ 135.6, 134.4, 129.7, 128.4, 127.3, 127.0. ^{19}F NMR (376 MHz, acetone-d_6) δ -135.1. ^{11}B NMR (128 MHz, acetone-d_6) δ -1.3 (q, *J* = 35.3 Hz). HRMS (ESI/QTOF) m/z: $[M-K]^-$ Calcd for $C_8H_4BClF_3^-$ 203.0052; Found 203.0053.

Potassium trifluoro((4-(methoxycarbonyl)phenyl)ethynyl)borate (5i):

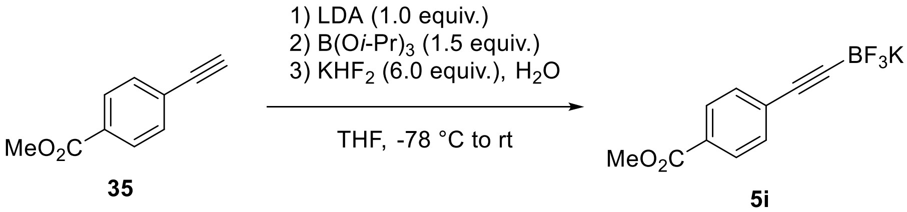

Compound **5i** was synthesized following a reported procedure.[8,41] An oven-dried round-bottom flask (PFA) was evacuated and backfilled with N_2 (3×). Then, freshly distilled diisopropylamine (1.05 mL, 7.50 mmol, 1.0 equiv) and dry THF (15 mL) were added. The mixture was cooled to 0 °C and a solution of *n*-BuLi (3.0 mL, 7.5 mmol, 2.5 M, 1.0 equiv) in hexane was added dropwise under N_2. The reaction was stirred at 0 °C for 0.5 h then cooled to −78 °C. A solution of methyl 4-ethynylbenzoate (**35**) (1.2 g, 7.5 mmol, 1.0 equiv) in dry THF (10 mL) was added dropwise. The reaction was stirred at −78 °C for 0.5 h then B(O*i*-Pr)$_3$ (2.60 mL, 11.3 mmol, 1.5 equiv) was added quickly. The reaction was stirred 10 min at −78 °C then 2 h at rt. The mixture was cooled to 0 °C and a saturated solution of KHF_2 (3.52 g, 45.0 mmol, 6.0 equiv) in water (10 mL + additional 10 mL to rinse the remaining solid) was added. The reaction was stirred at rt open to air for 2 h then concentrated in vacuo. The wet solid obtained was further dried by co-evaporation with acetone. To the dry solid was added acetone (≈30 mL) and the resulting mixture was placed on a rotary evaporator and rotated rapidly at atmospheric pressure with the bath set at 45 °C for 15 minutes. The flask was removed and the mixture carefully filtered taking care to leave the insoluble material in the reaction flask. Acetone was once again added and the process (heating for 15 min then collection of the liquid) was repeated 2 more times. The combined acetone filtrates were concentrated in vacuo to approximately 1/3 of the initial volume. Et_2O (≈40 mL) was added causing a white solid to precipitate. The mixture was cooled to 0 °C for 10 min then filtered. The solid obtained was washed with Et_2O and dried in vacuo

[41] For LDA preparation see: S. Jansone-Popova, J. A. May, *J. Am. Chem. Soc.* **2012**, *134*, 17877–17880.

to afford potassium trifluoro((4-(methoxycarbonyl)phenyl)ethynyl)borate (**5i**) (0.80 g, 3.0 mmol, 40%) as a beige solid.

^{1}H NMR (400 MHz, acetone-d$_6$) δ 7.92 – 7.85 (m, 2H, Ar*H*), 7.45 – 7.38 (m, 2H, Ar*H*), 3.86 (s, 3H, OC*H$_3$*). ^{13}C NMR (101 MHz, acetone-d$_6$) δ 167.0, 132.5, 132.1, 129.9, 128.7, 52.3. ^{19}F NMR (376 MHz, acetone-d$_6$) δ -135.4. Spectroscopic data was consistent with the values reported in the literature.[8]

Potassium trifluoro(mesitylethynyl)borate (5j):

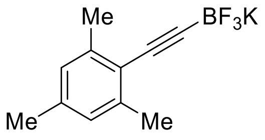

5j

Synthesized following **general procedure B** starting from 2-ethynyl-1,3,5-trimethylbenzene (0.950 g, 1.03 mL, 6.3 mmol). Potassium trifluoro(mesitylethynyl)borate (**5j**) (1.23 g, 4.94 mmol, 78%) was obtained as a white solid.

^{1}H NMR (400 MHz, acetone-d$_6$) δ 6.79 (s, 2H, Ar*H*), 2.34 (s, 6H, C*H$_3$*), 2.20 (s, 3H, C*H$_3$*). ^{13}C NMR (101 MHz, acetone-d$_6$) δ 140.0, 135.9, 127.9, 124.0, 21.3, 21.2. ^{19}F NMR (377 MHz, acetone-d$_6$) δ -134.3. Spectroscopic data was consistent with the values reported in the literature.[8]

Potassium trifluoro(thiophen-3-ylethynyl)borate (5k):

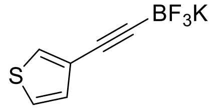

5k

Synthesized following **general procedure B** starting from 3-ethynylthiophene (0.85 g, 0.77 mL, 7.5 mmol). Potassium trifluoro(thiophen-3-ylethynyl)borate (**5k**) (1.29 g, 6.01 mmol, 80%) was obtained as a light brown solid.

Mp (Dec.): 248 °C; ^{1}H NMR (400 MHz, acetone-d$_6$) δ 7.33 (dd, J = 4.9, 3.0 Hz, 1H, Ar*H*), 7.29 (dd, J = 3.0, 1.2 Hz, 1H, Ar*H*), 7.00 (dd, J = 4.9, 1.2 Hz, 1H, Ar*H*). ^{13}C NMR (101 MHz, acetone-d$_6$) δ 131.0, 126.9, 126.4, 125.5. ^{19}F NMR (376 MHz, acetone-d$_6$) δ -135.0. ^{11}B NMR (128 MHz, Acetone) δ -1.3 (q, J = 36.4 Hz). HRMS (ESI/QTOF) m/z: [M-K]$^-$ Calcd for $C_6H_3BF_3S^-$ 175.0006; Found 175.0012.

2-Ethynylbenzofuran (37):

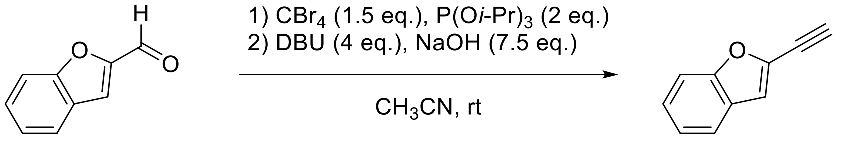

36 **37**

Compound **37** was synthesized following a reported procedure.[42] An oven-dried round-bottom flask charged with CBr_4 (5.0 g, 15 mmol, 1.5 equiv) was evacuated and backfilled with N_2 (3×) then dry CH_3CN (20 mL) and 2-benzofurancarboxaldehyde (**36**) (1.2 mL, 10 mmol, 1.0 equiv) were added. The mixture was cooled to 0 °C and triisopropyl phosphite (4.9 mL, 20 mmol, 2.0 equiv) was added dropwise over 5 min then DBU (6.0 mL, 40 mmol, 4.0 equiv) was added dropwise over 15 minutes. The mixture was stirred at 0 °C for 10 min then at rt for 20 min. Grinded NaOH (3.0 g, 75 mmol, 7.5 equiv) was added and the reaction was stirred at rt for 4 h under N_2 atmosphere. Water (30 mL) and brine (50 mL) were added and the mixture was extracted with 3 × 70 mL of EtOAc. The combined organic layers were washed with brine (100 mL), dried over $MgSO_4$, filtered and concentrated in vacuo. The crude product was loaded on celite and purified by column chromatography (pentane) to afford 2-ethynylbenzofuran (**37**) (516 mg, 3.63 mmol, 36%) as an orange oil.

^{1}H NMR (400 MHz, $CDCl_3$) δ 7.59 – 7.54 (m, 1H, Ar*H*), 7.49 – 7.44 (m, 1H, Ar*H*), 7.36 (td, *J* = 7.8, 1.3 Hz, 1H, Ar*H*), 7.30 – 7.21 (m, 1H, Ar*H*), 7.01 (d, *J* = 0.5 Hz, 1H, OC=C*H*), 3.50 (s, 1H, C≡C*H*). ^{13}C NMR (101 MHz, $CDCl_3$) δ 154.9, 137.8, 127.3, 126.1, 123.5, 121.5, 112.8, 111.5, 83.5, 74.2. Spectroscopic data was consistent with the values reported in the literature.[42]

Potassium (benzofuran-2-ylethynyl)trifluoroborate (5l):

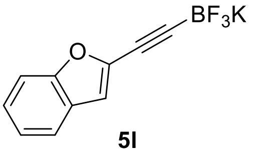

5l

Synthesized following **general procedure B** starting from 2-ethynylbenzofuran (**37**) (500 mg, 3.52 mmol). After recrystallization (acetone/Et_2O) potassium (benzofuran-2-ylethynyl)trifluoroborate (**5l**) (568 mg, 2.29 mmol, 65%) was obtained as a white solid.

^{1}H NMR (400 MHz, DMSO-d_6) δ 7.59 – 7.54 (m, 1H, Ar*H*), 7.51 – 7.45 (m, 1H, Ar*H*), 7.30 (dd, *J* = 6.0, 1.4 Hz, 1H, Ar*H*), 7.22 (td, *J* = 7.6, 1.0 Hz, 1H, Ar*H*), 6.92 (s, 1H, OC=C*H*). ^{13}C NMR (101 MHz, DMSO-d_6) δ 153.5, 140.4, 127.7, 124.8, 123.1, 120.9, 110.7, 108.6. ^{19}F NMR (377 MHz, DMSO-d_6) δ -132.7. Spectroscopic data was consistent with the values reported in the literature.[43]

---

[42] Y. Thummala, G. V. Karunakar, V. R. Doddi, *Adv. Synth. Catal.* **2019**, *361*, 611–616.
[43] J.-F. Wang, X. Meng, C.-H. Zhang, C.-M. Yu, B. Mao, *Org. Lett.* **2020**, *22*, 7427–7432.

Potassium trifluoro(prop-1-yn-1-yl)borate (5m):

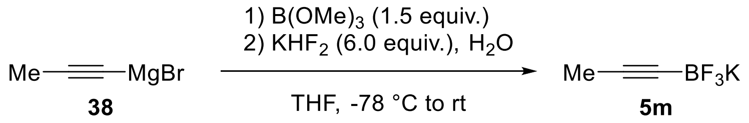

Compound **5m** was synthesized following a reported procedure.[8] An oven-dried round-bottom flask (PFA) was evacuated and backfilled with N_2 (3×). Then, a solution of 1-propynylmagnesium bromide (**38**) (15 mL, 7.5 mmol, 0.5 M, 1.0 equiv) in THF and dry THF (15 mL) were added. The solution was cooled to −78 °C and $B(OMe)_3$ (1.25 mL, 11.3 mmol, 1.5 equiv) was added quickly under N_2. The reaction was stirred 1 h at −78 °C then 1.5 h at −20 °C. A saturated solution of KHF_2 (3.5 g, 45 mmol, 6.0 equiv) in water (10 mL + additional 10 mL to rinse the remaining solid) was added. The reaction was stirred at rt open air for 2 h then concentrated in vacuo. The wet solid obtained was further dried by co-evaporation with acetone. To the dry solid was added acetone (≈30 mL) and the resulting mixture was placed on a rotary evaporator and rotated rapidly at atmospheric pressure with the bath set at 45 °C for 15 minutes. The flask was removed and the mixture carefully filtered taking care to leave the insoluble material in the reaction flask. Acetone was once again added and the process (heating for 15 min then collection of the liquid) was repeated 2 more times. The combined acetone filtrates were concentrated in vacuo to approximately 1/3 of the initial volume. Et_2O (≈30 mL) was added causing a white solid to precipitate. The mixture was cooled to 0 °C for 10 min then filtered. The solid obtained was washed with Et_2O and dried in vacuo to afford potassium trifluoro(prop-1-yn-1-yl)borate (**5m**) (0.95 g, 6.5 mmol, 87%) as a white solid.

^{1}H NMR (400 MHz, acetone-d$_6$) δ 1.64 – 1.58 (m, 3H, C*H$_3$*). ^{13}C NMR (101 MHz, acetone-d$_6$) δ 4.0. ^{19}F NMR (376 MHz, acetone-d$_6$) δ -134.7. Spectroscopic data was consistent with the values reported in the literature.[8]

Potassium (cyclopropylethynyl)trifluoroborate (5n):

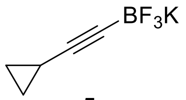

5n

Synthesized following **general procedure B** starting from ethynylcyclopropane (0.50 g, 0.64 mL, 7.5 mmol). Potassium (cyclopropylethynyl)trifluoroborate (**5n**) (0.86 g, 5.0 mmol, 67%) was obtained as a white solid.

^{1}H NMR (400 MHz, DMSO-d$_6$) δ 1.12 – 1.01 (m, 1H, C*H*), 0.61 – 0.54 (m, 2H, C*H$_2$*), 0.42 – 0.36 (m, 2H, C*H$_2$*). ^{13}C NMR (101 MHz, DMSO-d$_6$) δ 7.4, 0.1. ^{19}F NMR (377 MHz, DMSO-d$_6$) δ -131.1. Spectroscopic data was consistent with the values reported in the literature.[8]

Potassium (5-chloropent-1-yn-1-yl)trifluoroborate (5o):

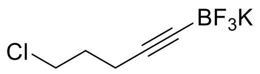

5o

Synthesized following **general procedure B** starting from 5-chloropent-1-yne (0.77 g, 0.80 mL, 7.5 mmol). Potassium (5-chloropent-1-yn-1-yl)trifluoroborate (**5o**) (1.28 g, 6.14 mmol, 82%) was obtained as a white solid.

^{1}H NMR (400 MHz, acetone-d$_6$) δ 3.70 (t, *J* = 6.6 Hz, 2H, C*H*$_2$Cl), 2.24 – 2.17 (m, 2H, C≡C-C*H*$_2$), 1.85 (p, *J* = 6.7 Hz, 2H, CH$_2$C*H*$_2$CH$_2$). ^{13}C NMR (101 MHz, acetone-d$_6$) δ 44.9, 33.1, 17.3. ^{19}F NMR (376 MHz, acetone-d$_6$) δ -134.6. Spectroscopic data was consistent with the values reported in the literature.[34]

7.4 Scope of the azido-alkynylation previously reported by our group

General Note: We observed that the homopropargylic azides synthetized in this work tend to slowly decompose even when stored in the fridge at 4 °C

General procedure C:

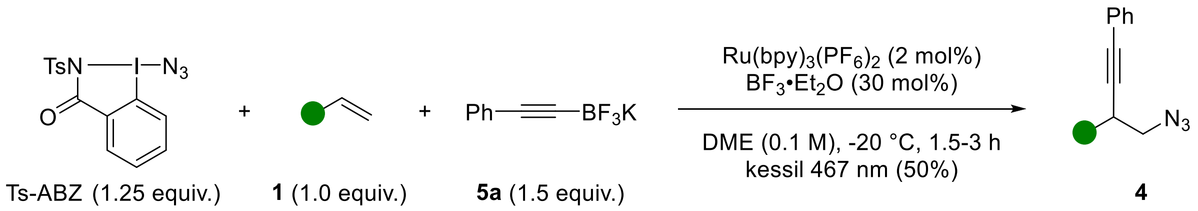

An oven-dried test tube charged with Ru(bpy)$_3$(PF$_6$)$_2$ (5.2 mg, 6.0 µmol, 0.02 equiv), Ts-ABZ (166 mg, 0.375 mmol, 1.25 equiv), potassium trifluoro(phenylethynyl)borate (**5a**) (94 mg, 0.45 mmol, 1.50 equiv) and alkene (**1**) (if solid, 0.30 mmol, 1.00 equiv) was evacuated and backfilled with N$_2$ (3×). Dry degassed DME (2.7 mL) and alkene (**1**) (if liquid, 0.30 mmol, 1.00 equiv) were added and the mixture was cooled to −20 °C. Then, a stock solution of BF$_3$•Et$_2$O (11 µL, 90 µmol, 0.30 equiv) in dry degassed DME (0.34 mL) was added and the reaction was stirred under blue LEDs irradiation (1 × Kessil 467 nm, 50% intensity 22 W) at −20 °C until full conversion was observed (1.5-3 h). The reaction mixture was filtered through a short plug of silica and eluted with DCM or EtOAc then concentrated in vacuo. The crude product was purified by column chromatography to afford **4**.

Note: Commercially available liquid alkenes were eluted through a short plug of basic Al$_2$O$_3$ before use. DME was sparged with argon for 0.5 h before use. Cooling was performed using a Dewar filled with a mixture of ice and salt. We did not observe significant rise in temperature after 1.5 h (which is enough in most cases to reach full conversion). In the case of longer reactions, the cold bath was replaced with a new one after 1.5 h. For further details on the photochemistry set-up see Figure S1.

General procedure D:

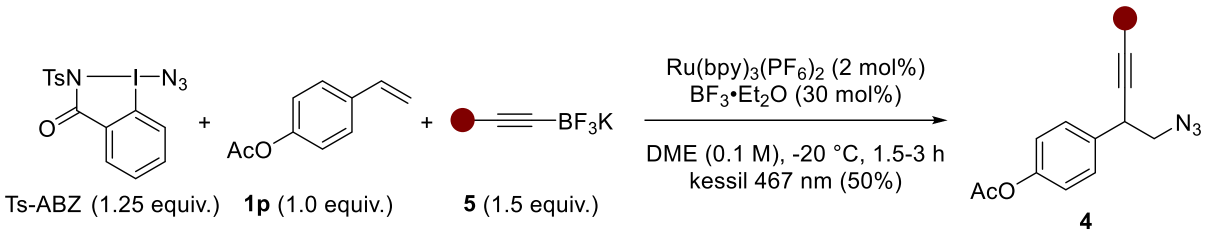

An oven-dried test tube charged with $Ru(bpy)_3(PF_6)_2$ (5.2 mg, 6.0 µmol, 0.02 equiv), Ts-ABZ (166 mg, 0.375 mmol, 1.25 equiv) and potassium trifluoroborate **5** (0.45 mmol, 1.50 equiv) was evacuated and backfilled with N_2 (3×). Dry degassed DME (2.7 mL) and 4-acetoxystyrene (**1p**) (46 µL, 0.30 mmol, 1.00 equiv) were added and the mixture was cooled to −20 °C. Then, a stock solution of BF_3•Et_2O (11 µL, 90 µmol, 0.30 equiv) in dry degassed DME (0.34 mL) was added and the reaction was stirred under blue LEDs irradiation (1 × Kessil 467 nm 50% intensity, 22 W) at −20 °C for 1.5-3 h. The reaction mixture was filtered through a short plug of silica and eluted with DCM then concentrated in vacuo. The crude product was purified by column chromatography to afford **4**.

Note: 4-Acetoxystyrene (**1p**) was eluted through a short plug of basic Al_2O_3 before use. DME was sparged with argon for 0.5 h before use. Cooling was performed using a Dewar filled with a mixture of ice and salt. We did not observe significant rise in temperature after 1.5 h (which is enough in most cases to reach full conversion). In the case of longer reaction, the cold bath was replaced with a new one after 1.5 h. For further details on the photochemistry set-up see Figure S1.

(4-Azidobut-1-yne-1,3-diyl)dibenzene (4a):

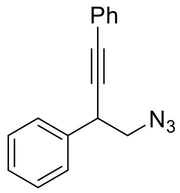

4a

Synthesized following **general procedure C** starting from styrene (**1a**) (35 µL, 0.30 mmol, 1.00 equiv). The reaction was carried out for 1.5 h, DCM was used for the silica plug. The crude product was purified by column chromatography (pentane/toluene, 85:15) to afford (4-azidobut-1-yne-1,3-diyl)dibenzene (**4a**) (55 mg, 0.22 mmol, 73%) as a yellow oil.

R_f (pentane/toluene, 7:3): 0.45; ^{1}H NMR (400 MHz, $CDCl_3$) δ 7.52 – 7.45 (m, 4H, Ar*H*), 7.42 – 7.36 (m, 2H, Ar*H*), 7.35 – 7.30 (m, 4H, Ar*H*), 4.13 (t, *J* = 6.8 Hz, 1H, C*H*C≡C), 3.63 (dd, *J* = 12.0, 7.6 Hz, 1H, C*H*$_2$N$_3$), 3.53 (dd, *J* = 12.0, 6.2 Hz, 1H, C*H*$_2$N$_3$). ^{13}C NMR (101 MHz, $CDCl_3$) δ 138.1, 131.7, 128.8, 128.3, 128.3, 127.9, 127.8, 123.0, 87.9, 84.9, 57.3, 39.6. HRMS (APPI/LTQ-Orbitrap) m/z: $[M-N_2+H]^+$ Calcd for $C_{16}H_{14}N^+$ 220.1121; Found 220.1120.

1-(1-Azido-4-phenylbut-3-yn-2-yl)-4-(*tert*-butyl)benzene (4b):

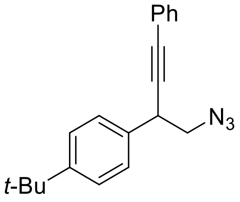

4b

Synthesized following **general procedure C** starting from 4-*tert*-butylstyrene (55 µL, 0.30 mmol, 1.00 equiv). The reaction was carried out for 1.5 h, DCM was used for the silica plug. The crude product was purified by column chromatography (pentane/toluene, 95:5 to 90:10) to afford 1-(1-azido-4-phenylbut-3-yn-2-yl)-4-(tert-butyl)benzene (**4b**) (71 mg, 0.24 mmol, 78%) as a colorless oil.

R_f (pentane/toluene, 85 :15) : 0.27; ^1H NMR (400 MHz, $CDCl_3$) δ 7.54 – 7.48 (m, 2H, Ar*H*), 7.45 – 7.39 (m, 4H, Ar*H*), 7.36 – 7.30 (m, 3H, Ar*H*), 4.12 (dd, *J* = 7.7, 6.1 Hz, 1H, C*H*C≡C), 3.63 (dd, *J* = 12.0, 7.8 Hz, 1H, CH_2N_3), 3.53 (dd, *J* = 12.0, 6.1 Hz, 1H, CH_2N_3), 1.35 (s, 9H, *t*-Bu). ^{13}C NMR (101 MHz, $CDCl_3$) δ 150.8, 135.2, 131.8, 128.4, 128.3, 127.6, 125.9, 123.2, 88.3, 84.8, 57.4, 39.3, 34.7, 31.5. HRMS (ESI/QTOF) m/z: $[M+H]^+$ Calcd for $C_{20}H_{22}N_3^+$ 304.1808; Found 304.1804.

2-(1-Azido-4-phenylbut-3-yn-2-yl)-1,3,5-trimethylbenzene (4c):

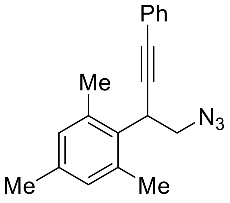

4c

Synthesized following **general procedure C** starting from 2,4,6-trimethylstyrene (48 µL, 0.30 mmol, 1.00 equiv). The reaction was carried out for 1.5 h, DCM was used for the silica plug. The crude product was purified by column chromatography (pentane/Et_2O, 97.5:2.5) to afford 2-(1-azido-4-phenylbut-3-yn-2-yl)-1,3,5-trimethylbenzene (**4c**) (68 mg, 0.24 mmol, 78%) as a yellow oil.

R_f (pentane/Et_2O, 95:5): 0.5; ^1H NMR (400 MHz, $CDCl_3$) δ 7.48 – 7.43 (m, 2H, Ar*H*), 7.35 – 7.30 (m, 3H, Ar*H*), 6.91 (s, 2H, Ar*H*), 4.61 (dd, *J* = 8.6, 7.0 Hz, 1H, C*H*C≡C), 3.83 (dd, *J* = 12.0, 8.8 Hz, 1H, CH_2N_3), 3.49 (dd, *J* = 12.0, 6.9 Hz, 1H, CH_2N_3), 2.54 (s, 6H, CH_3), 2.30 (s, 3H, CH_3). ^{13}C NMR (101 MHz, $CDCl_3$) δ 137.2, 136.8, 131.6, 131.0, 130.4, 128.4, 128.1, 123.4, 88.5, 84.1, 53.9, 34.1, 21.0, 20.9. HRMS (APPI/LTQ-Orbitrap) m/z: $[M-N_2+H]^+$ Calcd for $C_{19}H_{20}N^+$ 262.1590; Found 262.1590.

1-(1-Azido-4-phenylbut-3-yn-2-yl)-4-methoxybenzene (4d):

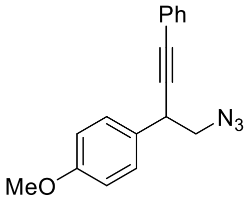

4d

Synthesized following **general procedure C** starting from 4-vinylanisole (40 µL, 0.30 mmol, 1.00 equiv). The reaction was carried out for 1.5 h, DCM was used for the silica plug. The crude product was purified by column chromatography (pentane/Et_2O, 97.5:2.5 to 95:5) to afford 1-(1-azido-4-phenylbut-3-yn-2-yl)-4-methoxybenzene (**4d**) (70 mg, 0.25 mmol, 84%) as a yellow oil.

R_f (pentane/Et_2O, 9:1): 0.35; ^1H NMR (400 MHz, $CDCl_3$) δ 7.51 – 7.46 (m, 2H, Ar*H*), 7.41 – 7.36 (m, 2H, Ar*H*), 7.34 – 7.30 (m, 3H, Ar*H*), 6.95 – 6.89 (m, 2H, Ar*H*), 4.10 – 4.05 (m, 1H, C*H*C≡C), 3.82 (s, 3H, OC*H₃*), 3.60 (dd, *J* = 12.0, 7.6 Hz, 1H, CH_2N_3), 3.50 (dd, *J* = 12.0, 6.3 Hz, 1H, CH_2N_3). ^{13}C NMR (101 MHz, $CDCl_3$) δ 159.3, 131.8, 130.3, 129.0, 128.4, 128.4, 123.1, 114.3, 88.4, 84.8, 57.5, 55.5, 38.9. HRMS (APPI/LTQ-Orbitrap) m/z: $[M-N_2+H]^+$ Calcd for $C_{17}H_{16}NO^+$ 250.1226; Found 250.1226.

4-(1-Azido-4-phenylbut-3-yn-2-yl)phenyl acetate (4e):

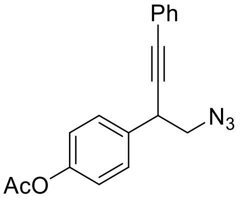

4e

Synthesized following **general procedure C** starting from 4-vinylphenyl acetate (**1p**) (46 µL, 0.30 mmol, 1.00 equiv). The reaction was carried out for 1.5 h, DCM was used for the silica plug. The crude product was purified by column chromatography (pentane/Et_2O, 90:10 to 85:15) to afford 4-(1-azido-4-phenylbut-3-yn-2-yl)phenyl acetate (**4e**) (71 mg, 0.23 mmol, 78%) as a yellow oil.

R_f (pentane/Et_2O, 8:2): 0.33; ^1H NMR (400 MHz, $CDCl_3$) δ 7.52 – 7.44 (m, 4H, Ar*H*), 7.36 – 7.30 (m, 3H, Ar*H*), 7.14 – 7.08 (m, 2H, Ar*H*), 4.13 (dd, *J* = 7.5, 6.2 Hz, 1H, C*H*C≡C), 3.62 (dd, *J* = 12.0, 7.6 Hz, 1H, CH_2N_3), 3.52 (dd, *J* = 12.0, 6.1 Hz, 1H, CH_2N_3), 2.31 (s, 3H, CH_3). ^{13}C NMR (101 MHz, $CDCl_3$) δ 169.5, 150.2, 135.8, 131.8, 129.0, 128.5, 128.4, 122.9, 122.0, 87.7, 85.2, 57.3, 39.2, 21.2. HRMS (ESI/QTOF) m/z: $[M+Na]^+$ Calcd for $C_{18}H_{15}N_3NaO_2^+$ 328.1056; Found 328.1056.

***N*-(4-(1-Azido-4-phenylbut-3-yn-2-yl)phenyl)acetamide (4f):**

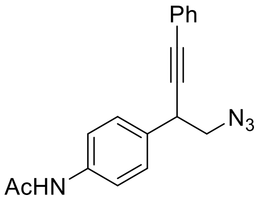

4f

Synthesized following **general procedure C** starting from *N*-(4-vinylphenyl)acetamide (**1s**) (52 mg, 0.30 mmol, 1.00 equiv). The reaction was carried out for 1.5 h, EtOAc was used for the silica plug. The crude product was dissolved in DCM (15 mL), the solution was washed with 3 × 10 mL of a sat. sol. of $NaHCO_3$. The organic layer was dried over $MgSO_4$, filtered and concentrated in vacuo. The crude product was purified by column chromatography (pentane/EtOAc, 6:4 to 5:5) to afford *N*-(4-(1-azido-4-phenylbut-3-yn-2-yl)phenyl)acetamide (**4f**) (62 mg, 0.20 mmol, 68%) as a yellow solid.

R_f (pentane/EtOAc, 4:6): 0.45; Mp (Dec.): 139 °C; ^{1}H NMR (400 MHz, $CDCl_3$) δ 7.55 – 7.44 (m, 4H, Ar*H*), 7.41 (d, *J* = 8.4 Hz, 2H, Ar*H*), 7.35 – 7.29 (m, 3H, Ar*H*), 7.22 (bs, 1H, N*H*Ac), 4.09 (t, *J* = 6.8 Hz, 1H, C*H*C≡C), 3.60 (dd, *J* = 12.0, 7.5 Hz, 1H, CH_2N_3), 3.50 (dd, *J* = 12.0, 6.2 Hz, 1H, CH_2N_3), 2.18 (s, 3H, CH_3). ^{13}C NMR (101 MHz, $CDCl_3$) δ[44] 168.4, 137.5, 134.1, 131.8, 128.6, 128.4, 123.0, 120.3, 87.9, 85.1, 57.4, 39.2, 24.8. HRMS (ESI/QTOF) m/z: $[M+Na]^+$ Calcd for $C_{18}H_{16}N_4NaO^+$ 327.1216; Found 327.1212.

1-(1-Azido-4-phenylbut-3-yn-2-yl)-4-bromobenzene (4g):

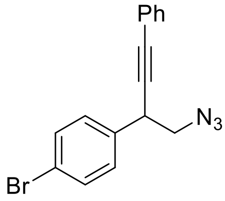

4g

Synthesized following **general procedure C** starting from 4-bromostyrene (41 µL, 0.30 mmol, 1.00 equiv). The reaction was carried out for 1.5 h, DCM was used for the silica plug. The crude product was purified by column chromatography (pentane/toluene, 90:10 to 85:15) to afford 1-(1-azido-4-phenylbut-3-yn-2-yl)-4-bromobenzene (**4g**) (69 mg, 0.21 mmol, 71%) as a yellow oil.

R_f (pentane/toluene, 7:3): 0.48; ^{1}H NMR (400 MHz, $CDCl_3$) δ 7.54 – 7.46 (m, 4H, Ar*H*), 7.38 – 7.30 (m, 5H, Ar*H*), 4.08 (t, *J* = 6.8 Hz, 1H, C*H*C≡C), 3.62 (dd, *J* = 12.0, 7.3 Hz, 1H, CH_2N_3), 3.52 (dd, *J* = 12.0, 6.3 Hz, 1H, CH_2N_3). ^{13}C NMR (101 MHz, $CDCl_3$) δ 137.3, 132.0, 131.8,

[44] One aromatic carbon was not resolved.

129.7, 128.6, 128.5, 122.8, 121.8, 87.3, 85.4, 57.1, 39.2. HRMS (Sicrit plasma/LTQ-Orbitrap) m/z: $[M+H]^+$ Calcd for $C_{16}H_{13}BrN_3^+$ 326.0287; Found 326.0286.

Methyl 4-(1-azido-4-phenylbut-3-yn-2-yl)benzoate (4h):

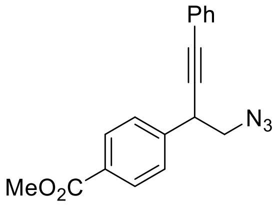

4h

Synthesized following **general procedure C** starting from methyl 4-vinylbenzoate (49 mg, 0.30 mmol, 1.00 equiv). The reaction was carried out for 3 h, DCM was used for the silica plug. The crude product was purified by column chromatography (pentane/Et_2O, 9:1) to afford methyl 4-(1-azido-4-phenylbut-3-yn-2-yl)benzoate (**4h**) (45 mg, 0.18 mmol, 49%) as a yellow oil.

R_f (pentane/Et_2O, 8:2): 0.45; ^1H NMR (400 MHz, $CDCl_3$) δ 8.08 – 8.03 (m, 2H, Ar*H*), 7.57 – 7.52 (m, 2H, Ar*H*), 7.51 – 7.46 (m, 2H, Ar*H*), 7.36 – 7.30 (m, 3H, Ar*H*), 4.18 (t, *J* = 6.7 Hz, 1H, C*H*C≡C), 3.93 (s, 3H, C*H₃*), 3.65 (dd, *J* = 12.0, 7.3 Hz, 1H, CH_2N_3), 3.56 (dd, *J* = 12.0, 6.3 Hz, 1H, CH_2N_3). ^{13}C NMR (101 MHz, $CDCl_3$) δ 166.8, 143.4, 131.8, 130.2, 129.8, 128.6, 128.5, 128.1, 122.8, 87.1, 85.5, 57.1, 52.3, 39.7. HRMS (ESI/QTOF) m/z: $[M+Na]^+$ Calcd for $C_{18}H_{15}N_3NaO_2^+$ 328.1056; Found 328.1061.

2-(1-Azido-4-phenylbut-3-yn-2-yl)thiophene (4i):

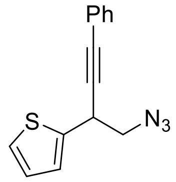

4i

Synthesized following **general procedure C** starting from a solution of 2-vinylthiophene (35 mg, 0.30 mmol, 1.00 equiv) in dry degassed DME (0.5 mL). The reaction was carried out for 1.5 h, DCM was used for the silica plug. The crude product was purified by column chromatography (pentane/toluene, 90:10 to 85:15) to afford 2-(1-azido-4-phenylbut-3-yn-2-yl)thiophene (**4i**) (47 mg, 0.19 mmol, 62%) as a yellow oil.

R_f (pentane/toluene, 7:3): 0.55; ^1H NMR (400 MHz, $CDCl_3$) δ 7.53 – 7.47 (m, 2H, Ar*H*), 7.37 – 7.31 (m, 3H, Ar*H*), 7.28 (dd, *J* = 5.1, 1.2 Hz, 1H, Ar*H*), 7.16 – 7.11 (m, 1H, Ar*H*), 7.01 (dd, *J* = 5.1, 3.5 Hz, 1H, Ar*H*), 4.39 (t, *J* = 6.6 Hz, 1H, C*H*C≡C), 3.70 (dd, *J* = 12.0, 7.2 Hz, 1H, CH_2N_3), 3.61 (dd, *J* = 12.0, 6.2 Hz, 1H, CH_2N_3). ^{13}C NMR (101 MHz, $CDCl_3$) δ 141.3, 131.9, 128.6, 128.4, 127.1, 125.8, 125.2, 122.7, 87.4, 84.7, 57.4, 35.0. HRMS (APPI/LTQ-Orbitrap) m/z: $[M-N_2+H]^+$ Calcd for $C_{14}H_{12}NS^+$ 226.0685; Found 226.0687.

2-(1-Azido-4-phenylbut-3-yn-2-yl)-5-bromofuran (4j):

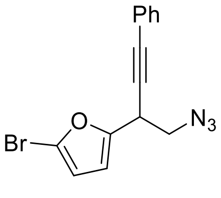

4j

Synthesized following **general procedure C** starting from a solution of 2-bromo-5-vinylfuran (**1v**) (52 mg, 0.30 mmol, 1.00 equiv) in dry degassed DME (0.5 mL). The reaction was carried out for 1.5 h, DCM was used for the silica plug. The crude product was purified by column chromatography (pentane/toluene, 9:1) to afford 2-(1-azido-4-phenylbut-3-yn-2-yl)-5-bromofuran (**4j**) (52 mg, 0.16 mmol, 54%) as an orange oil.

R_f (pentane/toluene, 8:2): 0.43; ^{1}H NMR (400 MHz, $CDCl_3$) δ 7.50 – 7.45 (m, 2H, Ar*H*), 7.36 – 7.30 (m, 3H, Ar*H*), 6.40 (dd, *J* = 3.3, 0.9 Hz, 1H, Ar*H*), 6.30 (d, *J* = 3.3 Hz, 1H, Ar*H*), 4.20 (t, *J* = 6.0 Hz, 1H, C*H*C≡C), 3.68 (dd, *J* = 6.2, 2.5 Hz, 2H, C$H_2$$N_3$). ^{13}C NMR (101 MHz, $CDCl_3$) δ 152.8, 131.9, 128.7, 128.5, 122.5, 121.5, 112.4, 110.7, 84.7, 84.5, 54.2, 33.8. HRMS (APPI/LTQ-Orbitrap) m/z: $[M-N_2+H]^+$ Calcd for $C_{14}H_{11}BrNO^+$ 288.0019; Found 288.0025.

2-Azido-1-(phenylethynyl)-1,2,3,4-tetrahydronaphthalene (4k/4k'):

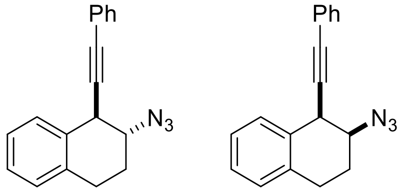

4k **4k'**

Synthesized following **general procedure C** starting from 1,2-dihydronaphthalene (39 µL, 0.30 mmol, 1.00 equiv). The reaction was carried out for 1.5 h, DCM was used for the silica plug. Crude ^{1}H NMR of the mixture showed a diastereomeric ratio of 3.8:1 (*trans*:*cis*) using peaks at 2.08 and 1.89 ppm. The crude product was purified by column chromatography (pentane/toluene, 9:1 to 8:2). Relative configuration of the diastereoisomers were determined by analogy to **4ad/4ad'** where the major product is *trans*.

trans diastereoisomer (major): 1,2-*trans*-2-azido-1-(phenylethynyl)-1,2,3,4-tetrahydronaphthalene (**4k**) (50 mg, 0.18 mmol, 61%) yellow oil.

R_f (pentane/toluene, 8:2): 0.42; ^{1}H NMR (400 MHz, $CDCl_3$) δ 7.62 – 7.58 (m, 1H, Ar*H*), 7.50 – 7.44 (m, 2H, Ar*H*), 7.34 – 7.29 (m, 3H, Ar*H*), 7.25 – 7.19 (m, 2H, Ar*H*), 7.12 (d, *J* = 6.8 Hz, 1H, Ar*H*), 4.04 (d, *J* = 8.2 Hz, 1H, C*H*C≡C), 4.01 – 3.95 (m, 1H, C*H*N_3), 3.00 – 2.93 (m, 2H, ArCH_2), 2.36 – 2.27 (m, 1H, ArCH_2CH_2), 1.96 – 1.83 (m, 1H, ArCH_2CH_2). ^{13}C NMR (101 MHz, $CDCl_3$) δ 134.6, 133.7, 131.9, 129.3, 128.8, 128.4, 128.3, 127.3, 126.7, 123.2, 89.5, 83.8, 62.5, 39.2, 27.4, 27.2. HRMS (APPI/LTQ-Orbitrap) m/z: $[M-N_2+H]^+$ Calcd for $C_{18}H_{16}N^+$ 246.1277; Found 246.1269.

cis diastereoisomer (minor): 1,2-*cis*-2-azido-1-(phenylethynyl)-1,2,3,4-tetrahydronaphthalene (**4k'**) (8 mg, 0.03 mmol, 10%) orange oil.

R_f (pentane/toluene, 8:2): 0.32; ^{1}H NMR (400 MHz, $CDCl_3$) δ 7.51 – 7.43 (m, 3H, Ar*H*), 7.31 – 7.27 (m, 3H, Ar*H*), 7.24 – 7.18 (m, 2H, Ar*H*), 7.15 – 7.10 (m, 1H, Ar*H*), 4.27 (d, *J* = 4.3 Hz, 1H, C*H*C≡C), 4.01 (ddd, *J* = 8.5, 4.3, 2.8 Hz, 1H, C*H*N_3), 3.08 (dt, *J* = 17.2, 6.5 Hz, 1H, ArCH_2), 2.87 (dt, *J* = 17.2, 6.9 Hz, 1H, ArCH_2), 2.37 – 2.27 (m, 1H, ArCH_2CH_2), 2.13 – 2.04 (m, 1H, ArCH_2CH_2). ^{13}C NMR (101 MHz, $CDCl_3$) δ 134.5, 133.9, 131.9, 129.6, 129.0, 128.3, 128.2, 127.5, 126.6, 123.3, 88.2, 85.0, 59.6, 38.3, 26.5, 25.6. HRMS (APPI/LTQ-Orbitrap) m/z: $[M-N_2+H]^+$ Calcd for $C_{18}H_{16}N^+$ 246.1277; Found 246.1275.

(3-(Azidomethyl)penta-1,4-diyne-1,5-diyl)dibenzene (4l):

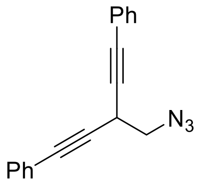

4l

Synthesized following **general procedure C** starting from a solution of but-3-en-1-yn-1-ylbenzene (**1q**) (39 mg, 0.30 mmol, 1.00 equiv) in dry degassed DME (0.5 mL). The reaction was carried out for 1.5 h, DCM was used for the silica plug. The crude product was purified by column chromatography (pentane/toluene, 85:15) to afford (3-(azidomethyl)penta-1,4-diyne-1,5-diyl)dibenzene (**4l**) (36 mg, 0.13 mmol, 44%) as a yellow oil.

R_f (pentane/toluene, 8:2): 0.32; ^{1}H NMR (400 MHz, $CDCl_3$) δ 7.52 – 7.44 (m, 4H, Ar*H*), 7.37 – 7.29 (m, 6H, Ar*H*), 4.09 (t, *J* = 6.7 Hz, 1H, C*H*C≡C), 3.64 (d, *J* = 6.7 Hz, 2H, CH_2N_3). ^{13}C NMR (101 MHz, $CDCl_3$) δ 132.0, 128.7, 128.4, 122.6, 84.4, 83.2, 55.1, 26.8. HRMS (APPI/LTQ-Orbitrap) m/z: $[M-N_2+H]^+$ Calcd for $C_{18}H_{14}N^+$ 244.1121; Found 244.1121.

(4-Azido-3-butoxybut-1-yn-1-yl)benzene (4m):

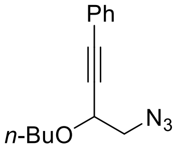

4m

Synthesized following **general procedure C** starting from vinyl butyl ether (39 µL, 0.30 mmol, 1.00 equiv). The reaction was carried out for 1.5 h, DCM was used for the silica plug. The crude product was purified by column chromatography (pentane/Et_2O, 99:1 to 98:2) to afford (4-azido-3-butoxybut-1-yn-1-yl)benzene (**4m**) (36 mg, 0.15 mmol, 49%) as a slightly yellow oil.

R_f (pentane/Et_2O, 95:5): 0.55; ^{1}H NMR (400 MHz, $CDCl_3$) δ 7.47 – 7.43 (m, 2H, Ar*H*), 7.35 – 7.30 (m, 3H, Ar*H*), 4.43 (dd, *J* = 7.6, 4.0 Hz, 1H, C*H*C≡C), 3.86 (dt, *J* = 9.1, 6.5 Hz, 1H, CH_2O), 3.61 – 3.47 (m, 2H, CH_2O + CH_2N_3), 3.38 (dd, *J* = 12.8, 4.0 Hz, 1H, CH_2N_3), 1.68 – 1.60 (m,

2H, CH_2CH$_2$O), 1.49 – 1.39 (m, 2H, CH_2CH$_3$), 0.95 (t, J = 7.4 Hz, 3H, CH$_2$CH_3). ^{13}C NMR (101 MHz, CDCl$_3$) δ 131.9, 128.9, 128.5, 122.3, 87.1, 85.3, 69.9, 69.5, 54.7, 31.8, 19.4, 14.0. HRMS (ESI/QTOF) m/z: [M+Na]$^+$ Calcd for $C_{14}H_{17}N_3NaO^+$ 266.1264; Found 266.1260.

4-(1-Azido-4-(3-methoxyphenyl)but-3-yn-2-yl)phenyl acetate (4n):

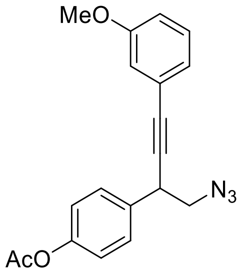

4n

Synthesized following **general procedure D** starting from potassium trifluoro((3-methoxyphenyl)ethynyl)borate (**5g**) (107 mg, 0.450 mmol, 1.50 equiv). The reaction was carried out for 1.5 h, DCM was used for the silica plug. The crude product was purified by column chromatography (pentane/Et$_2$O, 85:15) to afford 4-(1-azido-4-(3-methoxyphenyl)but-3-yn-2-yl)phenyl acetate (**4n**) (60 mg, 0.18 mmol, 60%) as a yellow oil.

R_f (pentane/Et$_2$O, 8:2): 0.19; ^{1}H NMR (400 MHz, CDCl$_3$) δ 7.50 – 7.45 (m, 2H, ArH), 7.27 – 7.20 (m, 1H, ArH), 7.14 – 7.05 (m, 3H, ArH), 7.01 (dd, J = 2.4, 1.4 Hz, 1H, ArH), 6.89 (ddd, J = 8.3, 2.6, 0.8 Hz, 1H, ArH), 4.16 – 4.09 (m, 1H, CHC≡C), 3.81 (s, 3H, OCH_3), 3.62 (dd, J = 12.0, 7.7 Hz, 1H, CH_2N$_3$), 3.52 (dd, J = 12.0, 6.1 Hz, 1H, CH_2N$_3$), 2.31 (s, 3H, CH_3). ^{13}C NMR (101 MHz, CDCl$_3$) δ 169.6, 159.4, 150.3, 135.7, 129.5, 129.1, 124.3, 123.9, 122.1, 116.6, 115.2, 87.6, 85.1, 57.3, 55.4, 39.2, 21.3. HRMS (ESI/QTOF) m/z: [M+Na]$^+$ Calcd for $C_{19}H_{17}N_3NaO_3^+$ 358.1162; Found 358.1161.

4-(1-Azido-4-(4-fluorophenyl)but-3-yn-2-yl)phenyl acetate (4o):

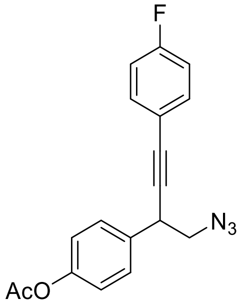

4o

Synthesized following **general procedure D** starting from potassium trifluoro((4-fluorophenyl)ethynyl)borate (**5h**) (102 mg, 0.450 mmol, 1.50 equiv). The reaction was carried out for 1.5 h, DCM was used for the silica plug. The crude product was purified by column chromatography (pentane/Et$_2$O, 90:10 to 85:15) to afford 4-(1-azido-4-(4-fluorophenyl)but-3-yn-2-yl)phenyl acetate (**4o**) (65 mg, 0.20 mmol, 67%) as a yellow oil.

R_f (pentane/Et_2O, 8:2): 0.3; ^{1}H NMR (400 MHz, $CDCl_3$) δ 7.45 (dd, *J* = 8.6, 4.7 Hz, 4H, Ar*H*), 7.14 – 7.08 (m, 2H, Ar*H*), 7.06 – 6.97 (m, 2H, Ar*H*), 4.14 – 4.08 (m, 1H, C*H*C≡C), 3.60 (dd, *J* = 12.0, 7.7 Hz, 1H, CH_2N_3), 3.51 (dd, *J* = 12.0, 6.0 Hz, 1H, CH_2N_3), 2.31 (s, 3H, CH_3). ^{13}C NMR (101 MHz, $CDCl_3$) δ 169.5, 162.7 (d, *J* = 249.5 Hz), 150.3, 135.7, 133.7 (d, *J* = 8.4 Hz), 129.0, 122.1, 119.0 (d, *J* = 3.5 Hz), 115.7 (d, *J* = 22.1 Hz), 87.5 (d, *J* = 1.3 Hz), 84.2, 57.3, 39.2, 21.3. ^{19}F NMR (376 MHz, $CDCl_3$) δ -110.9. HRMS (ESI/QTOF) m/z: $[M+Na]^+$ Calcd for $C_{18}H_{14}FN_3NaO_2^+$ 346.0962; Found 346.0953.

4-(1-Azido-4-(2-chlorophenyl)but-3-yn-2-yl)phenyl acetate (4p):

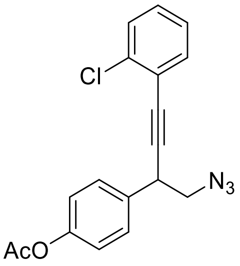

4p

Synthesized following **general procedure D** starting from potassium ((2-chlorophenyl)ethynyl)trifluoroborate (**5i**) (109 mg, 0.450 mmol, 1.50 equiv). The reaction was carried out for 1.5 h, DCM was used for the silica plug. The crude product was purified by column chromatography (pentane/Et_2O, 90:10 to 85:15) to afford 4-(1-azido-4-(2-chlorophenyl)but-3-yn-2-yl)phenyl acetate (**4p**) (79 mg, 0.23 mmol, 77%) as a yellow oil.

R_f (pentane/Et_2O, 8:2): 0.3; ^{1}H NMR (400 MHz, $CDCl_3$) δ 7.56 – 7.47 (m, 3H, Ar*H*), 7.41 (dd, *J* = 7.9, 1.4 Hz, 1H, Ar*H*), 7.30 – 7.17 (m, 2H, Ar*H*), 7.15 – 7.07 (m, 2H, Ar*H*), 4.16 (t, *J* = 6.8 Hz, 1H, C*H*C≡C), 3.66 (dd, *J* = 12.0, 7.6 Hz, 1H, CH_2N_3), 3.56 (dd, *J* = 12.0, 6.3 Hz, 1H, CH_2N_3), 2.30 (s, 3H, CH_3). ^{13}C NMR (101 MHz, $CDCl_3$) δ 169.5, 150.3, 136.3, 135.4, 133.6, 129.5, 129.4, 129.2, 126.6, 122.9, 122.1, 93.2, 82.1, 57.4, 39.3, 21.3. HRMS (ESI/QTOF) m/z: $[M+Na]^+$ Calcd for $C_{18}H_{14}ClN_3NaO_2^+$ 362.0667; Found 362.0654.

Methyl 4-(3-(4-acetoxyphenyl)-4-azidobut-1-yn-1-yl)benzoate (4q):

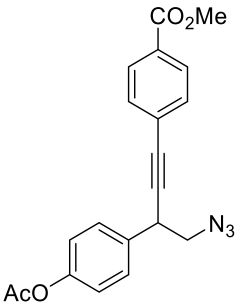

4q

Synthesized following **general procedure D** starting from potassium trifluoro((4-(methoxycarbonyl)phenyl)ethynyl)borate (**5j**) (120 mg, 0.450 mmol, 1.50 equiv). The reaction was carried out for 1.5 h, DCM was used for the silica plug. The crude product was purified by

column chromatography (pentane/EtOAc, 85:15 to 80:20) to afford methyl 4-(3-(4-acetoxyphenyl)-4-azidobut-1-yn-1-yl)benzoate (**4q**) (74 mg, 0.20 mmol, 68%) as a yellow oil.

R_f (pentane/EtOAc, 8:2): 0.33; ^{1}H NMR (400 MHz, $CDCl_3$) δ 8.02 – 7.96 (m, 2H, Ar*H*), 7.55 – 7.51 (m, 2H, Ar*H*), 7.49 – 7.42 (m, 2H, Ar*H*), 7.15 – 7.08 (m, 2H, Ar*H*), 4.17 – 4.11 (m, 1H, C*H*C≡C), 3.92 (s, 3H, OCH_3), 3.63 (dd, *J* = 12.0, 7.7 Hz, 1H, CH_2N_3), 3.53 (dd, *J* = 12.0, 6.1 Hz, 1H, CH_2N_3), 2.31 (s, 3H, CH_3). ^{13}C NMR (101 MHz, $CDCl_3$) δ 169.6, 166.7, 150.4, 135.4, 131.8, 129.8, 129.6, 129.0, 127.6, 122.2, 90.9, 84.5, 57.2, 52.4, 39.3, 21.3. HRMS (ESI/QTOF) m/z: $[M+Na]^+$ Calcd for $C_{20}H_{17}N_3NaO_4^+$ 386.1111; Found 386.1101.

4-(1-Azido-4-mesitylbut-3-yn-2-yl)phenyl acetate (4r):

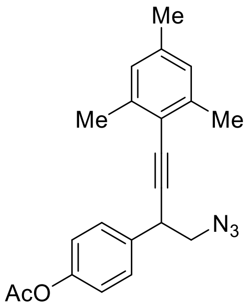

4r

Synthesized following **general procedure D** starting from potassium trifluoro(mesitylethynyl)borate (**5k**) (113 mg, 0.450 mmol, 1.50 equiv). The reaction was carried out for 3 h, DCM was used for the silica plug. The crude product was purified by column chromatography (pentane/Et_2O, 95:5 to 90:10) to afford 4-(1-azido-4-mesitylbut-3-yn-2-yl)phenyl acetate (**4r**) (35 mg, 99 μmol, 33%) as a yellow oil.

R_f (pentane/Et_2O, 9:1): 0.26; ^{1}H NMR (400 MHz, $CDCl_3$) δ 7.53 – 7.48 (m, 2H, Ar*H*), 7.14 – 7.08 (m, 2H, Ar*H*), 6.87 (s, 2H, Ar*H*), 4.20 (t, *J* = 6.9 Hz, 1H, C*H*C≡C), 3.64 (dd, *J* = 12.0, 7.4 Hz, 1H, CH_2N_3), 3.58 (dd, *J* = 12.0, 6.4 Hz, 1H, CH_2N_3), 2.42 (s, 6H, $ArCH_3$), 2.31 (s, 3H, $CH_3C(O)$), 2.28 (s, 3H, $ArCH_3$). ^{13}C NMR (101 MHz, $CDCl_3$) δ 169.5, 150.2, 140.4, 137.8, 136.2, 129.1, 127.7, 122.0, 119.7, 95.2, 83.1, 57.9, 39.3, 21.4, 21.3, 21.2. HRMS (ESI/QTOF) m/z: $[M+Na]^+$ Calcd for $C_{21}H_{21}N_3NaO_2^+$ 370.1526; Found 370.1536.

4-(1-Azido-4-(thiophen-3-yl)but-3-yn-2-yl)phenyl acetate (4s):

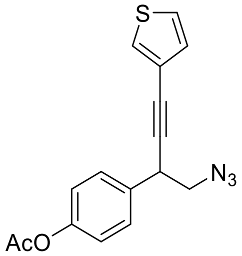

4s

Synthesized following **general procedure D** starting from potassium trifluoro(thiophen-3-ylethynyl)borate (**5l**) (97 mg, 0.45 mmol, 1.50 equiv). The reaction was carried out for 3 h, DCM was used for the silica plug. The crude product was purified by column chromatography (pentane/Et_2O, 85:15) to afford 4-(1-azido-4-(thiophen-3-yl)but-3-yn-2-yl)phenyl acetate (**3ac**) (50 mg, 0.16 mmol, 54%) as a yellow oil.

R_f (pentane/Et_2O, 8:2): 0.29; ^{1}H NMR (400 MHz, $CDCl_3$) δ 7.50 – 7.44 (m, 3H, Ar*H*), 7.28 (dd, *J* = 5.0, 3.0 Hz, 1H, Ar*H*), 7.17 – 7.09 (m, 3H, Ar*H*), 4.14 – 4.08 (m, 1H, C*H*C≡C), 3.61 (dd, *J* = 12.0, 7.7 Hz, 1H, CH_2N$_3$), 3.51 (dd, *J* = 12.0, 6.1 Hz, 1H, CH_2N$_3$), 2.31 (s, 3H, CH_3). ^{13}C NMR (101 MHz, $CDCl_3$) δ 169.5, 150.3, 135.7, 130.0, 129.0, 129.0, 125.4, 122.0, 121.9, 87.3, 80.4, 57.2, 39.2, 21.3. HRMS (ESI/QTOF) m/z: $[M+Na]^+$ Calcd for $C_{16}H_{13}N_3NaO_2S^+$ 334.0621; Found 334.0624.

4-(1-Azido-4-cyclopropylbut-3-yn-2-yl)phenyl acetate (4t):

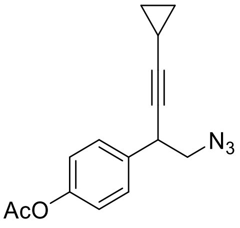

4t

Synthesized following **general procedure D** starting from potassium (cyclopropylethynyl)trifluoroborate (**5o**) (77 mg, 0.45 mmol, 1.50 equiv). The reaction was carried out for 1.5 h, DCM was used for the silica plug. The crude product was purified by column chromatography (pentane/Et_2O, 9:1) to afford 4-(1-azido-4-cyclopropylbut-3-yn-2-yl)phenyl acetate (**4t**) (42 mg, 0.16 mmol, 52%) as a colorless oil.

R_f (pentane/Et_2O, 8:2): 0.29; ^{1}H NMR (400 MHz, $CDCl_3$) δ 7.40 – 7.34 (m, 2H, Ar*H*), 7.09 – 7.04 (m, 2H, Ar*H*), 3.87 – 3.80 (m, 1H, C*H*C≡C), 3.45 (dd, *J* = 11.9, 7.6 Hz, 1H, CH_2N$_3$), 3.36 (dd, *J* = 12.0, 6.1 Hz, 1H, CH_2N$_3$), 2.29 (s, 3H, CH_3), 1.34 – 1.25 (m, 1H, C*H*CH$_2$), 0.81 – 0.68 (m, 4H, CHCH_2). ^{13}C NMR (101 MHz, $CDCl_3$) δ 169.5, 150.1, 136.4, 128.9, 121.8, 88.6, 73.6, 57.5, 38.6, 21.2, 8.1, -0.3. HRMS (ESI/QTOF) m/z: $[M+Na]^+$ Calcd for $C_{15}H_{15}N_3NaO_2^+$ 292.1056; Found 292.1055.

4-(1-Azidobut-3-yn-2-yl)phenyl acetate (4u):

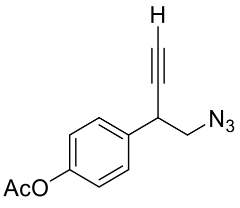

4u

Synthesized following **general procedure D** starting from potassium ethynyltrifluoroborate (**5f**) (59 mg, 0.45 mmol, 1.50 equiv). The reaction was carried out for 1.5 h, DCM was used for the

silica plug. The crude product was purified by column chromatography (pentane/Et_2O, 90:10 to 85:15) to afford 4-(1-azidobut-3-yn-2-yl)phenyl acetate (**4u**) (23 mg, 0.10 mmol, 34%) as a yellow oil.

R_f (pentane/Et_2O, 8:2): 0.35; ^1H NMR (400 MHz, $CDCl_3$) δ 7.44 – 7.39 (m, 2H, Ar*H*), 7.12 – 7.07 (m, 2H, Ar*H*), 3.89 (td, *J* = 7.3, 2.5 Hz, 1H, C*H*C≡C), 3.56 (dd, *J* = 12.0, 7.5 Hz, 1H, C*H₂*N₃), 3.48 (dd, *J* = 12.0, 6.3 Hz, 1H, CH_2N_3), 2.39 (d, *J* = 2.5 Hz, 1H, C≡C*H*), 2.30 (s, 3H, CH_3). ^{13}C NMR (101 MHz, $CDCl_3$) δ 169.5, 150.4, 135.1, 129.0, 122.1, 82.5, 73.3, 57.1, 38.1, 21.3. HRMS (ESI/QTOF) m/z: $[M+Na]^+$ Calcd for $C_{12}H_{11}N_3NaO_2^+$ 252.0743; Found 252.0734.

4-(1-Azido-4-phenylbut-3-yn-2-yl)-1,1'-biphenyl (4v):

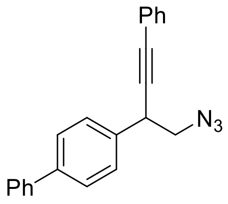

4v

Synthesized following **general procedure C** starting from 4-vinylbiphenyl (54 mg, 0.30 mmol, 1.00 equiv). The reaction was carried out for 1.5 h, DCM was used for the silica plug. The crude product was purified by column chromatography (pentane/toluene, 90:10 to 80:20) to afford 4-(1-azido-4-phenylbut-3-yn-2-yl)-1,1'-biphenyl (**4v**) (74 mg, 0.23 mmol, 76%) as a yellow oil.

R_f (pentane/toluene, 7:3): 0.48; ^1H NMR (400 MHz, $CDCl_3$) δ 7.66 – 7.60 (m, 4H, Ar*H*), 7.58 – 7.51 (m, 4H, Ar*H*), 7.49 – 7.44 (m, 2H, Ar*H*), 7.41 – 7.32 (m, 4H, Ar*H*), 4.19 (t, *J* = 6.6 Hz, 1H, C*H*C≡C), 3.69 (dd, *J* = 12.0, 7.6 Hz, 1H, CH_2N_3), 3.59 (dd, *J* = 12.0, 6.2 Hz, 1H, CH_2N_3). ^{13}C NMR (101 MHz, $CDCl_3$) δ[45] 140.9, 140.7, 137.3, 131.9, 128.9, 128.4, 128.4, 127.7, 127.5, 127.2, 123.1, 88.0, 85.1, 57.3, 39.4. HRMS (ESI/QTOF) m/z: $[M+H]^+$ Calcd for $C_{22}H_{18}N_3^+$ 324.1495; Found 324.1497.

1-(1-Azido-4-phenylbut-3-yn-2-yl)-2-methylbenzene (4w):

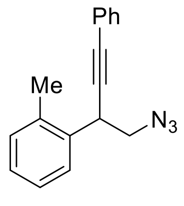

4w

Synthesized following **general procedure C** starting from 2-methylstyrene (39 µL, 0.30 mmol, 1.00 equiv). The reaction was carried out for 1.5 h, DCM was used for the silica plug. The crude product was purified by column chromatography (pentane/toluene, 90:10 to 85:15) to

[45] One aromatic carbon was not resolved.

afford 1-(1-azido-4-phenylbut-3-yn-2-yl)-2-methylbenzene (**4w**) (62 mg, 0.24 mmol, 79%) as a yellow oil.

R_f (pentane/toluene, 7:3): 0.57; ^{1}H NMR (400 MHz, $CDCl_3$) δ 7.65 – 7.61 (m, 1H, Ar*H*), 7.51 – 7.45 (m, 2H, Ar*H*), 7.34 – 7.29 (m, 3H, Ar*H*), 7.29 – 7.18 (m, 3H, Ar*H*), 4.35 (dd, *J* = 8.1, 5.8 Hz, 1H, C*H*C≡C), 3.60 (dd, *J* = 12.0, 8.2 Hz, 1H, CH_2N_3), 3.49 (dd, *J* = 12.0, 5.8 Hz, 1H, CH_2N_3), 2.42 (s, 3H, CH_3). ^{13}C NMR (101 MHz, $CDCl_3$) δ 136.4, 135.4, 131.8, 130.9, 128.4, 128.3, 128.2, 127.8, 126.8, 123.2, 88.7, 84.3, 56.2, 36.2, 19.4. HRMS (Sicrit plasma/LTQ-Orbitrap) m/z: $[M-N_2+H]^+$ Calcd for $C_{17}H_{16}N^+$ 234.1277; Found 234.1277.

1-(1-Azido-4-phenylbut-3-yn-2-yl)-4-(trifluoromethoxy)benzene (4x):

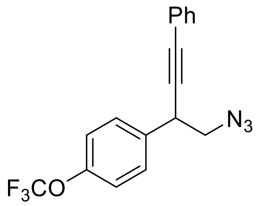

4x

Synthesized following **general procedure C** starting from a solution of 1-(trifluoromethoxy)-4-vinylbenzene (**1r**) (56 mg, 0.30 mmol, 1.00 equiv) in dry degassed DME (0.5 mL). The reaction was carried out for 1.5 h, DCM was used for the silica plug. The crude product was purified by column chromatography (pentane/toluene, 9:1) to afford 1-(1-azido-4-phenylbut-3-yn-2-yl)-4-(trifluoromethoxy)benzene (**4x**) (59 mg, 0.18 mmol, 59%) as a colorless oil.

R_f (pentane/toluene, 85:15): 0.35; ^{1}H NMR (400 MHz, $CDCl_3$) δ 7.52 – 7.46 (m, 4H, Ar*H*), 7.35 – 7.31 (m, 3H, Ar*H*), 7.24 (d, *J* = 8.0 Hz, 2H, Ar*H*), 4.14 (t, *J* = 6.8 Hz, 1H, C*H*C≡C), 3.63 (dd, *J* = 12.0, 7.2 Hz, 1H, CH_2N_3), 3.53 (dd, *J* = 12.0, 6.3 Hz, 1H, CH_2N_3). ^{13}C NMR (101 MHz, $CDCl_3$) δ 148.9 (q, *J* = 1.8 Hz), 137.0, 131.8, 129.4, 128.6, 128.5, 122.8, 121.4, 120.6 (q, *J* = 257.2 Hz), 87.3, 85.5, 57.3, 39.1. ^{19}F NMR (376 MHz, CDCl3) δ -57.9. HRMS (APPI/LTQ-Orbitrap) m/z: $[M-N_2+H]^+$ Calcd for $C_{17}H_{13}F_3NO^+$ 304.0944; Found 304.0946

1-(1-Azido-4-phenylbut-3-yn-2-yl)-3-chlorobenzene (4y):

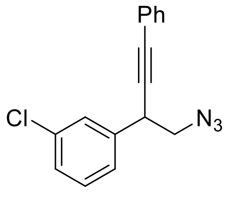

4y

Synthesized following **general procedure C** starting from 3-chlorostyrene (38 µL, 0.30 mmol, 1.00 equiv). The reaction was carried out for 1.5 h, DCM was used for the silica plug. The crude product was purified by column chromatography (pentane/toluene, 90:10 to 85:15) to afford 1-(1-azido-4-phenylbut-3-yn-2-yl)-3-chlorobenzene (**4y**) (45 mg, 0.16 mmol, 53%) as a yellow oil.

R_f (pentane/toluene, 8:2): 0.24; ^1H NMR (400 MHz, $CDCl_3$) δ 7.52 – 7.45 (m, 3H, Ar*H*), 7.37 – 7.28 (m, 6H, Ar*H*), 4.10 (t, *J* = 6.8 Hz, 1H, C*H*C≡C), 3.63 (dd, *J* = 12.0, 7.3 Hz, 1H, CH_2N_3), 3.54 (dd, *J* = 12.0, 6.3 Hz, 1H, CH_2N_3). ^{13}C NMR (101 MHz, $CDCl_3$) δ 140.3, 134.8, 131.9, 130.2, 128.6, 128.5, 128.2, 128.1, 126.2, 122.8, 87.1, 85.5, 57.2, 39.4. HRMS (APPI/LTQ-Orbitrap) m/z: $[M-N_2+H]^+$ Calcd for $C_{16}H_{13}ClN^+$ 254.0731; Found 254.0737.

1-(1-Azido-4-phenylbut-3-yn-2-yl)-4-(trifluoromethyl)benzene (4z):

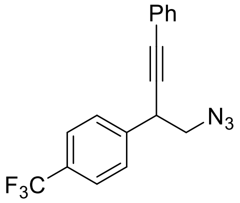

4z

Synthesized following **general procedure C** starting from 4-(trifluoromethyl)styrene (46 µL, 0.30 mmol, 1.00 equiv). The reaction was carried out for 3 h, DCM was used for the silica plug. The crude product was purified by column chromatography (pentane/toluene, 90:10 to 85:15) to afford 1-(1-azido-4-phenylbut-3-yn-2-yl)-4-(trifluoromethyl)benzene (**4z**) (33 mg, 0.11 mmol, 35%) as a yellow oil.

R_f (pentane/toluene, 85:15): 0.38; ^1H NMR (400 MHz, $CDCl_3$) δ 7.65 (d, *J* = 8.3 Hz, 2H, Ar*H*), 7.59 (d, *J* = 8.4 Hz, 2H, Ar*H*), 7.50 – 7.46 (m, 2H, Ar*H*), 7.36 – 7.31 (m, 3H, Ar*H*), 4.18 (t, *J* = 6.7 Hz, 1H, C*H*C≡C), 3.66 (dd, *J* = 12.1, 7.1 Hz, 1H, CH_2N_3), 3.57 (dd, *J* = 12.1, 6.3 Hz, 1H, CH_2N_3). ^{13}C NMR (101 MHz, $CDCl_3$) δ 142.3, 131.9, 130.2 (q, *J* = 32.7 Hz), 128.7, 128.5, 128.5, 125.9 (q, *J* = 3.6 Hz), 124.2 (q, *J* = 272.1 Hz), 122.7, 86.9, 85.7, 57.1, 39.5. ^{19}F NMR (376 MHz, $CDCl_3$) δ -62.6. HRMS (APPI/LTQ-Orbitrap) m/z: $[M-N_2+H]^+$ Calcd for $C_{17}H_{13}F_3N^+$ 288.0995; Found 288.0995.

2-(1-Azido-4-phenylbut-3-yn-2-yl)benzofuran (4aa):

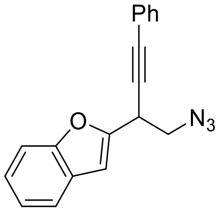

4aa

Synthesized following **general procedure C** starting from a solution of 2-vinylbenzofuran (**1u**) (43 mg, 0.30 mmol, 1.00 equiv) in dry degassed DME (0.5 mL). The reaction was carried out for 1.5 h, DCM was used for the silica plug. The crude product was purified by column chromatography (pentane/toluene, 85:15) to afford 2-(1-azido-4-phenylbut-3-yn-2-yl)benzofuran (**4aa**) (54 mg, 0.19 mmol, 62%) as a yellow oil.

R_f (pentane/toluene, 8:2): 0.35; ^1H NMR (400 MHz, $CDCl_3$) δ 7.59 – 7.45 (m, 4H, Ar*H*), 7.37 – 7.32 (m, 3H, Ar*H*), 7.33 – 7.19 (m, 2H, Ar*H*), 6.84 (s, 1H, OC=C*H*), 4.37 (t, *J* = 6.0 Hz, 1H,

CHC≡C), 3.83 (dd, J = 11.1, 4.7 Hz, 1H, CH_2N$_3$), 3.79 (dd, J = 12.1, 4.9 Hz, 1H, CH_2N$_3$). ^{13}C NMR (101 MHz, $CDCl_3$) δ 155.2, 153.9, 132.0, 128.7, 128.5, 128.3, 124.4, 123.1, 122.6, 121.1, 111.3, 105.0, 84.9, 84.8, 54.1, 34.2. HRMS (APPI/LTQ-Orbitrap) m/z: $[M-N_2+H]^+$ Calcd for $C_{18}H_{14}NO^+$ 260.1070; Found 260.1071.

4-(1-Azido-4-(3-methoxyphenyl)but-3-yn-2-yl)phenol (4ab):

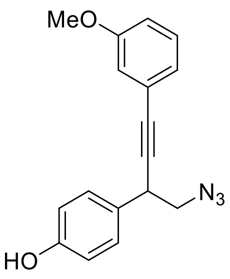

4ab

An oven-dried test tube charged with $Ru(bpy)_3(PF_6)_2$ (5.2 mg, 6.0 μmol, 2 mol%), Ts-ABZ (166 mg, 0.375 mmol, 1.25 equiv), potassium trifluoro((3-methoxyphenyl)ethynyl)borate (**5g**) (107 mg, 0.450 mmol, 1.50 equiv) and 4-vinylphenol (**1t**) (36 mg, 0.30 mmol, 1.00 equiv) was evacuated and backfilled with N_2 (3×). Dry degassed DME (2.7 mL) was added and the mixture was cooled to −20 °C. Then, a stock solution of BF_3•Et_2O (11.1 μL, 90.0 μmol, 0.30 equiv) in dry degassed DME (0.34 mL) was added and the reaction was stirred under blue LEDs irradiation (1 × Kessil 467 nm 50% intensity, 22W) at −20 °C for 1.5 h. The reaction mixture was filtered through a short plug of silica and eluted with DCM then concentrated in vacuo. The crude product was purified by column chromatography (pentane/EtOAc, 85:15 to 80:20) to afford 4-(1-azido-4-(3-methoxyphenyl)but-3-yn-2-yl)phenol (**4ab**) (41 mg, 0.14 mmol, 47%) as an orange oil.

R_f (pentane/EtOAc, 75:25): 0.32; ^{1}H NMR (400 MHz, $CDCl_3$) δ 7.35 – 7.30 (m, 2H, Ar*H*), 7.25 – 7.19 (m, 1H Ar*H*), 7.11 – 7.06 (m, 1H Ar*H*), 7.02 (dd, J = 2.4, 1.4 Hz, 1H Ar*H*), 6.89 (ddd, J = 8.4, 2.6, 0.8 Hz, 1H Ar*H*), 6.87 – 6.81 (m, 2H Ar*H*), 5.19 (bs, 1H, O*H*), 4.06 (t, J = 6.9 Hz, 1H, C*H*C≡C), 3.80 (s, 3H, OCH_3), 3.59 (dd, J = 12.0, 7.5 Hz, 1H, CH_2N$_3$), 3.49 (dd, J = 12.0, 6.2 Hz, 1H, CH_2N$_3$). ^{13}C NMR (101 MHz, $CDCl_3$) δ 159.4, 155.2, 130.4, 129.5, 129.2, 124.4, 124.1, 116.6, 115.8, 115.0, 88.2, 84.8, 57.5, 55.4, 38.9. HRMS (APPI/LTQ-Orbitrap) m/z: $[M-N_2+H]^+$ Calcd for $C_{17}H_{16}NO_2^+$ 266.1176; Found 266.1176.

(4-Azidopent-1-yne-1,3-diyl)dibenzene (4ac):

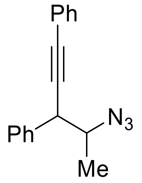

4ac

Synthesized following **general procedure C** starting from *trans*-β-methylstyrene (36 μL, 0.30 mmol, 1.00 equiv). The reaction was carried out for 1.5 h, DCM was used for the silica plug.

Crude ^{1}H NMR of the mixture showed a diastereomeric ratio of 1.9:1 using peaks at 4.08 and 3.98 ppm. The crude product was purified by column chromatography (pentane/Et_2O, 99:1 to 98:2) to afford an inseparable mixture of diastereoisomers (4-azidopent-1-yne-1,3-diyl)dibenzene (**4ac**) (45 mg, 0.17 mmol, 57%) as a yellow oil.

R_f (pentane/Et_2O, 97.5:2.5): 0.54; ^{1}H NMR (400 MHz, $CDCl_3$) δ 7.53 – 7.44 (m, 8H, Ar*H* major + minor), 7.42 – 7.36 (m, 4H, Ar*H* major + minor), 7.35 – 7.30 (m, 8H, Ar*H* major + minor), 4.08 (d, *J* = 6.1 Hz, 1H, C*H*C≡C major), 3.98 (d, *J* = 5.4 Hz, 1H, C*H*C≡C minor), 3.83 – 3.75 (m, 1H, C*H*N_3 minor), 3.71 (p, *J* = 6.5 Hz, 1H, C*H*N_3 major), 1.40 (d, *J* = 6.6 Hz, 3H, C*H₃* major), 1.38 (d, *J* = 6.7 Hz, 3H, C*H₃* minor). ^{13}C NMR (101 MHz, $CDCl_3$) δ 138.1, 138.1, 131.8, 131.8, 128.8, 128.7, 128.6, 128.5, 128.4, 128.4, 128.3, 128.3, 127.8, 127.7, 123.2, 123.2, 87.6, 87.4, 85.7, 85.4, 62.1, 62.0, 45.4, 45.2, 17.7, 16.0. HRMS (APPI/LTQ-Orbitrap) m/z: $[M-N_2+H]^+$ Calcd for $C_{17}H_{16}N^+$ 234.1277; Found 234.1288.

2-Azido-1-(phenylethynyl)-2,3-dihydro-1*H*-indene (4ad/4ad'):

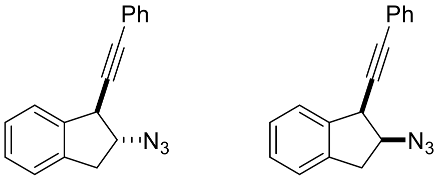

4ad **4ad'**

Synthesized following **general procedure C** starting from indene (35 µL, 0.30 mmol, 1.00 equiv). The reaction was carried out for 1.5 h, DCM was used for the silica plug. Crude ^{1}H NMR of the mixture showed a diastereomeric ratio of 5.4:1 (*trans*:*cis*) using peaks at 4.46 and 4.38 ppm. The crude product was purified by column chromatography (pentane/toluene, 9:1 to 8:2).

trans diastereoisomer (major): 1,2-*trans*-2-azido-1-(phenylethynyl)-2,3-dihydro-1*H*-indene (**4ad**) (49 mg, 0.19 mmol, 63%) yellow oil. The *trans* configuration was determined using ^{1}H-^{1}H NOESY experiment on the reduced product.**Fehler! Textmarke nicht definiert.**

R_f (pentane/toluene, 7:3): 0.46; ^{1}H NMR (400 MHz, $CDCl_3$) δ 7.50 – 7.42 (m, 3H, Ar*H*), 7.35 – 7.21 (m, 6H, Ar*H*), 4.38 (q, *J* = 8.3 Hz, 1H, C*H*N_3), 4.22 (d, *J* = 8.1 Hz, 1H, C*H*C≡C), 3.32 (dd, *J* = 15.5, 7.4 Hz, 1H, ArC*H₂*), 2.95 (dd, *J* = 15.5, 8.6 Hz, 1H, ArC*H₂*). ^{13}C NMR (101 MHz, $CDCl_3$) δ 140.5, 138.7, 131.9, 128.4, 128.3, 128.1, 127.7, 124.8, 124.6, 123.1, 88.0, 83.9, 68.7, 43.7, 37.4. HRMS (APPI/LTQ-Orbitrap) m/z: $[M-N_2]^+$ Calcd for $C_{17}H_{13}N^+$ 231.1043; Found 231.1043.

cis diastereoisomer (minor): 1,2-*cis*-2-azido-1-(phenylethynyl)-2,3-dihydro-1*H*-indene (**4ad'**), the yield was determined only by crude ^{1}H NMR (12%) using CH_2Br_2 (10.6 µL, 0.150 mmol, 0.50 equiv) as internal standard. An analytically pure sample was obtained by preparative TLC (pentane/toluene, 1:1).

R_f (pentane/toluene, 7:3): 0.25; ^{1}H NMR (400 MHz, $CDCl_3$) δ 7.54 – 7.47 (m, 3H, Ar*H*), 7.34 – 7.30 (m, 3H, Ar*H*), 7.28 – 7.25 (m, 3H, Ar*H*), 4.49 – 4.43 (m, 2H, C*H*C≡C + C*H*N_3), 3.26 – 3.16

(m, 1H, ArC*H₂*), 3.13 – 3.06 (m, 1H, ArC*H₂*). ^{13}C NMR (101 MHz, $CDCl_3$) δ 140.5, 139.3, 132.0, 128.4, 128.3, 128.0, 127.7, 124.9, 124.7, 123.2, 85.6, 85.5, 65.4, 43.6, 38.1. HRMS (nanochip-ESI/LTQ-Orbitrap) m/z: $[M-N_2+H]^+$ Calcd for $C_{17}H_{14}N^+$ 232.1121; Found 232.1122.

3,4-*trans*-3-Azido-4-(phenylethynyl)chromane (4ae):

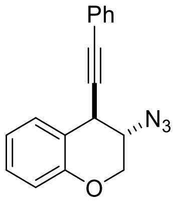

4ae

Synthesized following **general procedure C** starting from a solution of 2*H*-chromene (**1w**) (40 mg, 0.30 mmol, 1.00 equiv) in dry degassed DME (0.5 mL). The reaction was carried out for 1.5 h, DCM was used for the silica plug. The crude product was purified by column chromatography (pentane/toluene, 85:15 to 80:20) to afford 3,4-*trans*-3-azido-4-(phenylethynyl)chromane (**4ae**) (24 mg, 0.090 mmol, 30%) as a yellow oil. Relative configuration of the diastereoisomer was determined by analogy to **4ad/4ad'** where the major product is *trans*.

R_f (Pentane/Toluene, 75:25): 0.23; ^{1}H NMR (400 MHz, $CDCl_3$) δ 7.49 – 7.42 (m, 3H, Ar*H*), 7.34 – 7.29 (m, 3H, Ar*H*), 7.25 – 7.18 (m, 1H, Ar*H*), 6.99 (td, *J* = 7.5, 1.2 Hz, 1H, Ar*H*), 6.88 (dd, *J* = 8.2, 1.1 Hz, 1H, Ar*H*), 4.43 (dd, *J* = 10.9, 2.0 Hz, 1H, OCH_2CH), 4.13 – 4.01 (m, 3H, OCH_2CH + C*H*N_3 + C*H*C≡C). ^{13}C NMR (101 MHz, $CDCl_3$) δ 152.8, 131.9, 130.0, 129.1, 128.6, 128.4, 122.7, 121.7, 119.3, 117.0, 87.9, 84.4, 65.9, 58.3, 35.2. HRMS (APPI/LTQ-Orbitrap) m/z: $[M-N_2+H]^+$ Calcd for $C_{17}H_{14}NO^+$ 248.1070; Found 248.1080.

4-(1-Azido-4-(benzofuran-2-yl)but-3-yn-2-yl)phenyl acetate (4af):

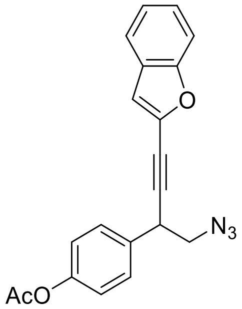

4af

Synthesized following **general procedure D** starting from potassium (benzofuran-2-ylethynyl)trifluoroborate (**5m**) (112 mg, 0.450 mmol, 1.50 equiv). The reaction was carried out for 3 h, DCM was used for the silica plug. The crude product was purified by column chromatography (pentane/Et_2O, 8:2) to afford 4-(1-azido-4-(benzofuran-2-yl)but-3-yn-2-yl)phenyl acetate (**4af**) (49 mg, 0.14 mmol, 47%) as a yellow oil.

R_f (pentane/Et_2O, 6:4): 0.45; ^1H NMR (400 MHz, $CDCl_3$) δ 7.55 (d, *J* = 7.8 Hz, 1H, Ar*H*), 7.46 (t, *J* = 8.3 Hz, 3H, Ar*H*), 7.33 (t, *J* = 7.2 Hz, 1H, Ar*H*), 7.24 (t, *J* = 7.5 Hz, 1H, Ar*H*), 7.12 (d, *J* = 8.6 Hz, 2H, Ar*H*), 6.95 (s, 1H, OC=C*H*), 4.18 (t, *J* = 6.9 Hz, 1H, C*H*C≡C), 3.68 (dd, *J* = 12.0, 7.5 Hz, 1H, CH_2N_3), 3.58 (dd, *J* = 12.0, 6.3 Hz, 1H, CH_2N_3), 2.31 (s, 3H, CH_3). ^{13}C NMR (101 MHz, $CDCl_3$) δ 169.5, 154.8, 150.5, 138.3, 134.7, 129.1, 127.6, 125.7, 123.4, 122.2, 121.4, 111.8, 111.4, 94.1, 75.7, 56.8, 39.2, 21.3. HRMS (ESI/QTOF) m/z: $[M+Na]^+$ Calcd for $C_{20}H_{15}N_3NaO_3^+$ 368.1006; Found 368.1022.

4-(1-Azidopent-3-yn-2-yl)phenyl acetate (4ag)

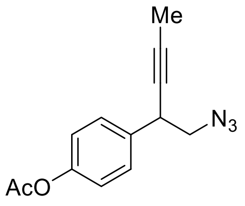

4ag

Synthesized following **general procedure D** starting from potassium trifluoro(prop-1-yn-1-yl)borate (**5n**) (66 mg, 0.45 mmol, 1.50 equiv). The reaction was carried out for 3 h, DCM was used for the silica plug. The crude product was purified by column chromatography (pentane/Et_2O, 8:2) to afford 4-(1-azidopent-3-yn-2-yl)phenyl acetate (**4ag**) (40 mg, 0.16 mmol, 54%) as a slightly yellow oil.

R_f (pentane/Et_2O, 8:2): 0.46; ^1H NMR (400 MHz, $CDCl_3$) δ 7.41 – 7.36 (m, 2H, Ar*H*), 7.10 – 7.04 (m, 2H, Ar*H*), 3.87 – 3.80 (m, 1H, C*H*C≡C), 3.47 (dd, *J* = 11.9, 7.6 Hz, 1H, CH_2N_3), 3.39 (dd, *J* = 11.9, 6.2 Hz, 1H, CH_2N_3), 2.29 (s, 3H, CH_3), 1.88 (d, *J* = 2.4 Hz, 3H, C≡CCH_3). ^{13}C NMR (101 MHz, $CDCl_3$) δ[46] 169.5, 150.1, 136.3, 128.9, 121.9, 81.1, 57.5, 38.5, 21.2, 3.7. HRMS (ESI/QTOF) m/z: $[M+Na]^+$ Calcd for $C_{13}H_{13}N_3NaO_2^+$ 266.0900; Found 266.0902.

4-(1-Azido-7-chlorohept-3-yn-2-yl)phenyl acetate (4ah):

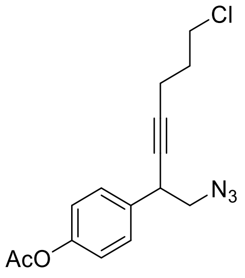

4ah

Synthesized following **general procedure D** starting from potassium (5-chloropent-1-yn-1-yl)trifluoroborate (**5p**) (94 mg, 0.45 mmol, 1.50 equiv). The reaction was carried out for 1.5 h, DCM was used for the silica plug. The crude product was purified by column chromatography

[46] One carbon of the alkyne is overlapping with the $CDCl_3$ signal.

(pentane/Et_2O, 90:10 to 85:15) to afford 4-(1-azido-7-chlorohept-3-yn-2-yl)phenyl acetate (**4ah**) (47 mg, 0.16 mmol, 52%) as a yellow oil.

R_f (pentane/Et_2O, 8:2): 0.2; ^{1}H NMR (400 MHz, $CDCl_3$) δ 7.41 – 7.35 (m, 2H, Ar*H*), 7.10 – 7.05 (m, 2H, Ar*H*), 3.90 – 3.83 (m, 1H, C*H*C≡C), 3.66 (t, *J* = 6.3 Hz, 2H, C*H₂*Cl), 3.47 (dd, *J* = 12.0, 7.7 Hz, 1H, CH_2N_3), 3.40 (dd, *J* = 12.0, 6.1 Hz, 1H, CH_2N_3), 2.46 (td, *J* = 6.9, 2.2 Hz, 2H, C≡CCH_2CH_2), 2.30 (s, 3H, CH_3), 2.00 (p, *J* = 6.6 Hz, 2H, C≡CCH_2CH_2). ^{13}C NMR (101 MHz, $CDCl_3$) δ 169.5, 150.1, 136.1, 128.9, 121.9, 83.6, 79.5, 57.5, 43.8, 38.5, 31.4, 21.2, 16.4. HRMS (ESI/QTOF) m/z: $[M+Na]^+$ Calcd for $C_{15}H_{16}ClN_3NaO_2^+$ 328.0823; Found 328.0830.
